# Immune signatures of protective spleen memory CD8 T cells

**DOI:** 10.1038/srep37651

**Published:** 2016-11-24

**Authors:** Lilia Brinza, Sophia Djebali, Martine Tomkowiak, Julien Mafille, Céline Loiseau, Pierre-Emmanuel Jouve, Simon de Bernard, Laurent Buffat, Bruno Lina, Michèle Ottmann, Manuel Rosa-Calatrava, Stéphane Schicklin, Nathalie Bonnefoy, Grégoire Lauvau, Morgan Grau, Mélanie Wencker, Christophe Arpin, Thierry Walzer, Yann Leverrier, Jacqueline Marvel

**Affiliations:** 1 CIRI, Centre International de Recherche en Infectiologie, Inserm, U1111, Université Claude Bernard Lyon 1, CNRS, UMR5308, École Normale Supérieure de Lyon, Univ Lyon, F-69007, LYON, France; 2 AltraBio SAS, 69007 Lyon, France; 3 Laboratoire Virpath EA4610, Faculté de Médecine Lyon Est, Université Claude Bernard Lyon 1, Université de Lyon, France; 4 Laboratoire de Virologie, CNR des virus influenza, Hospices Civils de Lyon, Lyon, France; 5 IRCM, Institut de Recherche en Cancérologie de Montpellier; INSERM, U896; Université Montpellier 1; CRLC Val d’Aurelle Paul Lamarque, Montpellier, France; 6 Albert Einstein College of Medicine, Department of Microbiology and Immunology, Bronx, NY 10461, USA

## Abstract

Memory CD8 T lymphocyte populations are remarkably heterogeneous and differ in their ability to protect the host. In order to identify the whole range of qualities uniquely associated with protective memory cells we compared the gene expression signatures of two qualities of memory CD8 T cells sharing the same antigenic-specificity: protective (Influenza-induced, Flu-TM) and non-protective (peptide-induced, TIM) spleen memory CD8 T cells. Although Flu-TM and TIM express classical phenotypic memory markers and are polyfunctional, only Flu-TM protects against a lethal viral challenge. Protective memory CD8 T cells express a unique set of genes involved in migration and survival that correlate with their unique capacity to rapidly migrate within the infected lung parenchyma in response to influenza infection. We also enlighten a new set of poised genes expressed by protective cells that is strongly enriched in cytokines and chemokines such as *Ccl1*, *Ccl9* and *Gm-csf*. *CCL1* and *GM-CSF* genes are also poised in human memory CD8 T cells. These immune signatures are also induced by two other pathogens (vaccinia virus and *Listeria monocytogenes*). The immune signatures associated with immune protection were identified on circulating cells, i.e. those that are easily accessible for immuno-monitoring and could help predict vaccines efficacy.

Upon infection or immunization, antigen-specific naive CD8 T lymphocytes proliferate and differentiate into effector cells reactive against pathogen-associated antigens. After the resolution of infection, a minority of cells persist as memory cells. Memory CD8 T lymphocytes differ from naive cells by their improved effector functions but also by their recirculation pattern^1^,^2^. These properties are acquired by effector cells generated during the primary response and are maintained by active gene transcription or by epigenetic modifications^3^. The heterogeneity of memory cells has been recognised long ago and their subdivision in TCM and TEM subsets, on the basis of surface markers, has contributed to their characterisation in terms of differentiation and functional capacities^4^. More recently, a subset of memory cells (TRM) able to persist within most tissues has attracted much attention as they act as sentinels to provide immediate protection upon local secondary infection^5^,^6^. However, the diversity of cells expressing a classical memory surface phenotype and increased functional capacities is reported to be much larger, with a large fraction of them being present in lymphoid organs and blood^7^,^8^.

The identification, for lymphoid organ-derived memory cells, of immune signatures associated with protection is paramount as it would allow the easy screening of vaccine efficiency on blood derived cells. As a result, most studies trying to find a correlation between CD8-mediated-protection and the phenotype, functional capabilities or transcriptome of memory cells have focused on circulating cells as well as cells from lymphoid organs^9^,^10^. Altogether, those findings have demonstrated that the frequency of polyfunctional memory cells, i.e. memory T cells that displayed multiple effector functions such as cytokines, chemokine secretion or cytolytic activity markers, was a better predictor of protection than the frequency of IFN-γ-producing T cells, a parameter frequently used to assess memory cells frequencies^11^. A number of studies have also highlighted the crucial role of functional avidity as a determinant of T cell efficacy^12^. More recently, memory cells quality was evaluated by a microarray approach looking at the genes that were transcribed in quiescent *ex vivo* memory cells generated in different infectious contexts: different pathogens^13^ or type of infection^14^. Memory cells have also been analysed in response to tumour^15^, at different locations following pathogen infection^16^,^17^ or after stimulation^18^. However, none of these studies has addressed the gene expression signature uniquely associated with protection.

Protective memory cells are normally generated in response to acute viral infection, but this is not always achieved when using recombinant vaccines or peptide immunisation^19^. A broad comparison of the memory cells induced by these two types of immunization opens an avenue for identifying genes that can be associated with protection. In order to characterise transcriptomic properties of protective memory cells, we have compared memory CD8 cells generated in response to NP68 encoding influenza virus (Flu-TM) to T Inflammatory memory (TIM) cells that were generated by NP68 peptide in a sterile inflammatory context^20^,^21^,^22^. Since high affinity TCRs have been associated with memory response efficiency, we generated memory cell types using naive CD8 T cells harbouring the same antigenic specificity, using F5 TCR transgenic cells that are specific for NP68, an influenza nucleoprotein epitope. This system avoids the selection of CD8 T cell clones with different affinities in the different experimental models. TIM CD8 T cells display classic phenotypic and functional memory traits^20^,^21^,^22^. We have compared the capacity of these two subsets of memory cells to protect against a lethal dose of influenza virus. Flu-TM CD8 T cells transferred to naive mice are able to protect them in contrast to naive or TIM memory cells. In order to identify genes that are associated with the protective capacity of Flu-TM, we have performed gene expression analysis on quiescent and re-stimulated memory CD8 T cells. We have identified gene signatures that are uniquely associated with Flu-TM and which correlate with their capacity to migrate more rapidly to the infected parenchyma and contain cytokines and chemokines such as CCL1, CCL9 and GM-CSF.

## Results

### Comparison of protective capacity between two memory CD8 T cell populations

**Figure 1:**
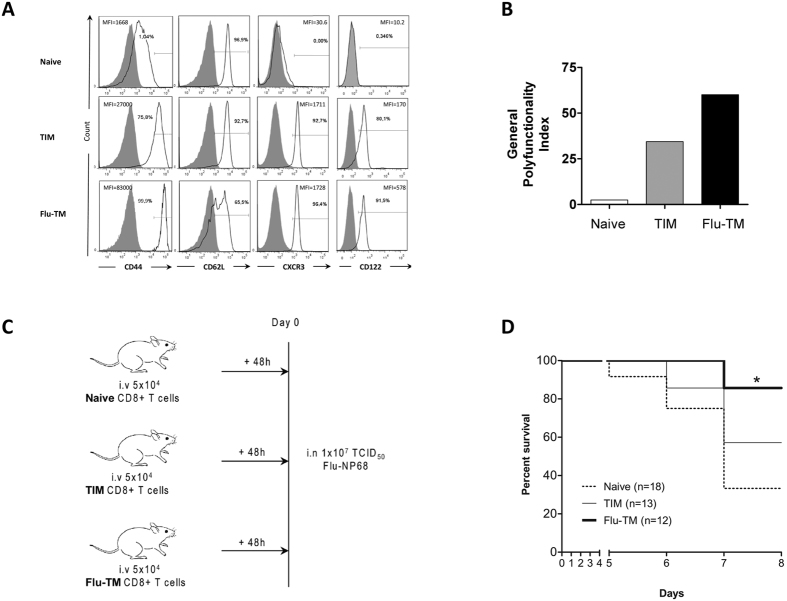
Comparison of phenotype and protective capacity between two memory CD8 T cell populations. (**A**) Naive, TIM and Flu-TM CD8 T cells were stained with the indicated antibodies (black histogram) or isotype control (filled gray histogram). Data show expression on gated F5 CD8 T cells and are representative of five independent experiments. (**B**) Polyfunctionality index (calculated as described in the Methods) of specific CD8 T cells was assessed for the simultaneous production of 4 cytokines: IFN-γ, IL-2, CCL3 and CCL5. Data show one out of five independent experiments with similar results. (**C**) Experimental plan and (**D**) survival curves of infected mice. Mice were transferred with 5 × 10^4^ naive or TIM or Flu-TM CD8 T cells and infected intranasally 48 hours later with a lethal dose of Flu-NP68 (1 × 10^7^ TCID_50_). Data are mean of 2 independent experiments. Total number of mice is indicated in brackets. Survival was significantly improved in the Flu-TM group as compared with naive group (*p < 0.05 Log-rank test).

We first compared the phenotype and the protective capacity of two populations of memory cells generated using naive F5 TCR transgenic cells that are specific for NP68 epitope. Antigen-specific TIM cells were generated in a sterile inflammatory context by direct immunization of F5 TCR transgenic mice with the antigenic NP68 peptide in saline^20^,^21^,^22^. Flu-induced memory cells (Flu-TM) were generated by intranasal infection with influenza virus expressing the NP68 epitope of C57BL/6 J mice that were grafted with 2 × 10^5^ naive F5 CD8 T cells. Although this represents a substantial number of transferred naive CD8 T cells, the initial frequency of naive TCR-transgenic CD8 T cells does not influence functional properties of memory T cells and their ability to protect from re-challenge^13^,^23^. In terms of phenotype and cytokine secretion capacity, TIM memory cells, similarly to Flu-TM F5 cells, display a prototypic memory phenotype expressing increased levels of CD44, CXCR3 and CD122 that distinguish them from naive cells (Fig. 1A) and show significant polyfunctionality (Fig. 1B). We next measured the capacity of the different subsets of CD8 T cells to protect mice against a lethal dose of influenza virus. Naive C57BL/6 J mice were grafted with identical numbers of splenic naive, TIM or Flu-TM F5 CD8 T cells before being intra-nasally infected with a lethal dose of influenza virus (Fig. 1C). Results in Fig. 1D show that the transfer of Flu-TM confers a significant protection against a lethal dose of virus, compared to naive mice. This is in contrast to what is observed with TIM F5 cells. Hence, although TIM and Flu-TM share a memory phenotype, only Flu-TM cells display intrinsic functional capacities, acquired following their priming by the virus and allowing them to curtail a lethal infection by influenza virus.

### Identification of gene sets that distinguish naive, TIM and Flu-TM CD8 T cells

**Figure 2:**
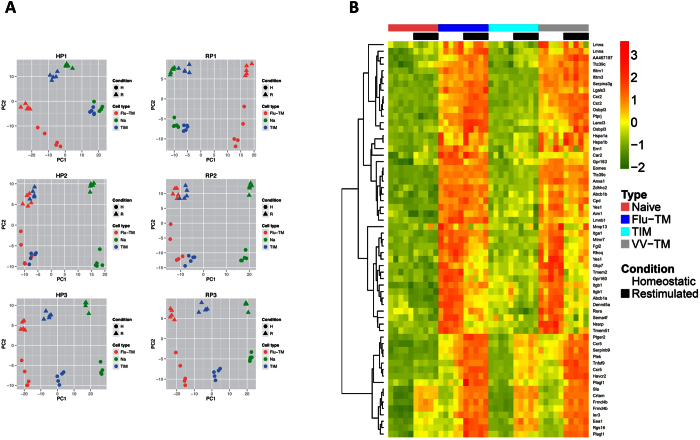
Discretized comparison defined gene clusters that discriminate naive, TIM and Flu-TM cells. (**A**) Principal component analysis of probe sets belonging to the P1, P2 or P3 gene lists defined by the discretized comparison of gene expression profiles in homeostatic (H) or re-stimulated (R) conditions. For each probe set, the complete gene expression profile i.e. expression value in each condition (homeostatic and re-stimulated) has been used for the PCA analysis. (**B**) Expression heatmap of the top 55 HP1 genes with a minimal FC of 4 when comparing Flu-TM to naive cells. Expression profiles in naive cells, Flu-TM, TIM and VV-TM in homeostatic conditions and following restimulation are shown. Colors represent the centered and scaled signal intensity.

In order to identify, at the gene expression level, signatures associated with immune protection, we performed a global transcriptional analysis of naive F5, TIM and Flu-TM F5 CD8 T cells. Arrays were performed with RNA from quiescent (Homeostatic condition, H) or NP68 peptide-stimulated (re-stimulated condition, R) CD8 T cells. This last condition allows the capture of poised genes that are more rapidly expressed by memory cells. Differentially expressed gene numbers are given in Supplementary Table S1. The large majority of genes differentially expressed compared to naive cells in TIM are also differentially expressed in Flu-TM, in both homeostatic and re-stimulated conditions (data not shown). We searched for significantly differentially expressed probe sets in a three way discretized comparison (see Methods). Three groups of genes that discriminate naive cells, TIM and Flu-TM from each other were identified: P1 (naive = TIM < Flu-TM), P2 (naive < TIM = Flu-TM) and P3 (naive < TIM < Flu-TM) (Supplementary Table S2). A principal component analysis (Fig. 2A) performed on the different array-samples, using the P1 to P3 gene lists (Supplementary Tables S3–8), illustrates how P3 and P2 genes discriminate naive cells from memory TIM and Flu-TM in contrast to P1 genes that segregates TIM and naive cells apart from Flu-TM. This is true for gene lists obtained when comparing gene expression by CD8 T cells in homeostatic conditions (H) or following re-stimulation (R). To assess the conserved expression of these memory gene signatures, we performed a meta-analysis of 8 datasets comparing naive and memory CD8 T cells gene-expression-profiles in homeostatic conditions (Supplementary Tables S9–11). Memory CD8 T cells, in these models, were generated using 4 different pathogens (vaccinia virus, *Listeria monocytogenes*, Vesicular Stomatitis Virus and Lymphocytic Choriomeningitis Virus) and 3 different TCR transgenic mice (P14, F5 and OT1). The majority of genes in the P2 and P3 clusters are present in the meta-analysis i.e. are differentially expressed by memory cells compared to naive cells when all these different models are analysed with Rankprod (Table 1 and Supplementary Tables S3–5 and S9). In homeostatic conditions the HP2 and HP3 gene-lists that differentiate memory cells, TIM or Flu-TM, from naive cells contain genes that are classically associated with memory such as *Klrc1*, *Ccl5*, *Cd44*, *Cxcr3* (HP3 profile) or *Ifng*, *Asns* (HP2 profile) (Table 1). The pattern of expression of some of these genes was validated at RNA level (Supplementary Fig. S1).

### Genes differentially expressed by Flu-TM in homeostatic conditions (HP1) are associated with their increased capacity to rapidly access the lung parenchyma

**Figure 3:**
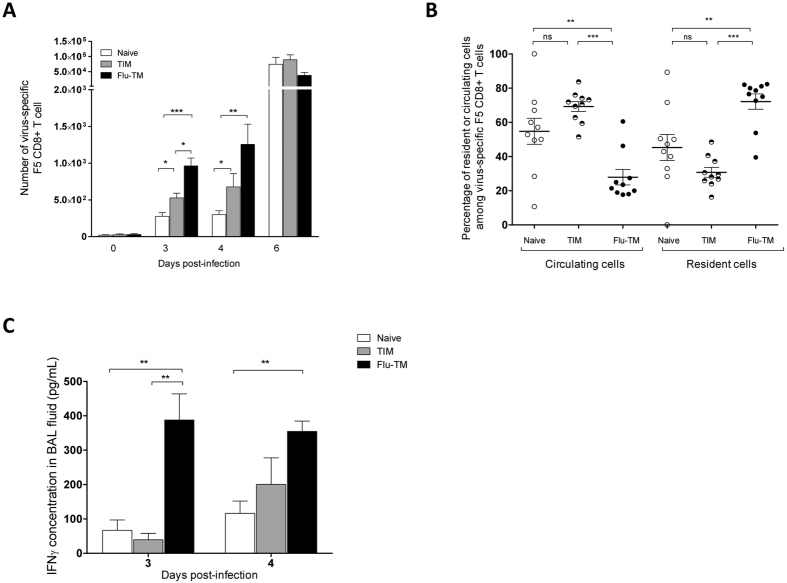
Flu-TM localise more rapidly to the infected lung parenchyma. (**A**) Number of virus-specific F5 CD8 T cells (CD45.1+) in the lung. Mice were transferred with 5 × 10^4^ naive, TIM or Flu-TM CD8 T cells and infected intranasally 48 hours later with Flu-NP68 (2 × 10^5^ TCID_50_). Data correspond to mean cell numbers ± SD with at least four mice per group. One out of 6 independent experiments with similar results is shown. (**B**) Lung parenchyma (resident) or vascular (circulating) virus-specific F5 CD8 T cells were identified at day 4 using anti-CD45 intra-vascular staining. Data are mean of 2 independent experiments with five mice per group. (**C**) IFN-γ concentration in cell-free BAL fluid. Data represent mean ± SD of 4 or 5 mice per group. One out of 4 independent experiment with similar results is shown. *p < 0.05, **p < 0.01 and ***p < 0.001 (two tailed unpaired t-test).

To identify genes whose expression levels could uniquely be associated with spleen Flu-TM CD8 and could represent a signature for protection against a lethal intranasal infection, we next concentrated our analysis on genes that have a P1 profile in homeostatic conditions (HP1) i.e. a significant increased expression in Flu-TM, but not TIM, compared to quiescent naive cells. These HP1 genes are also up-regulated in F5 memory cells generated in response to two other pathogens vaccinia-NP68 (VV-NP68) and *Listeria Monocytogenes*-NP68 (LM-NP68) (Fig. 2B and Supplementary Fig. S2), indicating that this HP1 signature was not restricted to influenza-generated memory CD8 T cells. Genes in this signature code for proteins that are viral infection restricting factors (e.g. *Ifitm1*, *Ifitm3*, *Gbp7*), that protect against cellular or environmental stress (e.g. *Abcb1a*, *Car2*, *Ern1*, *Serpina3g*) or that regulate immune activation processes (e.g. *Fgl2*, *Anxa1*, *Havcr2*). This highlights the balance between genes that protect CD8 T cells from their environment and those that control the potential harmful activity of CD8 T cells for their environment. Genes involved in the control of cell migration were also represented in the HP1 list (e.g. *Ccr2*, *Ccr5*, *Itga1*, *Itgb1*, *Gpr183*, *Rgs16*), with some having an essential role in the recruitment of CD8 T cells in the lung following infection by a pathogen. Indeed, the chemokine receptor CCR5 has been shown to be necessary for the recruitment of peripheral memory CD8 T cells to the lung^24^ and the *Itga1*/*Itgb1* genes code for VLA1, an integrin involved in the retention^25^ and survival^26^ of CD8 memory cells in the lung. To validate if this differential expression could correlate with different migration patterns following a virus induced stimulation, we transferred equivalent numbers of naive, TIM or Flu-TM F5 CD8 cells in naive C57BL/6 J hosts that were subsequently infected intra-nasally with an infectious dose of influenza. The number of F5 cells in different organs was monitored at different times following infection (Fig. 3 and Supplementary Fig. S3). As expected, increased numbers of F5 cells were found in all groups on day 6 post-infection, indicating that at the peak of the response the different subsets of transferred F5 cells were activated and had a similar distribution. This observation was considerably different at earlier time points (day 3 and 4) following infection, when an increased number of F5 CD8 T cells were found in the lung of mice having been transferred with Flu-TM CD8 T cells compared to naive or TIM recipient mice (Fig. 3A). Of note, the number of TIM in the lung, although lower than Flu-TM, was also significantly increased compared to the number of naive F5 cells. However, to discriminate between intravascular and tissue location, we performed *in vivo* anti-CD45 intravascular staining before mice sacrifice, on day 4 following infection^27^. The results show that the majority of Flu-TM are localized within the tissue in contrast to TIM and naive F5 cells that were mainly found in the vasculature (Fig. 3B), indicating that, although some TIM cells can reach the tissue at early time points following infection, they are not prone to enter the organ. Finally, this early recruitment of Flu-TM was associated with increased levels of IFN-γ in the broncho-alveolar lavage of Flu-TM recipient mice (Fig. 3C). Altogether, these results indicated that the HP1 gene expression signature is associated with the capacity of Flu-TM to be more rapidly recruited to the lung parenchyma to deliver their effector functions and contribute to the protection they can confer against a lethal dose of influenza virus.

### Identification of poised genes associated with memory CD8 T cells

**Figure 4:**
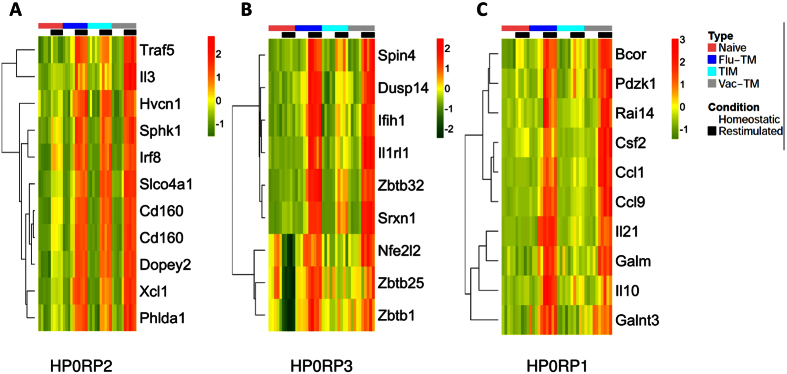
Expression heatmap of the top 10 HP0RP2, HP0RP3 and HP0RP1 genes with a minimal fold change of 2 when comparing Flu-TM to naive cells. Expression profiles in naive cells, Flu-TM, TIM and VV-TM in homeostatic conditions and following restimulation are shown.

We next analysed genes that were differentially expressed following a brief NP68 peptide re-stimulation *in vitro* (R). Genes were again classified in three groups: RP1 (naive = TIM < Flu-TM), RP2 (naive < TIM = Flu-TM) and RP3 (naive < TIM < Flu-TM) (Supplementary Tables S2 and S6–8). RP2 and RP3 segregate naive from memory cells and RP1 segregates Flu-TM from naive and TIM cells (Fig. 2A). These RP-gene-lists contain a number of HP genes that maintain their differential expression following peptide stimulation and poised genes that were not differentially expressed in homeostatic conditions. We focused our analysis on these poised genes as they might reveal new functions associated with memory CD8 T cells efficiency. Hence we analysed genes that were not significantly up regulated by memory cells compared to naive cells in homeostatic conditions (HP0) but showed a significant differential expression in memory CD8 T cells, following peptide stimulation (RP). The expression profile of 10 genes showing the highest fold changes (FC) when comparing naive cells and Flu-TM was validated on VV-TM cells (Fig. 4). The HP0RP2 genes, that show a similar expression profile in Flu-TM and TIM, include two transcriptions factors *Irf8*^28^ and *Traf5*^29^ that control the optimal generation of effector CD8 T cells and two cytokines IL-3 and *Xcl1* that have been previously shown to be poised in memory CD4 or CD8 T cells^30^,^31^. The HP0RP3 highlights new poised genes such as *Il1rl1* that codes for the receptor for the IL-33 alarmin that is produced by non hematopoietic cells during the course of viral infections and that plays an essential role in the development of anti-viral effector CD8 T cells^32^. transcription factors (*Zbtb1*, *Zbtb25*, *Zbtb32*)^33^,^34^,^35^ involved in regulating T cells activation or differentiation or proteins involved in cellular response to stress (*Nfe2l2*) or infection (*Ifih1*). Hence the majority of the RP2 and RP3 poised genes seem to be involved in the generation, regulation or function of memory CD8 T cells.

### The poised gene signature uniquely associated with Flu-TM is enriched in genes coding for chemokines and cytokines

**Figure 5:**
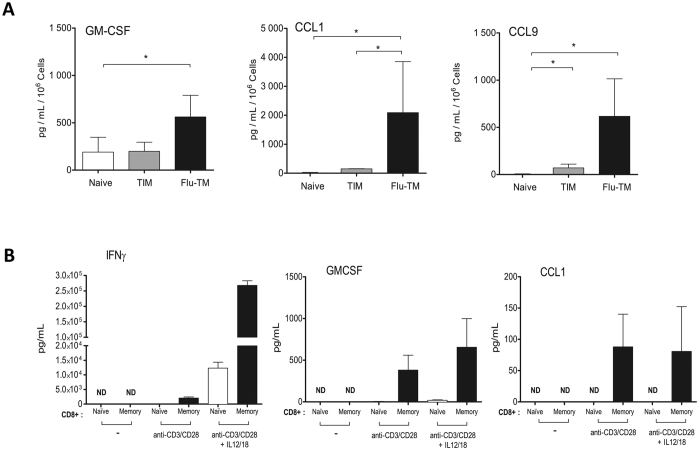
Poised cytokines produced by Flu-TM and human memory CD8 T cells. (**A**) Cytokine profile of stimulated naive, TIM or Flu-TM CD8 T cells. Supernatants were collected after 48 hours of NP68 peptide stimulation for indicated cell subsets. Data are mean ± SD of 3 independent experiments with a pool of four or five mice per group. (**B**) Cytokine production by sorted human naive or memory CD8 T cells. Supernatants were collected after 48 hours of anti-CD3/CD28 stimulation or anti-CD3/CD28 stimulation plus IL-12/IL-18 stimulation. Data are mean ± SD of 5 healthy donors. *p < 0.05 (two tailed unpaired t-test).

The poised genes signature HP0RP1 associated with protective Flu-TM is strongly enriched in cytokines (*Csf2* also known as *Gm-csf*, *Il21* and *Il10*) and CC-chemokines (*Ccl1* and *Ccl9*). Expression of these cytokines by memory CD8 T cells was validated at the mRNA level by PCR (Supplementary Fig. S4). The production of the poised RP1 cytokine GM-CSF and chemokines CCL1 and CCL9 was measured at the protein level following peptide stimulation. Results in Fig. 5A indicate that all three proteins are secreted in significantly larger quantities by Flu-TM compared to TIM or naive F5 cells. Another characteristic of memory CD8 T cells is their ability to increase their functional potential and secrete certain cytokines such as IFN-γ in response to two cytokines, IL-12 and IL-18, associated with inflammatory milieu^36^. Similarly, the production of GM-CSF by Flu-TM, but not CCL1 or CCL9, was strongly induced by IL-12 and IL-18 (Supplementary Fig. S5), indicating that its production by memory CD8 T cells follows the same pattern as IFN-γ. We next looked if the genes coding for these proteins were also behaving as poised genes in human memory CD8 cells. To that aim, we purified human blood memory CD8 T cells and measured their capacity to produce those cytokines/chemokines following stimulation with anti-CD3/CD28, in the presence or absence of IL-12/IL-18. Of note, CCL9 is not conserved in humans, so we focused our analysis on CCL1 and GM-CSF or IFN-γ as a control (Fig. 5B). As described before, GM-CSF or IFN-γ could readily be detected following stimulation^8^. We next could show that memory phenotype but not naive CD8 T cells also produced CCL1 chemokine, indicating that the *CCL1* gene is also poised in human memory CD8 T cells.

## Discussion

Using transcriptome approaches applied to different qualities of CD8 T cells, we have identified unique gene expression signatures that are associated with the capacity of memory cells to protect against a lethal influenza infection. We could show that these immune signatures were conserved, as they were induced by 2 other pathogens (vaccinia virus and *Listeria monocytogenes*) and also based on meta-analysis of memory CD8 T cell datasets obtained using 3 different TCR transgenic CD8 T cells.

We identified a gene expression signature (HP1) associated with protective Flu-TM in homeostatic conditions, i.e. genes expressed by cells in the absence of re-stimulation, that is enriched in genes such as *Ccr5* or *Itga1* and *Itgb1* coding for proteins that control the re-circulation of cells to the lung^24^,^37^,^38^. Indeed, we find that a larger number of protective Flu-TM are recruited to the lung and that the majority of these cells have made their way to the lung parenchyma, in contrast to TIM memory or naive CD8 T cells that can not protect against a lethal dose of virus. Expression of VLA1 by memory CD8 T cells has previously been shown to correlate with their capacity to migrate back to lung^38^, skin^39^ or mucosal tissue^40^. Its expression is also preferentially associated with memory CD8 T cells generated in response to an acute infection compared to chronic infection^14^. Thus VLA1 could be a key marker to identify peripheral memory CD8 T cells that are able to migrate rapidly to tissues such as the lung or skin. Noteworthy, *Itga1* is also one of the few genes that are differentially expressed by TRM^16^. However we cannot exclude that other differentially expressed HP1 gene, coding for proteins such as chemokine receptors regulators (*Rgs16*), actin cytoskeleton regulators (*Anxa1*, *Rhoq*, *Yes1*) are also involved in the control of memory CD8 T cells extravasation and tissue migration. Furthermore, both VLA1 and CCR5 could also contribute to the increased survival of CD8 T cells within the lung^26^,^41^. They might not be the only factors favouring the survival of Flu-TM as the HP1 signature also includes genes that code for proteins that will increase their resistance to environmental stress or to pathogen infection. This property could contribute to their efficiency, as an increased resistance to infection and survival would be key in the context of an infected tissue. Indeed, expression of one of these genes, *Ifitm3*, by TRM protects them from infection by the virus, as its invalidation leads to decreased protection against influenza infection^42^. The HP1 signature does not contain genes coding for classical memory traits such as *Ifng*, as non-protective TIM memory cells also show an increased level of expression of these genes compared to naive CD8 T cells. However, the capacity of Flu-TM to be rapidly recruited to the lung parenchyma targets the IFN-γ to the lung as reflected by the early increase in the IFN-γ content of the BAL. This would mimic, with a little delay, the alarm function of TRM and lead to the recruitment of more peripheral memory cells^5^. Thus the migration capacity of peripheral memory CD8 T cells that allows the rapid delivery of effector functions to the site of infection is a key efficiency determinant of peripheral memory cells. This could represent an essential property, complementing other factors such as the polyfunctionality of memory cells, that one would need to take into account when determining the efficiency potential of these cells. Altogether, the presence in the spleen of memory cells expressing VLA1 and the broader HP1 signature could consequently be used to identify immunisation regimens that lead to the generation of protective memory CD8 T cells. Generation of such cells might depend, among other factors, on the priming route^40^. Moreover, and considering the growing importance of TRM in the protection against infection, it has been shown that, following natural influenza infection, the presence of protective Flu-TM cells in the peripheral lymphoid organs correlates with the generation of efficient TRM in the lung^43^. Thus the presence of protective cells in the periphery could represent a predictive factor for TRM generation, although whether this would hold true in all cases would need further experimental data to be confirmed.

We also defined a set of poised genes that are differentially expressed by Flu-TM compared to TIM memory cells. This signature is highly enriched in genes encoding cytokines and chemokines some of which might be associated with the promotion of T cell responses. Indeed, protective Flu-TM cells produce cytokines that are involved in the recruitment (XCL1), survival and stimulation of antigen cross-presentation (IL-3 and GM-CSF) of dendritic cells (DC)^30^,^44^. IL-3 and XCL1 are poised in both types of memory cells, in contrast to GM-CSF that is only poised in Flu-TM. Expression of GM-CSF and CCL1 is not restricted to mouse CD8 T cells as we could show that their expression was also poised in human memory cells. Similarly to IFN-γ, the production of GM-CSF by memory CD8 T cells could be induced or potentiated by IL-12 and IL-18 in both human and mouse. This could thus represent a positive feedback by which DC-derived IL-12 and IL-18 would synergise with TCR engagement to increase the production of GM-CSF by CD8 T cells, which in turn will promote the cross-presentation capacity of newly recruited immature DC^45^.

Overall we unravel new gene expression signatures that are associated with protective spleen memory CD8 T cells. We highlight the lung migration capacity of these cells as a key property associated with protection. Of interest, these signatures were identified using the pool of memory CD8 cells that are present in lymphoid organs, i.e. that are easily accessible for the monitoring of antigen specific CD8 T cells.

## Methods

### Mice

F5 TCR-transgenic mice were provided by Pr. Dimitris Kioussis^46^ and back-crossed on C57BL/6.Ly5.1 (B6.SLJ-PtprcaPepcb/BoyCrl) background to generate F5xLy5.1 (B6.SJL-PtprcaPepcb/BoyCrl-Tg(CD2-TcraF5,CD2-TcrbF5)1Kio/Jmar). The F5 TCR recognizes the NP68 peptide from influenza A virus (ASNENMDAM) in the context of H2-Db. Mice were bred and housed in specific pathogen–free conditions in the AniRA-PBES animal facility at Lyon, France. All animal experiments were undertaken in full compliance with European regulations and were approved by the local bioethics committee CECCAPP (Comité d’Evaluation Commun au Centre Léon Bérard, à l’Animalerie de transit de l’ENS, au PBES et au laboratoire P4).

### Viruses and immunizations

The recombinant influenza virus strain WSN encoding the NP68 epitope (Flu-NP) was produced by reverse genetics from the A/WSN/33 H1N1 strain^21^. The recombinant vaccinia virus expressing the NP68 epitope (VV-NP) was engineered from vaccinia virus (strain Western Reserve) by Dr. Denise Yu-Lin Teoh in Pr. Sir Andrew McMichael’s laboratory at the MRC (Human Immunology Unit, Institute of Molecular Medicine, Oxford, UK). Influenza or vaccinia virus induced memory cells (Flu-TM or VV-TM) were generated in C57BL/6 J mice (females, 8–12 weeks old) that received 2 × 10^5^ naive F5xLy5.1 CD8 T cells one day before intranasal infection with 2 × 10^5^ TCID_50_ of Flu-NP or 2.10^5^ PFU VV-NP in 20 μL of phosphate-buffered saline. Specific memory CD8 T cells (Flu-TM or VV-TM) were purified at least 6 weeks after immunization. T Inflammatory Memory (TIM) cells were generated as previously described^20^. Briefly, female F5 mice thymectomized at 5–7 weeks of age were injected twice with NP68 peptide (50 nmol, ASNENMDAM, Think Peptides, Oxford, UK) in DPBS. TIM cells were purified at least 6 weeks after immunization.

### Protection and functional assays

To evaluate the degree of protection associated with each CD8 T cell population, 5 × 10^4^ naive, TIM or Flu-TM F5xLy5.1 CD8 T cells were transferred in C57BL/6 J host mice (females, 8–12 weeks old) by i.v. injection in the retro-orbital sinus. Two days later, recipient mice were infected intra-nasally with a lethal dose (1 × 10^7^ TCID50) of Flu-NP68 virus. Mice were observed for illness and weight loss each day, for 10 days after infection. Mice that lost more than 20% of body weight were euthanized. To perform *in vivo* CD8-functional assays 5 × 10^4^ naive, TIM or Flu-TM F5 CD8 T cells were transferred in C57BL/6 J host mice by i.v. injection. Two days later, mice were infected intra-nasally with 2 × 10^5^ TCID_50_ of Flu-NP68 virus.

### Cell preparation and flow cytometric analysis

Intravascular staining was performed to identify lung resident memory CD8 T cells as previously described^27^. Mice were injected with 3 μg of CD45-BV421 antibody (BioLegend) diluted in 300 μl of sterile DPBS (Life Technologies) and sacrificed 3 min after injection. After euthanasia by overdose of pentobarbital, the trachea was exposed and cannulated with a 24-gauge plastic catheter (BD Biosciences). Lungs were lavaged 2 times with 1 ml cold sterile PBS 1X. Cell suspension of broncho-alveolar lavage (BAL) was centrifuged at 4 °C, 300 × g for 10 min. Flow cytometry staining was performed on cell pellets and IFN-γ in cell-free BAL fluid was measured by ELISA.

After lavage, the whole lungs and mediastinal lymph nodes were harvested. Lung tissues were enzymatically digested by using a Lung Dissociation Kit for mouse (Miltenyi Biotec). Spleen and lymph nodes were harvested, mechanically disrupted and filtered through a sterile 100 *μ*m nylon mesh filter. For surface staining, cells were stained for 45 min on ice with the appropriate mixture of monoclonal antibodies and washed with PBS (Life Technologies) supplemented with 1% FCS (Life Technologies) and 0.09% NaN3 (Sigma-Aldrich, Saint Quentin-Fallavier, France). The following antibodies were used: CD45.1 (A20), CD62L (MEL-14), CD8 (53-6.7), CD44 (IM7.8), KLRG1 (2F1), CXCR3 (Cxcr3-173), CD25 (PC61) (all BD PharMingen), Ly6G (1A8), CD11b (M1-70), NK1.1 (PK136), CD4 (RM4-5) (all eBioscience). Intracellular detection of cytokine production was performed after a 4 hours *in vitro* re-stimulation with 10 nM NP68 peptide in the presence of GolgiStop (BD Biosciences). After surface staining, cells were fixed and permealized using CytoFix/CytoPerm (BD Biosciences). The following anti-cytokine mAbs were used: IFN-γ (XMG1.2) and TNF (MP6-XT22), IL-2 (JES6-5H4) (all BD Biosciences), CCL3 (39624, R&D System) and CCL5 (2E9, in-house production). Cell numbers were calculated using fluorescent beads (flow-count fluorospheres, Beckman Coulter). All analyses were performed on a Becton Dickinson FACS LSR II or Fortessa and analysed with FlowJo software (TreeStar, Asland, OR, USA).

The polyfunctionality index was estimated as follow^47^: Polyfunctionality index = Σ^n^_i = 0_*F*_i_*i/n, where *n > 0* is the number of functions studied and F_i_ the frequency of cells performing *i* functions. In this study the polyfunctionality index of specific CD8 T cells was assessed with the data corresponding to the simultaneous detection of 4 cytokines: IFN-γ, IL-2, CCL3 and CCL5.

### Cytokine secretion after *in vitro* stimulation

Sorted Flu-TM, TIM or naive CD8 T cells were cultured (1 × 10^6^ cells/ml) for 24 or 48 hours with NP68 peptide (10 nM) or mouse recombinant IL-12 (10 ng/ml, R&D system) and IL-18 (10 ng/ml, MBL). Supernatants were collected and cytokines production was measured by bead-based multiplexing technology for IFN-γ, TNFα, CCL3, CCL5, IL-3, IL-10, GM-CSF (Bio-Plex Pro, Bio-Rad) or by ELISA for CCL1, CCL9, IL-21, IFN-γ (Mouse DuoSet, R&D system).

### Stimulation of human CD8 T cell

PBMC from peripheral blood of healthy donors (EFS, Lyon) were isolated by Ficoll density gradient (Eurobio). CD8 T cells were isolated by negative selection (Miltenyi Biotec) and labelled with anti-CCR7 (3D12) and anti-CD45RA (T6D11) antibodies. Naive (CD45RA+ CR7+) or memory (CD45RA− CCR7+/−) CD8 T cells were FACS-sorted (over 97% purity). Sorted CD8 T cells (2 × 10^6^ cell/ml) were incubated in presence of IL-2 (5 U/ml, Chiron Novartis), 1 μg/ml of anti-CD3 (UCHT1, BD Biosciences) and 5 μg/ml of anti-CD28 (CD28.2, eBioscience) antibodies or anti-CD3/CD28 antibodies in the presence of human recombinant IL-12 and IL-18 (10 ng/ml, Peprotech). Supernatants were collected 24 or 48 hours later and cytokine production was measured by bead-based multiplexing technology for IFN-γ and GM-CSF (Bioplex Human Cytokine Group I 6-plex Assay, Bio-Rad) or by ELISA for CCL1 (DuoSet human CCL1 kit, R&D system).

### Microarrays

Spleens from eight mice (Flu-TM) or three mice (TIM, naive) were pooled and CD8 T cells were enriched using a MACS CD8a+ T Cell Isolation Kit II for mouse (Miltenyi Biotec). This experimental setting was reproduced five days in a row. Naive, TIM or Flu-TM CD8 T cells were sorted based on CD44 expression levels by FACS-Sorting using a FACS-Aria Instruments (BD Biosciences, Franklin Lakes, NJ, USA) to over 98% purity. Purified CD8 T cells were stimulated with 10 nM NP68 peptide (re-stimulated condition, hereafter R) or not (homeostatic condition, hereafter H) as previously described^48^. Total RNA was extracted using the RNEasy Kit (QIAGEN), and 5–50 ng of total RNA was used for microarray analysis. RNA for the microarray was amplified using the BioArray High Yield RNA Transcript Labeling Kit (ENZO). RNA quality was assessed using the Agilent Model 2100 Bioanalyzer. Samples were hybridized and loaded onto Affymetrix GeneChip Mouse 430 2.0 arrays.

Affymetrix CEL files were processed and analysed with R^49^ and Bioconductor (Release 2.10)^50^ packages. The expression data were computed with the Robust MultiChip Average (RMA) method^51^ implemented in the *affy* package^52^. This pre-processing step included: background correction based on PM probes only, quantile normalization and median polish summarization^51^. The log2 transformed expression data was used for all further analyses.

The differential expression analysis was carried out with the Linear Models for Microarray Analysis method available in the *limma* package^53^. For each comparison the limma p-values were adjusted for multiple testing with the Benjamini-Hochberg method for false discovery rate (FDR)^54^. The FDR adjusted p-values have been used to select the differentially expressed genes (adjusted p-value ≤ 0.05). The following contrasts have been tested: NaR – NaH, TIMR – TIMH, FluR (Flu-TM-R) – FluH (Flu-TM-H), TIMH - NaH, FluH - NaH, FluH – TIMH, TIMR - NaR, FluR - NaR, FluR – TIMR. From all the lists of differentially expressed probe sets we eliminated those without gene annotation. Two FluH samples showed a degree of activation in the PCA compared to the three other FluH samples. To only identify genes expressed in homeostatic conditions, we eliminated the probe sets that are differentially expressed (adjusted p-value ≤0.1) between the two FluH samples that show a degree of activation in the PCA compared to the three other FluH samples from all the lists corresponding to comparisons that implied the FluH condition, 362 such probe sets have been excluded (Supplementary Table S1). For PCA analysis non-informative probe sets were filtered out according to the informative/non-informative (I/NI) calls implemented in the FARMS Bioconductor package^55^,^56^.

Functional enrichment was performed using the Model-based Gene Set Analysis (MGSA)^57^ Bioconductor package in R. Gene categories were sourced from the Biological Process tree of the Gene Ontology.

### Discretized fold change profiles

A fold change profile is defined for each probe set as a vector composed of (FC_1_ = TIMH - NaH, FC_2_ = FluH - NaH, FC_3_ = FluH – TIMH) in the homeostatic condition and (FC_1_ = TIMR - NaR, FC_2_ = FluR - NaR, FC_3_ = FluR – TIMR) in the re-stimulated condition. The discretized FC profiles have been obtained from the FC profiles using the *signum* function as follows:


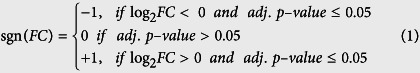


Among the possible discretized FC profiles we selected only those corresponding to compatible order relations, e.g. the (−1, 0, 0) profile implies the following relations:


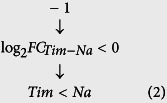



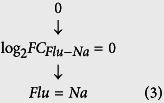



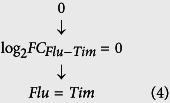


The Eqs 2 and 3 relations imply Flu > TIM while in Eq. 4 we have Flu = TIM. This type of profiles was eliminated from our study. The remaining discretized FC profiles were annotated in six distinct classes, from P1 to P6.

### Meta-analysis

To identify a homeostatic memory signature we performed a meta-analysis with 8 datasets comparing memory CD8 T cells to naive CD8 T cells (Supplementary Table S9). All the datasets have been pre-processed as described above (RMA pre-processing and LIMMA analysis). We used a rank product-based meta-analysis method, available in the *RankProd* package^58^. Rankprod method uses as input complete pre-processed expression data matrix from each experiment. All possible pair comparisons, treatment vs. control, were performed within each experiment. Suppose one has in the first experiment t treatment samples and c control samples, then t x c fold changes will be computed for each gene. Fold changes are transformed in ranks for each comparison, the largest fold change having rank = 1. After pair comparisons within each experiment, the rank geometric mean is computed for each gene. A permutation analysis was used to compute the p- and q-values. 500 permutations have been employed in order to estimate the reference distribution of rank products and to assess statistical significance and control false discovery rate (FDR ≤ 0.01).

### Pathway analysis

The functional analysis of gene expression data has been performed with SPIA method and R/Bioconductor package (Signalling Pathway Impact Analysis). In addition to performing classical enrichment analysis the SPIA method allows us to take into account the pathway topology and the amount of gene perturbation (FC). The KEGG information used in our analysis is the one included in the R package, and has been downloaded from the KEGG’s website on 09.07.2012. The SPIA analysis output of an expression dataset includes for each pathway the NDE (number of genes differentially expressed for a given pathway) and its associated p-value (P_NDE_), a perturbation index (tA) and its associated p-value (P_PERT_). The two p-values are combined into one global probability value (P_G_) that we have used for ranking and filtering the pathways.

### Taqman Low Density Array (TLDA)

Microarray data was validated using the Applied Biosystem TaqMan Low Density Array (TLDA) format 384, which allows the analysis of 47 gene targets in single reactions and of one mandatory endogenous control gene (HPRT). cDNA was generated using iScript cDNA Synthesis Kit (Bio-Rad) according to the manufacturer’s instructions. The cDNA obtained was amplified using a pre-designed TLDA plate (Applied Biosystems) with user-defined probes. The RT-qPCR was run on the 7900 HT Applied Biosystem machine. Raw data were analyzed with the RQ Manager software. Expression values were normalized to HPRT (housekeeping gene) mRNA. Relative expression of each gene was calculated by the (delta-delta) Ct method.

## Additional Information

**Accession codes:** The microarray data presented in this article have been deposited to the Gene Expression Omnibus (http://www.ncbi.nlm.nih.gov/geo/) under accession numbers: GSE70763 and GSE86601.

**How to cite this article**: Brinza, L. *et al*. Immune signatures of protective spleen memory CD8 T cells. *Sci. Rep*. **6**, 37651; doi: 10.1038/srep37651 (2016).

**Publisher’s note:** Springer Nature remains neutral with regard to jurisdictional claims in published maps and institutional affiliations.

## Supplementary Material

Supplementary Information

## Figures and Tables

**Table 1:** Characteristic of HP2 and HP3 gene lists that discriminate between naive and memory CD8 T cells.

HP2	84% in meta-analysis, p-val < 2.2e-16
Top 10 increased FC Flu-TM compared to naive cells	IFNg, Asns, Nrp1, Kcnk6, Slamf7, Ctla4, Gm20265, Ms4a4c, Vmp1, Sidt1
Top 10 decreased FC Flu-TM compared to naive cells	Sox4, Tubb2b, Ikzf2, Basp1, Ldhb, Cd24a, Rab4a, Acsl3, Baz2b, Trat1
GO Terms	GO:0000165 MAPK cascade
	GO:0006897 endocytosis
	GO:0030217 T cell differentiation
**HP3**	**92% in meta-analysis, p-val < 2.2e-16**
Top 10 increased FC Flu-TM compared to naive cells	Klrc1, Ccl5, Cd44, Cxcr3, S100a6, Fasl, Klrc2, Gzmk, Klrk1, Runx2
Top 10 decreased FC Flu-TM compared to naive cells	Ccr9,Cnn3, Atp1b1, Dntt,Bambi-ps1m Lef1,Igf1r, Ifngr2, AI131651, BB163080
GO Terms	GO:0051707 response to other organism
	GO:0032101 regulation of response to external stimulus
	GO:0046649 lymphocyte activation
	GO:0071345 cellular response to cytokine stimulus

The percentage and Fisher test p-value of HP2 or HP3 genes found in the meta-analysis (Supplementary Tables S9–11) is given. 2.2e-16 order p-values indicate significant enrichment of meta-analysis genes in HP2 and HP3 lists. Moreover, the FC are coherent i.e. Hp2 and Hp3 genes are exclusively found in the metaUP list (with one exception for p3 Plac8 gene) while Hp2m, Hp3m genes are exclusively found in the metaDOWN list. 10 genes with the highest increased or decreased fold changes are shown. The GO terms are the top-ranking terms from the Gene Ontology “Biological Process” tree returned by a Bayesian network-based gene-set analysis (MGSA) applied to the respective gene lists.
