# Supplementary material for: Immune signatures of protective spleen memory CD8 T cells
Source: Sci Rep. 2016 Nov 24;6:37651. doi: 10.1038/srep37651 (PMC5121635; doi:10.1038/srep37651)
Supplement: Supplementary Information [file srep37651-s1.pdf]

Immune signatures of protective spleen memory CD8 T cells

Lilia Brinza, Sophia Djebali, Martine Tomkowiak, Julien Mafille, Céline Loiseau, Pierre-Emmanuel Jouve, Simon de Bernard, Laurent Buffat, Bruno Lina, Michèle Ottmann, Manuel Rosa-Calatrava, Stéphane Schicklin, Nathalie Bonnefoy, Grégoire Lauvau, Morgan Grau, Mélanie Wencker, Christophe Arpin, Thierry Walzer, Yann Leverrier and Jacqueline Marvel

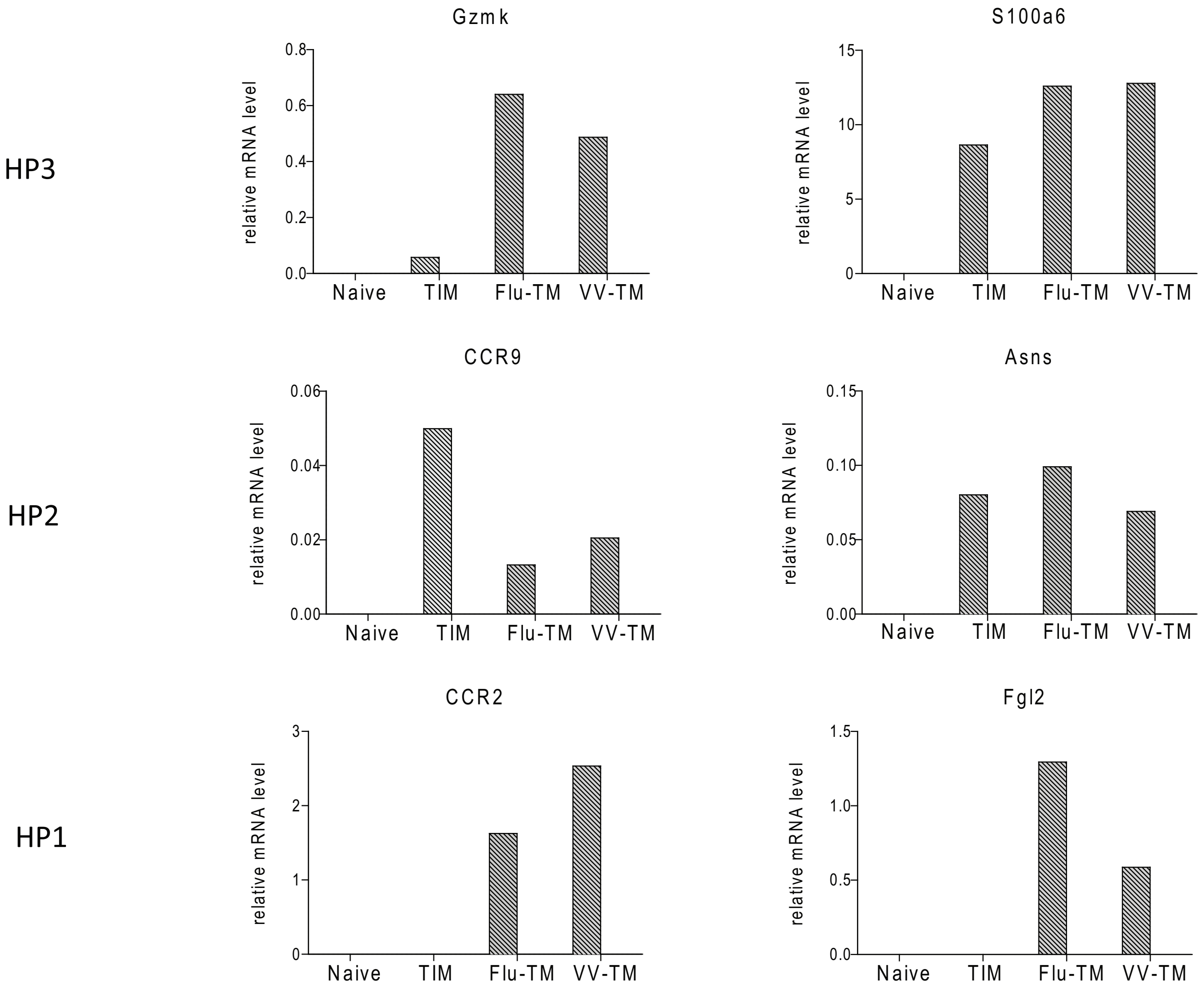

Supplementary Fig. S1. Validation of transcriptomic signature by quantitative RT-PCR assay. mRNA of Naive, TIM or Flu-TM CD8 T cells were analyzed *ex vivo* (homeostatic condition - dashed bar). The expression of each selected gene was measured in triplicate with TLDA technology. Data show one out of 2 independent experiments.

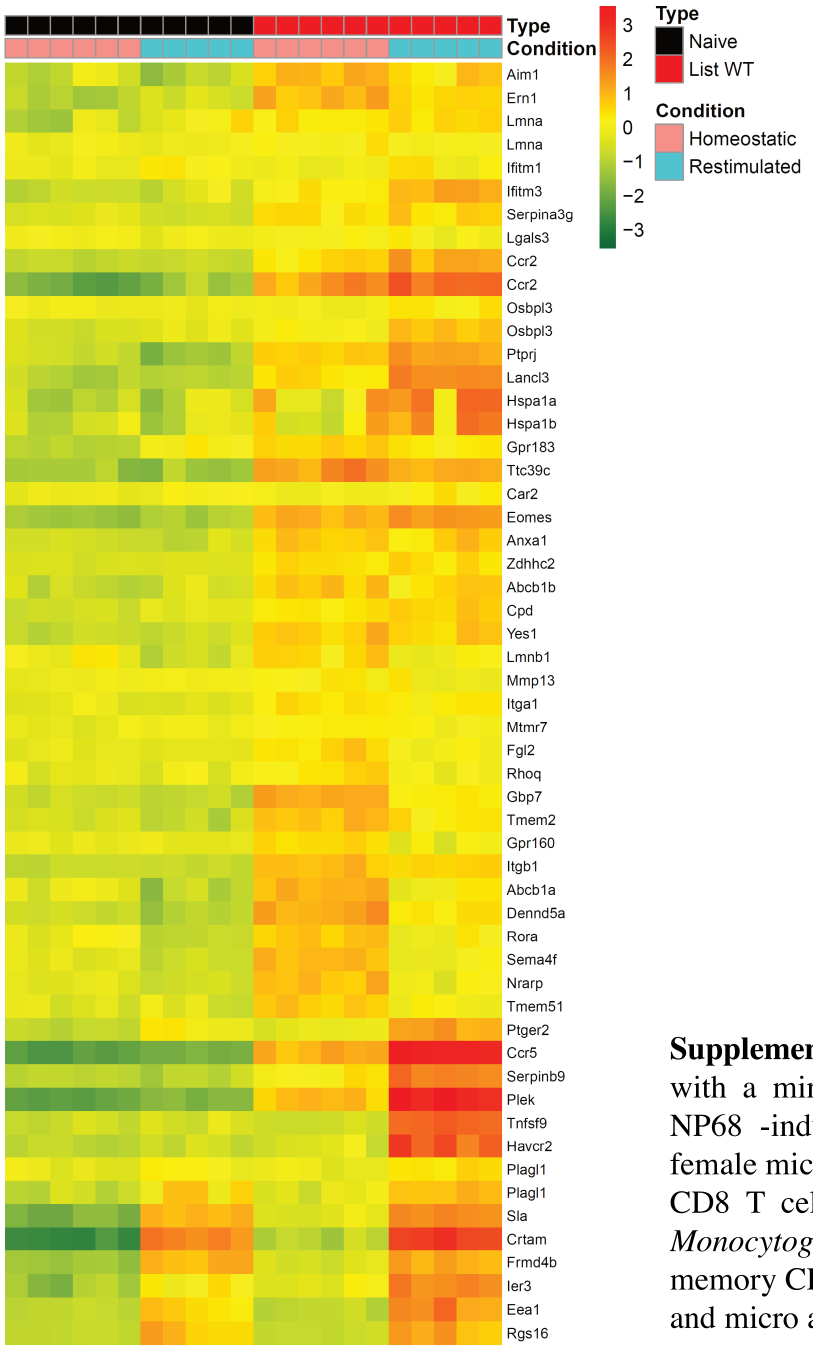

Supplementary Fig. S2. Expression heatmap of the top 55 HP1 genes with a minimal FC of 4 when comparing *Listeria Monocytogenes*-NP68 -induced memory cells (List WT) to naive cells. C57BL/6J female mice (8-12 weeks old) that had received 2×10^5 naive F5xLy5.1 CD8 T cells one day before were infected *i.v.* with $3x10^3$ *Listeria Monocytogenes* strain 10403S expressing NP68 epitope. Specific memory CD8 T cells were purified at least 6 weeks after immunization and micro arrays data were generated as described in the methods

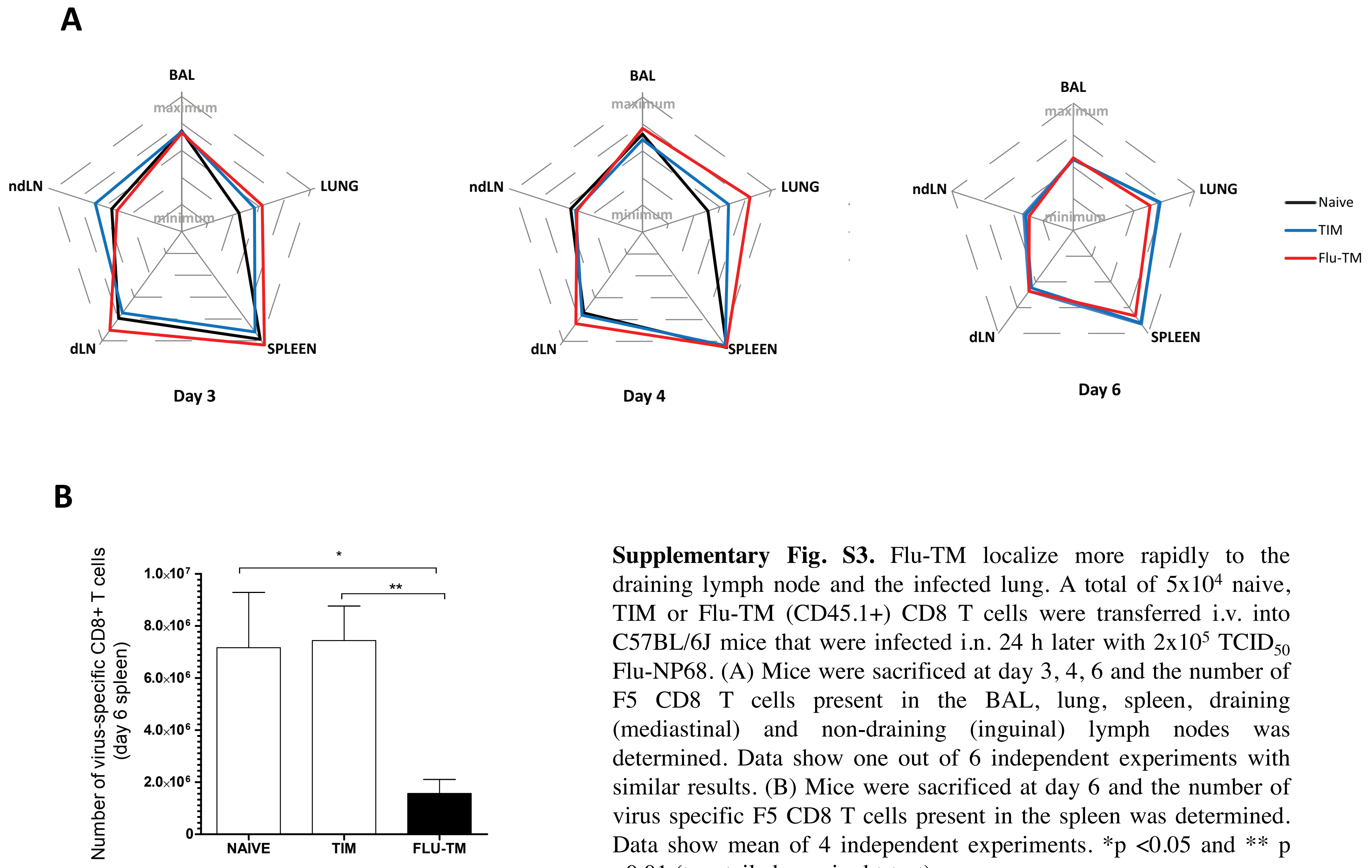

Supplementary Fig. S3. Flu-TM localize more rapidly to the draining lymph node and the infected lung. A total of $5x10^4$ naive, TIM or Flu-TM (CD45.1+) CD8 T cells were transferred i.v. into C57BL/6J mice that were infected i.n. 24 h later with $2x10^5$ $TCID_{50}$ Flu-NP68. (A) Mice were sacrificed at day 3, 4, 6 and the number of F5 CD8 T cells present in the BAL, lung, spleen, draining (mediastinal) and non-draining (inguinal) lymph nodes was determined. Data show one out of 6 independent experiments with similar results. (B) Mice were sacrificed at day 6 and the number of virus specific F5 CD8 T cells present in the spleen was determined. Data show mean of 4 independent experiments. *p <0.05 and ** p <0.01 (two tailed unpaired t-test)

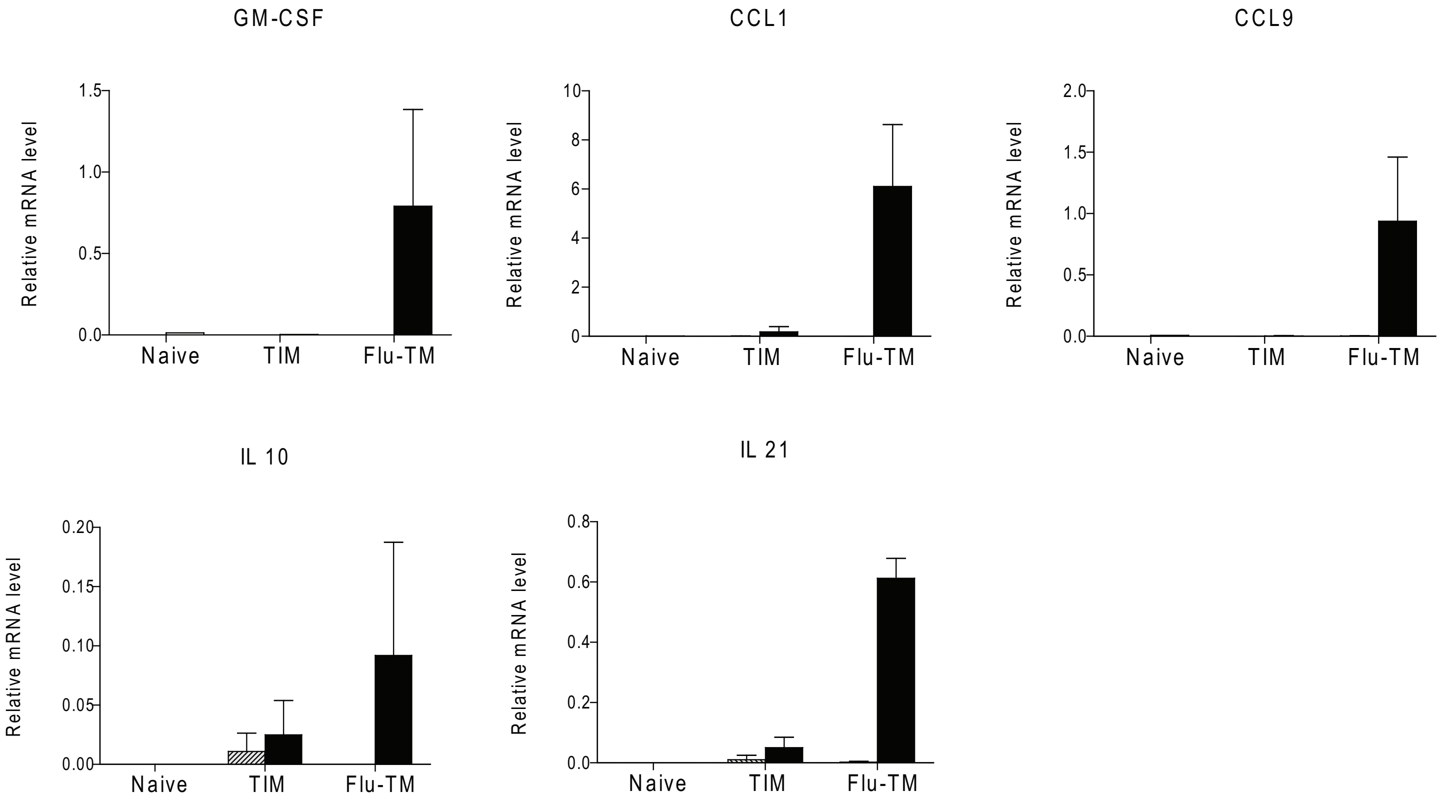

Supplementary Fig. S4. Validation of transcriptomic signature by quantitative RT-PCR assay. mRNA of Naive, TIM or Flu-TM CD8 T cells were analyzed *ex vivo* (dashed bar) or after 2h of NP68 peptide stimulation (black bar). The expression of each selected gene was measured in triplicate. Data show mean of 2 independent experiments.

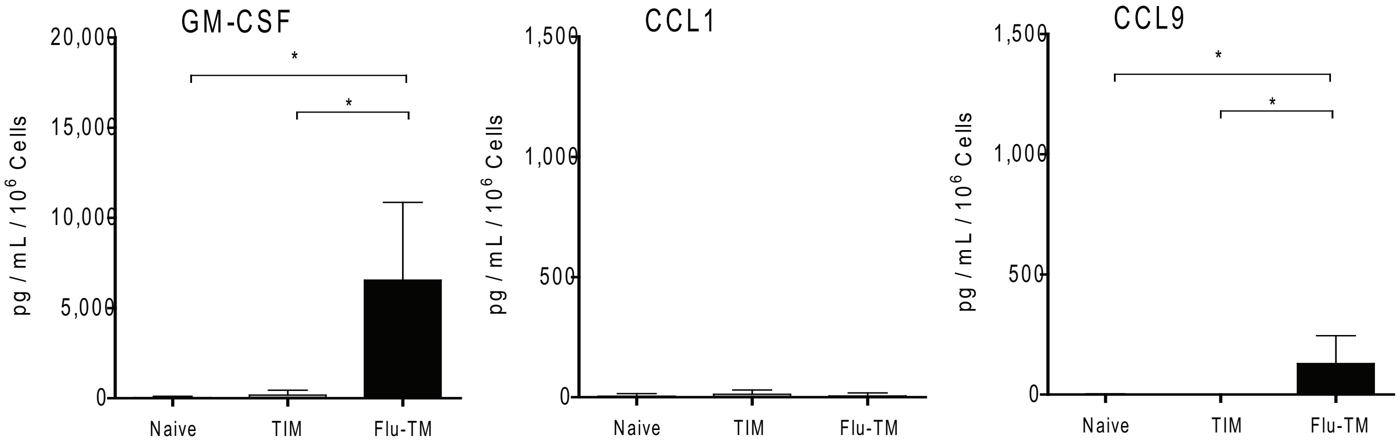

Supplementary Fig. S5. IL-12 and IL-18 induce the production of GM-CSF by Flu-TM memory cells. Cytokine profiles of naive, TIM and Flu-memory CD8 T cells. Supernatants were collected 48h after IL-12/IL-18 stimulation. Cytokine production was measured by bead-based multiplexing technology for GM-CSF or by ELISA for CCL1, CCL9. Data are mean ± SD of 3 independent experiments with a pool of four or five mice per group. *p <0.05 (two tailed unpaired t-test).

SUPPLEMENTARY TABLE S1. Number of differentially expressed probe sets and genes. The FDR adjusted p-values have been used to select the differentially expressed genes/probe sets (adjusted p-value ≤ 0.05) for each comparison (see Methods for further information). A: Number of differentially expressed probe sets in the lists. B: Number of differentially expressed probe sets after FluH correction (see methods). C: Number of differentially expressed probe sets corresponding to a gene, D. Number of unique genes in the lists.

List name	A	B	C	D
TIMH - NaH	1823	1823	1618	1174
FluH - NaH	4690	4574	4028	2821
FluH - TIMH	2399	2302	2073	1526
TIMR - NaR	1532	1532	1370	985
FluR - NaR	4451	4451	3999	2847
FluR - TIMR	1265	1265	1148	819
NaR - NaH	11789	11789	10485	6636
TIMR - TIMH	9408	9408	8386	5383
FluR - FluH	8533	8223	7392	4921

SUPPLEMENTARY TABLE S2. Analysis of discretized fold change profiles - summary. For each differentially expressed probe set, in at least one comparison a discretized fold change was defined for three comparisons (TIM – Na, Flu – Na, Flu – TIM), in homeostatic (H) and re- stimulated (R) conditions. Each FC was discretized as follows: 1 if $\log_2$ FC>0 and the adjusted p- value <0.05, -1 if $\log_2$ FC<0 and the adjusted p-value <0.05 or 0 otherwise. Probe sets with a compatible order relation were selected (P1 to P6). (a) Probe set number belonging to this profile, (b) corresponding gene number belonging to this profile, (c) supplementary table number containing full gene list.

Tim/Na	Flu/Na	Flu/Tim	Order relation	Profile	Homeostatic			Restimulation		
					(a)	(b)	(c)	(a)	(b)	(c)
0	1	1	Na=Tim<Flu	P1	305	234	3 - up	232	184	6 - up
0	-1	-1	Flu<Na=Tim	P1m	70	42	3 - down	38	29	6 - down
1	1	0	Na<Tim=Flu	P2	119	96	4 - up	221	167	7 - up
-1	-1	0	Tim=Flu<Na	P2m	146	104	4 - down	133	94	7 - down
1	1	1	Na<Tim<Flu	P3	202	157	5 - up	203	141	8 - up
-1	-1	-1	Flu<Tim<Na	P3m	64	43	5 - down	37	27	8 - down
1	0	-1	Na=Flu<Tim	P4	4	3	NS	2	2	NS
-1	0	1	Tim<Na=Flu	P4m	2	2	NS	4	2	NS
-1	1	1	Tim<Na<Flu	P5	4	2	NS	1	1	NS
1	1	-1	Na<Flu<Tim	P6	1	1	NS	3	2	NS
-1	-1	1	Tim<Flu<Na	P6m	2	2	NS	2	2	NS

SUPPLEMENTARY TABLE S3. Genes with a fold change of at least 2 in the FLU-TM to Naive comparison and belonging to one of the six following profiles HP1 are shown. FC for the TIM to naive comparison are given. If the gene was found in the meta-analysis (tables S9, 10 and 11) its rank is given. For certain genes multiple probe sets are present on the Affymetrix microarrays, the profile for each probe set is given.

HP1- Upregulated genes

Gene	FluH-NaH	TimH-NaH	RankMeta	Classe profils
Plek	5.1	0	6	p3, p1, p1
Fgl2	4.7	0	121	p1
Ifitm1	3.6	0	382	p1
Rgs16	3.5	0	0	p1
6230424C14Rik	3.5	0	0	p1
Hspa1a	3.5	0	230	p1
Gpr15	3.2	0	368	p1
Pdcd1	3.2	0	516	p1
Ccr5	3.2	0	7	p3, p1, p1
Hspa1b	3.1	0	0	p1
Yes1	3.1	0	60	p1
Ttc39c	3.1	0	30	p0, p1, p1
Anxa1	3	0	26	p1
Osbpl3	3	0	48	p1
Itga1	3	0	62	p1
Il2	2.9	0	0	p1
Lgals3	2.8	0	243	p1
Eomes	2.8	0	42	p3, p1
Ifitm3	2.7	0	275	p1
Ptger2	2.7	0	389	p1
Serpina3g	2.6	0	0	p1
Crtam	2.6	0	87	p1
AA467197	2.6	0	123	p1
Serpinb9	2.5	0	114	p1
Ier3	2.5	0	129	p1
Itgb1	2.5	0	34	p3, p3, p1
Cpd	2.5	0	186	p3, p3, p3, p0, p1, p1, p1
Gpr160	2.4	0	165	p1
Abcb1b	2.4	0	354	p1
Lmnb1	2.4	0	433	p1
Lancl3	2.3	0	122	p1
Gbp7	2.3	0	260	p1
Tmem2	2.3	0	141	p3, p1, p1
Frmd4b	2.3	0	333	p3, p1, p1
Plagl1	2.3	0	562	p1, p0
2610027H17Rik	2.2	0	0	p1
Car2	2.2	0	0	p1
Ahr	2.2	0	0	p1
Tnfsf9	2.2	0	0	p1
Ccr2	2.2	0	22	p1
Ptprj	2.2	0	44	p1
Dennd5a	2.2	0	95	p1
Rora	2.2	0	103	p1
Gpr183	2.2	0	111	p1
Mtmr7	2.2	0	306	p1
Lmna	2.2	0	307	p1
Abcb1a	2.2	0	139	p3, p1
Smox	2.1	0	0	p1
Tmem51	2.1	0	220	p1
Eea1	2.1	0	286	p1
Sema4f	2.1	0	292	p1
Mmp13	2	0	0	p1
Zdhhc2	2	0	55	p1
Nrarp	2	0	84	p1
Ern1	2	0	130	p1
Aim1	2	0	134	p1
Sla	2	0	268	p1
Rhoq	2	0	364	p1
Havcr2	2	0	514	p1

Itpripl2	1.9	0	106	p1
Vim	1.9	0	193	p1
Rnf157	1.9	0	246	p1
Reck	1.9	0	341	p1
Ret	1.9	0	416	p1
Dusp4	1.8	0	0	p1
Sytl2	1.8	0	81	p1
Il10ra	1.8	0	98	p1
Baiap3	1.8	0	156	p1
Cish	1.8	0	159	p1
Emp1	1.8	0	214	p1
Dtx4	1.8	0	235	p1
Mettl21d	1.8	0	439	p1
Alcam	1.8	0	161	p3, p1, p1, p1, p1
3110043O21Rik	1.7	0	0	p1
Smpdl3b	1.7	0	46	p1
5830416P10Rik	1.7	0	97	p1
Slc25a24	1.7	0	102	p1
Socs2	1.7	0	323	p1
Cxcr4	1.7	0	415	p1
Itgav	1.7	0	0	p1, p1, p0
Car5b	1.7	0	67	p3, p1
BC016495	1.7	0	278	p0, p3, p1
2310031A07Rik	1.6	0	0	p1
Sv2c	1.6	0	0	p1
Fcgr2b	1.6	0	41	p1
1700025G04Rik	1.6	0	66	p1
Sytl3	1.6	0	177	p1
Cldnd1	1.6	0	184	p1
Cd80	1.6	0	370	p1
Ifitm2	1.6	0	702	p1
Vps54	1.6	0	427	p3, p1, p0, p1
Fosl2	1.5	0	0	p1
A930041H05Rik	1.5	0	0	p1
Vmn2r84	1.5	0	0	p1
B4galnt4	1.5	0	0	p1
Raph1	1.5	0	148	p1
Map3k8	1.5	0	229	p1
Tmbim1	1.5	0	239	p1
Gprin3	1.5	0	272	p1
Rab19	1.5	0	317	p1
Rbpj	1.5	0	474	p1
Nek6	1.5	0	511	p1
Slc3a2	1.5	0	563	p1
Zeb2	1.5	0	53	p0, p1, p1, p0
Endod1	1.5	0	71	p3, p3, p3, p1
Nfatc2	1.5	0	173	p3, p1
Casp3	1.5	0	273	p3, p1
Adam9	1.4	0	0	p1
Esm1	1.4	0	0	p1
Gzmm	1.4	0	63	p1
Entpd1	1.4	0	70	p1
Adam8	1.4	0	305	p1
Ptger4	1.4	0	334	p1
Cd7	1.4	0	340	p1
Med7	1.4	0	470	p1
Jak2	1.4	0	475	p1
Marveld2	1.4	0	688	p1
Gla	1.4	0	704	p1
Bcl2l1	1.4	0	127	p0, p1, p1, p0
Rgs3	1.4	0	174	p3, p3, p1
Abi2	1.4	0	270	p3, p1
Tnfrsf9	1.4	0	403	p0, p1
Ctnna1	1.3	0	0	p1
Kif23	1.3	0	0	p1
Zik1	1.3	0	0	p1
LOC73899	1.3	0	0	p1
Perp	1.3	0	0	p1

Mettl7a1	1.3	0	231	p1
Lpin2	1.3	0	282	p1
Acpl2	1.3	0	304	p1
Lcn4	1.3	0	329	p1
Klf10	1.3	0	465	p1
Nudt4	1.3	0	534	p1
Mki67	1.3	0	535	p1
Txnrd1	1.3	0	589	p1
Fryl	1.3	0	632	p1
Ubash3b	1.3	0	637	p1
Id2	1.3	0	19	p3, p3, p1
Ccdc50	1.3	0	91	p3, p3, p2, p1
Pvt1	1.3	0	256	p2, p1, p1
Slco3a1	1.3	0	348	p0, p1, p1
Cd47	1.3	0	445	p3, p0, p1, p1
Dynlt3	1.3	0	487	p2, p3, p1
Serpini1	1.2	0	0	p1
Rpap3	1.2	0	0	p1
Sult2b1	1.2	0	0	p1
Gpr65	1.2	0	0	p1
Ccrn4l	1.2	0	0	p1
5930433N17Rik	1.2	0	0	p1
A630035D09Rik	1.2	0	0	p1
Got1	1.2	0	0	p1
Elk3	1.2	0	0	p1
Tanc2	1.2	0	0	p1
Cdc42ep3	1.2	0	150	p1
Dennd4a	1.2	0	175	p1
Myadm	1.2	0	238	p1
Gfod1	1.2	0	288	p1
Mthfs	1.2	0	293	p1
Errfi1	1.2	0	330	p1
Vopp1	1.2	0	358	p1
Slc15a3	1.2	0	482	p1
Map2k3	1.2	0	529	p1
Rhof	1.2	0	633	p1
Gem	1.2	0	654	p1
Glipr1	1.2	0	697	p1
Pygl	1.2	0	699	p1
Tiparp	1.2	0	0	p0, p1, p1
Ccnd2	1.2	0	0	p0, p0, p1
Cobll1	1.2	0	43	p3, p1
Ms4a6d	1.2	0	117	p3, p1
Hip1	1.2	0	151	p0, p1
Itga4	1.2	0	154	p2, p0, p0, p0, p1
Smad3	1.2	0	166	p3, p1
Litaf	1.2	0	391	p3, p1
Plxnc1	1.2	0	413	p2, p0, p1
St6galnac4	1.2	0	435	p3, p1
Cpox	1.2	0	472	p2, p1
Slc37a3	1.1	0	0	p1
Iars	1.1	0	0	p1
Kbtbd7	1.1	0	0	p1
Ehd4	1.1	0	0	p1
Klf4	1.1	0	0	p1
Baz1a	1.1	0	0	p1
Ptgfrn	1.1	0	0	p1
Luzp1	1.1	0	0	p1
Dtl	1.1	0	0	p1
Aim2	1.1	0	0	p1
Gabarapl1	1.1	0	0	p1
2310079F09Rik	1.1	0	0	p1
Chst12	1.1	0	0	p1
Arhgap21	1.1	0	0	p1
Maf	1.1	0	0	p1
Gata3	1.1	0	99	p1
Pik3ap1	1.1	0	113	p1
Cysltr2	1.1	0	233	p1

Slc4a7	1.1	0	258	p1
Plekhg3	1.1	0	279	p1
Ptpn3	1.1	0	285	p1
Tmem140	1.1	0	429	p1
Sypl	1.1	0	449	p1
Tnfrsf18	1.1	0	477	p1
Mapk12	1.1	0	582	p1
Samsn1	1.1	0	600	p1
D8Ertd82e	1.1	0	639	p1
D16Ertd472e	1.1	0	710	p1
Nfkbiz	1.1	0	-580	p1, p0
Abhd5	1.1	0	0	p0, p1
Hs3st3b1	1.1	0	144	p3, p1
Gramd1b	1.1	0	172	p3, p1
Atp10a	1.1	0	347	p0, p0, p1
Tnfaip3	1.1	0	607	p0, p1
Mad2l1	1	0	0	p1
Tmed5	1	0	0	p1
Pawr	1	0	0	p1
Parp12	1	0	0	p1
Cd72	1	0	0	p1
Slc5a3	1	0	0	p1
Cdc14a	1	0	0	p1
3830612M24	1	0	0	p1
Homer1	1	0	0	p1
Slc7a1	1	0	0	p1
Mapre2	1	0	108	p1
Tmem154	1	0	132	p1
Zcchc18	1	0	222	p1
Stat4	1	0	302	p1
Il15	1	0	312	p1
Wee1	1	0	402	p1
Parp8	1	0	522	p1
Cpeb2	1	0	574	p1
9630033F20Rik	1	0	605	p1
Sec24d	1	0	646	p1
Hps4	1	0	687	p1
2410127L17Rik	1	0	0	p0, p1
Stx11	1	0	227	p3, p1

HP1 - Downregulated genes

Gene	FluH-NaH	TimH-NaH	RankMeta	Classe profils
Slc6a19	-2.9	0	-15	p1m
Ift80	-2.6	0	-10	p1m
Tmie	-2.5	0	-78	p1m
Klf7	-2.4	0	-185	p3m, p1m, p1m, p1m
Als2cl	-2.2	0	-63	p1m
Klf3	-2.2	0	-236	p3m, p1m, p1m, p1m, p1m, p1m
Klf2	-2.1	0	-311	p1m
Ccr7	-2	0	-39	p1m
A930005H10Rik	-1.9	0	-196	p1m
Actn1	-1.8	0	-14	p1m
D530037H12Rik	-1.8	0	0	p1m
Smc4	-1.7	0	-45	p1m
Fam101b	-1.6	0	-81	p1m
Myb	-1.5	0	-176	p1m
Prkd2	-1.5	0	-100	p1m
Cyp2d22	-1.5	0	0	p1m
Cd55	-1.5	0	-77	p1m, p1m, p0
Hspb11	-1.3	0	-169	p1m
Ccnd3	-1.3	0	0	p1m
Satb1	-1.3	0	-147	p2m, p2m, p2m, p2m, p1m
Slc43a2	-1.3	0	-55	p3m, p1m, p1m
Pgpep1l	-1.2	0	-509	p1m
Ugcg	-1.2	0	-310	p1m
Il6ra	-1.2	0	-50	p1m
4930520K10Rik	-1.2	0	0	p1m
Adk	-1.2	0	-58	p2m, p3m, p1m, p1m

Trib2	-1.2	0	-28	p1m, p0
Il6st	-1.2	0	-19	p2m, p2m, p1m, p1m
Emp3	-1.1	0	-773	p1m
Crlf3	-1.1	0	-448	p1m
Thada	-1.1	0	-327	p1m
Ypel2	-1.1	0	-290	p1m
Tmc6	-1.1	0	-271	p1m
Stk38	-1.1	0	-235	p1m
Lipa	-1.1	0	-75	p1m
Pdk1	-1.1	0	-53	p1m
9430025N12Rik	-1.1	0	0	p1m
Rasgrp2	-1.1	0	-336	p2m, p1m, p1m
Mettl9	-1	0	-316	p1m
Cyb5	-1	0	-162	p1m
Qser1	-1	0	-87	p1m
N4bp2	-1	0	-82	p1m

SUPPLEMENTARY TABLE S4. Genes with a fold change of at least 2 in the FLU-TM to Naive comparison and belonging to one of the six following profiles HP2 are shown. FC for the TIM to naive comparison are given. If the gene was found in the meta-analysis (tables S9, 10 and 11) its rank is given. For certain genes multiple probe sets are present on the Affymetrix microarrays, the profile for each probe set is given.

HP2 - Upregulated genes

Gene	FluH-NaH	TimH-NaH	RankMeta	Classe profils
AW555464	1	0,6	0	p2
Socs5	1	0.7	0	p2
Myo1d	1.1	0.8	0	p2
Lman1	1.1	0.8	0	p2
Cd52	1	0.8	0	p2
Gm10345	1.1	0.8	0	p2
Dus4l	1.3	0.8	0	p2
Dyx1c1	1	0.9	0	p2
Ttf2	1.1	0.9	0	p2
Ube2t	1.1	0.9	0	p2
Cdca5	1.7	1	0	p2
Ezh2	1.6	1	0	p2
Rab20	1.1	1.1	0	p2
LOC641050	1.3	1.1	0	p2
Gsg2	1.6	1.2	0	p2
Gm13152	1.9	1.4	0	p2
Gm20265	2.8	3.2	0	p2
Ifng	7.6	6.3	5	p2
Slamf7	3.2	2.3	25	p2
Il2ra	2.3	1.4	54	p2
Ttc39b	1.9	1.7	65	p2
Ppm1j	1.7	1.3	74	p2
Il12rb2	1.2	1.2	75	p2
Asns	4.1	3.7	79	p2
Ly6a	2.3	2.5	88	p2
Pogk	1.2	0.9	100	p2
Pion	1	0.7	104	p2
N4bp1	1.4	0.9	115	p2
Kcnk6	3.3	2.7	116	p2
Fgf13	1.6	1.2	120	p2
Efhd2	1.8	1.4	124	p2
Tspan31	1.2	1.1	131	p2
Gab3	1	0.8	136	p2
Ms4a4c	2.7	2.8	138	p2
Prkar2a	1.2	0.7	153	p2
Rnf138	2.4	1.9	158	p2
Gimap7	1.6	1.8	163	p2
Dennd4c	1.5	1	178	p2
Ggh	1.4	0.8	181	p2
Pla2g16	1.8	1.7	192	p2
AW112010	1.6	1.2	195	p2
Sidt1	2.4	2.5	203	p2
Prr13	1.1	0.9	204	p2
Ctsw	1.6	1.3	215	p2
Ctla4	3	3.4	217	p2
Vmp1	2.4	2.2	236	p2
Scpep1	1.3	1.1	267	p2
Mapk11	1	0.8	277	p2
Golm1	1.6	1.1	281	p2
Lpxn	1.3	0.9	298	p2
Atxn1	1.6	1.2	316	p2

Oplah	1.1	0.8	331	p2
Ncf4	1.6	1.1	335	p2
3830406C13Rik	1.5	1	349	p2
Ksr1	1	0.6	351	p2
Cyfip1	1.3	0.8	355	p2
Man2a1	1.1	0.7	366	p2
Gsto1	1.6	1.2	374	p2
Bag3	1.2	0.8	376	p2
Pmaip1	1.2	0.9	387	p2
Clic4	2.2	1.6	395	p2
Sfi1	2.2	2.3	401	p2
2610029I01Rik	1.5	1.3	404	p2
Ap1s2	1	0.7	406	p2
Ank	1.1	0.9	411	p2
Atp11a	1.6	1.3	417	p2
Kbtbd11	1.1	0.8	466	p2
Trim30d	1.3	1.2	492	p2
Fam92a	1.3	1.1	530	p2
Tbc1d12	1.5	0.8	532	p2
Chst2	1.2	0.9	597	p2
Dhrs7	1.1	0.7	628	p2
Mns1	1.1	0.8	638	p2
Slc12a2	1.1	0.8	0	p2. p0. p0
Tpi1	1.1	0.9	0	p0. p2
Lpgat1	1	0.9	0	p2. p0
Nrp1	3.4	2.6	50	p3. p2. p3. p2
Adam19	2	1.5	80	p2. p3
Ccdc50	2.2	1.6	91	p3. p3. p2. p1
Dock9	1.6	0.9	137	p3. p2. p0
Itga4	1.1	0.7	154	p2. p0. p0. p0. p1
Arsb	1.1	0.8	164	p2. p0
Chpt1	1.9	1.2	188	p2. p0
Rhoc	1.1	0.7	191	p0. p2
Dnaja4	2.3	1.7	232	p3. p3. p2
Pvt1	1.1	1.1	256	p2. p1. p1
Icos	2.1	1.4	271	p3. p2. p3
Lass4	1.2	1	294	p2. p0
Smyd1	1.2	0.9	310	p0. p2. p0
Hip1r	1	0.8	339	p2. p3
Sorl1	1.4	0.8	357	p3. p2
Plxnc1	1.5	0.9	413	p2. p0. p1
Cpox	1.1	0.7	472	p2. p1
Dynlt3	1.3	0.7	487	p2. p3. p1
Cd8a	1	0.6	615	p2. p0. p0. p0
Nfat5	1.2	0.8	626	p2. p2. p3. p0. p2. p2

HP2 - Down regulated genes

Gene	FluH-NaH	TimH-NaH	RankMeta	Classe profils
Stk3	-1.2	-1.5	-727	p2m
Sh3bp5	-1	-1	-653	p2m
Itk	-1.1	-0.9	-486	p2m
2810410L24Rik	-1.1	-1	-474	p2m
Tuba1a	-1	-0.7	-461	p2m
Tsc22d3	-1.2	-1	-392	p2m
Abcg2	-1.4	-1.2	-389	p2m
Ppm1l	-1.2	-0.7	-355	p2m
Fnbp1l	-1.7	-1.9	-324	p2m
Snn	-1.1	-0.7	-322	p2m
Cux1	-1.5	-1.2	-314	p2m
Txk	-1.2	-0.9	-313	p2m
Chd2	-1.1	-0.8	-308	p2m

Arrb1	-1	-1	-303	p2m
Ptpla	-1.1	-0.7	-284	p2m
Cyp39a1	-1	-1.2	-283	p2m
Plcxd2	-1.2	-1.3	-280	p2m
Aldh2	-1.2	-0.8	-275	p2m
Ccm2	-1.1	-0.8	-266	p2m
Setd7	-1.2	-0.8	-260	p2m
Ggta1	-1	-1.2	-256	p2m
Dusp1	-1.5	-1.4	-242	p2m
Dapk1	-1.1	-0.8	-225	p2m
Timeless	-1.2	-0.8	-221	p2m
Rab4a	-2.7	-2.3	-207	p2m
Rhebl1	-1.1	-1	-193	p2m
Ppm1h	-1.2	-0.7	-188	p2m
Nedd4l	-1	-0.8	-186	p2m
Gprc5b	-1.1	-1	-183	p2m
Irf6	-1.1	-1.6	-172	p2m
Bend5	-1	-1.2	-164	p2m
Pard6g	-1.6	-1.4	-159	p2m
Sqrdl	-1.4	-1.1	-153	p2m
Laptm4b	-1.8	-1.8	-148	p2m
Cnksr3	-1.8	-1.5	-138	p2m
Ramp3	-1.8	-1.6	-136	p2m
Pou2af1	-1.5	-1.1	-127	p2m
Nrip1	-1.2	-1.3	-120	p2m
Abca1	-1.3	-1	-118	p2m
Kif13a	-1.5	-1.4	-111	p2m
Gtf2ird1	-1.1	-1	-104	p2m
Zdhhc14	-1.7	-1.7	-101	p2m
Tcf7l2	-2.1	-2	-99	p2m
Scml4	-1	-0.6	-98	p2m
Spnb2	-1.4	-1	-96	p2m
2410066E13Rik	-1.5	-1.3	-92	p2m
Cd81	-1.5	-1.5	-88	p2m
Tubb2a	-2	-2.2	-74	p2m
Sfmbt2	-1.4	-2	-68	p2m
Sepp1	-1.5	-1.3	-64	p2m
Basp1	-2.9	-2.8	-62	p2m
Dusp10	-1.7	-1.3	-60	p2m
Ldhb	-2.8	-2.9	-59	p2m
Baz2b	-2.6	-2.6	-56	p2m
Ralgps2	-1.9	-1.4	-47	p2m
Tubb2b	-4.2	-3.6	-44	p2m
Plaur	-1.6	-1.6	-43	p2m
Sesn1	-1.7	-1.5	-42	p2m
Sgk3	-1.3	-1.2	-41	p2m
Cxxc5	-1.5	-1.2	-38	p2m
Gpr146	-1.3	-1.3	-35	p2m
Plekho1	-1.4	-1.2	-31	p2m
Ppic	-2	-1.5	-29	p2m
Gsn	-1.5	-1.3	-27	p2m
Lass6	-1	-0.7	-26	p2m
Ramp1	-1.8	-1.6	-25	p2m
Cd24a	-2.8	-3.6	-24	p2m
Mtss1	-1.9	-2.2	-20	p2m
Trat1	-2.4	-2.6	-18	p2m
Sox4	-4.7	-5.3	-16	p2m
Timp2	-2	-2.2	-12	p2m
Xkrx	-1.9	-1.8	-9	p2m
Itgae	-1.9	-2.2	-7	p2m
Utp14b	-1.7	-1.6	0	p2m

LOC552908	-1.8	-1.5	0	p2m
A230058F20Rik	-1	-1	0	p2m
C230085N15Rik	-1.4	-1	0	p2m
Ptprf	-1.1	-1	0	p2m
9430027B09Rik	-1.3	-0.9	0	p2m
Gm19597	-1.2	-0.9	0	p2m
E430014B02Rik	-1.2	-0.8	0	p2m
2310050P20Rik	-1.1	-0.8	0	p2m
Rasgrp2	-1	-0.8	-336	p2m. p1m. p1m
Bend4	-1.1	-0.8	-222	p0. p2m
Hivep3	-1.9	-2	-209	p2m. p0
Pde4b	-1	-0.8	-173	p2m. p3m
Cmah	-1.1	-0.7	-170	p3m. p3m. p3m. p2m
Dusp16	-1.3	-1.2	-158	p2m. p0
Satb1	-1.1	-1	-147	p2m. p2m. p2m. p2m. p1m
Tspan13	-1.2	-0.8	-103	p2m. p3m
Adk	-1.5	-0.9	-58	p2m. p3m. p1m. p1m
Tspan32	-1.2	-0.7	-51	p3m. p2m
Inadl	-2.4	-1.9	-40	p2m. p2m. p3m. p3m
Acsl3	-2.7	-2.2	-37	p2m. p2m. p0
Tox	-1.6	-1.4	-33	p2m. p0
Chst15	-2.1	-1.5	-22	p2m. p0
Il6st	-1.2	-0.9	-19	p2m. p2m. p1m. p1m
Dntt	-1.5	-1.3	-17	p3m. p3m. p2m
Ikzf2	-3.2	-3.8	-11	p6m. p2m. p2m
Igfbp4	-2.4	-1.3	-4	p2m. p3m. p2m
Atp1b1	-1.9	-1.6	-2	p3m. p2m. p3m. p3m
Ccr9	-3.9	-3.5	-1	p3m. p2m. p2m
Cap1	-1.5	-1.4	0	p2m. p0
Tmlhe	-1.3	-1.1	0	p3m. p3m. p3m. p2m

SUPPLEMENTARY TABLE S5. Genes with a fold change of at least 2 in the FLU-TM to Naive comparison and belonging to one of the six following profiles HP3 are shown. FC for the TIM to naive comparison are given. If the gene was found in the meta-analysis (tables S9, 10 and 11) its rank is given. For certain genes multiple probe sets are present on the Affymetrix microarrays, the profile for each probe set is given.

HP3 - Upregulated genes

Gene	FluH-NaH	TimH-NaH	RankMeta	Classe profils
W91776	1.2	0.5	0	p3
Top2a	1.4	0.6	0	p3
Rnf128	1.6	0.6	0	p3
Ddx43	1.9	0.7	0	p3
6530402F18Rik	1.2	0.7	0	p3
B830008J18Rik	1.8	0.7	0	p3
Gm14446	1.8	1	0	p3
6330509M05Rik	2.7	1.5	0	p3
Klrc1	9.5	6.3	1	p3
Ccl5	7.3	5.6	2	p3
S100a6	6.4	5.6	3	p3
Cxcr3	6.5	6	4	p3
Itgax	4	2.2	8	p3
Klrk1	5.7	4.2	9	p3
Fasl	6.3	3.7	10	p3
Cd44	7.2	4.4	11	p3
Il18rap	3.6	2.3	12	p3
Klrc2	6.1	2.9	13	p3
Tbx21	4.7	3.4	14	p3
S100a4	4.1	1.2	15	p3
Gzmk	6.1	1.6	16	p3
Kcnj8	2.3	0.5	17	p3
Casp1	4.4	2.1	18	p3
Runx2	5.4	2.6	20	p3
Gzmb	4.1	2.1	21	p3
Bhlhe40	3.7	1.4	23	p3
Nt5e	4.6	2	24	p3
Ctla2a	2.6	1.7	27	p3
Klrg1	1.6	0.9	28	p3
Ctla2b	3.3	1.8	29	p3
Hopx	3	2.1	31	p3
Dmrta1	3.5	0.8	32	p3
5430427O19Rik	4.4	3.4	33	p3
Casp4	4.2	2.1	35	p3
Myo1f	3.2	1.8	36	p3
Klrc3	4.9	1.9	38	p3
Rgs1	3.9	2	39	p3
Ahnak	3.2	1.9	40	p3
Cxcr6	3.4	0.9	45	p3
Camk2n1	3.1	2.2	47	p3
Il18r1	2.7	1.4	49	p3
Anxa2	2.5	1.3	51	p3
Gnptab	3.5	2.2	56	p3
Cdh1	4.2	1.8	57	p3
St3gal6	3.2	1.5	58	p3
Lgals1	2.1	1.4	59	p3
Fam129a	2.9	1.5	61	p3
H2-Q10	2	1.1	64	p3
Chsy1	2.9	1.3	68	p3
Mdfic	4.7	2.5	73	p3
Ell2	4.3	2.5	77	p3
Sdcbp2	3.3	2.8	89	p3

Acot7	2	1.1	90	p3
F2r	1.9	1	93	p3
Gzma	2.7	1.2	101	p3
Gng2	2.3	1.1	110	p3
Arhgap26	1.6	0.7	112	p3
Antxr2	2.9	1.1	118	p3
Csda	1.9	1.2	119	p3
Prss12	1.7	0.9	126	p3
Capn2	1.7	0.9	128	p3
Cst7	1.6	1	140	p3
Nkg7	2.2	1	143	p3
Tnfrsf1b	2.1	0.7	145	p3
Acsbg1	1.6	0.7	147	p3
Lax1	1.8	1.2	167	p3
Gbp3	2.7	0.9	168	p3
Coro2a	1.6	0.7	169	p3
Cldn12	2.6	1.6	170	p3
Ptpn13	1.5	0.8	171	p3
Snx10	1	0.6	179	p3
Odc1	2.7	1.7	182	p3
2010002N04Rik	2.7	0.8	190	p3
1110067D22Rik	2.6	1.1	196	p3
Jdp2	1.8	0.6	199	p3
Serpinb6b	2.1	0.9	200	p3
Mx1	2.4	0.7	202	p3
Mapkapk3	2.3	1.1	208	p3
Nfil3	2	0.9	210	p3
Stk39	1.4	0.7	211	p3
Fam129b	2.4	0.7	218	p3
Slc39a4	1.5	0.9	219	p3
Gvin1	1.9	1.4	221	p3
4930486L24Rik	2	0.8	224	p3
Inpp5d	1.8	0.8	228	p3
Cd82	1.3	0.5	237	p3
Sh2d2a	1.7	0.7	240	p3
Dstn	1.4	0.7	250	p3
Racgap1	1.8	0.8	252	p3
Usp18	2.1	0.9	254	p3
Ifit3	2.4	1.1	262	p3
Glrx	1.8	0.8	269	p3
Plac8	3	2.1	303	p3
Lxn	1.4	0.9	315	p3
Ikzf3	1.8	1	320	p3
Serpinb6a	2.7	1.4	322	p3
Carhsp1	1.4	0.7	328	p3
Fam110a	1.8	0.8	345	p3
Stap1	1.5	0.6	353	p3
Lcp1	1.4	1	362	p3
Pik3cg	1.2	0.6	363	p3
2010111I01Rik	1.5	0.9	379	p3
Gpr68	1.6	1	380	p3
Ifi203	1.8	0.7	381	p3
Rnf19b	1.4	0.8	485	p3
Cd2	1.1	0.4	539	p3
Capg	1.5	0.6	554	p3
Impa2	1.7	0.5	573	p3
Aars	1	0.5	610	p3
Blcap	1	0.5	630	p3
Aen	1.3	0.7	709	p3
Plek	5.2	1.4	6	p3, p1, p1
Ccr5	3.7	1.3	7	p3, p1, p1

Id2	4.1	1	19	p3, p3, p1
Itgb1	3	1.2	34	p3, p3, p1
Eomes	5	1.6	42	p3, p1
Cobll1	4.3	2	43	p3, p1
Nrp1	2.1	1.2	50	p3, p2, p3, p2
Kcnk5	2.3	0.5	52	p3, p0
Car5b	2.1	0.8	67	p3, p1
Endod1	3.8	2.7	71	p3, p3, p3, p1
Chst11	2	1	72	p3, p0
Adam19	2.6	2.2	80	p2, p3
Atf6	2.4	1.6	85	p3, p3, p0
Ccdc50	2	1	91	p3, p3, p2, p1
Plscr1	3.8	2.5	105	p3, p3, p0
Ms4a6d	1.3	0.6	117	p3, p1
Dock9	1.4	0.8	137	p3, p2, p0
Abcb1a	2.2	0.7	139	p3, p1
Tmem2	2	0.7	141	p3, p1, p1
Hs3st3b1	3	0.9	144	p3, p1
Atxn7l1	1.5	0.6	149	p3, p0
Alcam	1.6	0.7	161	p3, p1, p1, p1, p1
Golim4	2.8	1.4	162	p3, p0
Smad3	1.2	0.4	166	p3, p1
Gramd1b	1.9	1	172	p3, p1
Nfatc2	2.3	0.8	173	p3, p1
Rgs3	1.6	1	174	p3, p3, p1
Cpd	4.7	2.4	186	p3, p3, p3, p0, p1, p1, p1
Bcl2	1.7	0.9	216	p3, p0
Stx11	1.9	1	227	p3, p1
Dnaja4	2.1	1.3	232	p3, p3, p2
B4galt5	1.2	0.6	264	p3, p0
Abi2	1.5	0.7	270	p3, p1
Icos	2.5	1.6	271	p3, p2, p3
Casp3	1.5	0.8	273	p3, p1
BC016495	2.3	1.1	278	p0, p3, p1
Frmd4b	3.8	1.1	333	p3, p1, p1
Hip1r	1.4	0.9	339	p2, p3
Sorl1	1.2	0.6	357	p3, p2
Litaf	2.2	0.5	391	p3, p1
Cd86	2.6	1.3	421	p3, p0
Vps54	1.6	0.8	427	p3, p1, p0, p1
St6galnac4	1.6	0.9	435	p3, p1
Cd47	1.5	0.6	445	p3, p0, p1, p1
Dynlt3	1.5	0.7	487	p2, p3, p1
Nfat5	1.3	0.8	626	p2, p2, p3, p0, p2, p2

HP3 - Downregulated genes

Gene	FluH-NaH	TimH-NaH	RankMeta	Classe profils
Pcsk1	-1.3	-0.9	-289	p3m
Pank4	-1.1	-0.5	-219	p3m
Ncf1	-1.3	-0.5	-139	p3m
Stambpl1	-1.5	-0.8	-124	p3m
Tec	-1.3	-0.6	-121	p3m
Hdac4	-1.5	-1	-106	p3m
Rras2	-2.4	-1.2	-79	p3m
Nipal1	-1.8	-1.1	-71	p3m
Pik3ip1	-1.9	-0.9	-67	p3m
Adcy6	-1.4	-1.1	-57	p3m
Sell	-2.3	-0.7	-49	p3m
5730508B09Rik	-2.4	-0.8	-48	p3m
Afap1	-2.3	-1.4	-46	p3m
Lef1	-2.8	-1.4	-34	p3m

Igf1r	-2.8	-1.1	-32	p3m
2610019F03Rik	-2.3	-1.3	-23	p3m
Id3	-2.2	-0.6	-13	p3m
Ephx1	-2.3	-1.3	-6	p3m
Ifngr2	-2.8	-2.3	-5	p3m
St6gal1	-2.6	-0.9	-3	p3m
AI131651	-2.8	-2.1	0	p3m
BB163080	-2.7	-1.2	0	p3m
Gm20033	-1.6	-1	0	p3m
6720418B01Rik	-1.7	-1	0	p3m
Bambi-ps1	-3.8	-1	0	p3m
Gm10672	-1.3	-0.8	0	p3m
Klf3	-1.9	-0.6	-236	p3m, p1m, p1m, p1m, p1m, p1m
Klf7	-1.9	-0.5	-185	p3m, p1m, p1m, p1m
Pde4b	-1.2	-0.7	-173	p2m, p3m
Cmah	-1.4	-0.8	-170	p3m, p3m, p3m, p2m
Map4k2	-1.1	-0.5	-163	p3m, p0
Fam134b	-1.2	-0.7	-157	p0, p3m
Tspan13	-1.3	-0.6	-103	p2m, p3m
Adk	-1.8	-0.9	-58	p2m, p3m, p1m, p1m
Slc43a2	-1.5	-0.6	-55	p3m, p1m, p1m
Tspan32	-1.3	-0.9	-51	p3m, p2m
Inadl	-1.9	-0.8	-40	p2m, p2m, p3m, p3m
Dntt	-4.2	-3.4	-17	p3m, p3m, p2m
Cnn3	-4.6	-3.5	-8	p3m, p3m, p3m, p3m, p3m, p0
Igfbp4	-1.9	-1.1	-4	p2m, p3m, p2m
Atp1b1	-4.5	-3.5	-2	p3m, p2m, p3m, p3m
Ccr9	-4.8	-4.2	-1	p3m, p2m, p2m
Tmlhe	-2.6	-1.7	0	p3m, p3m, p3m, p2m

SUPPLEMENTARY TABLE S6. Genes with a fold change of at least 2 in the FLU-TM to Naive comparison and belonging to one of the six following profiles RP1 are shown. FC for the TIM to naive comparison are given. If the gene was found in the meta-analysis (tables S9, 10 and 11) its rank is given. For certain genes multiple probe sets are present on the Affymetrix microarrays, the profile for each probe set is given.

RP1 - Upregulated genes

Gene	FluR	FluH	TimR	TimH	Classe profils
Il21	6.4	0	0	0	p1
Zeb2	4.3	1.5	0	0	p3, p1, p1, p1
S100a4	3.9	4.1	0	1.2	p1
Hspa1b	3.8	3.1	0	0	p1
Hspa1a	3.8	3.5	0	0	p1
Ccl9	3.7	1.3	0	0	p1
Ccr2	3.2	2.2	0	0	p1
Lancl3	3.2	2.3	0	0	p1
Ifitm3	3.2	2.7	0	0	p1
Id2	3.1	4.1	0	1	p1, p1, p0
6230424C14Rik	3.1	3.5	0	0	p0, p1, p1
Dusp4	3	1.8	0	0	p1
Serpina3g	3	2.6	0	0	p1
Ifitm1	3	3.6	0	0	p1
Ccl1	3	0	0	0	p1
Mt1	2.9	1.8	0	0	p1
Lgals3	2.9	2.8	0	0	p1
Yes1	2.9	3.1	0	0	p1
Fgl2	2.9	4.7	0	0	p0, p1
Anxa1	2.8	3	0	0	p1
Lmnb1	2.7	2.4	0	0	p1
Itga1	2.6	3	0	0	p1
Ptprj	2.6	1.9	0	0	p1, p1, p1, p3
Socs2	2.5	1.7	0	0	p1
Plagl1	2.5	2.3	0	0	p1
Socs3	2.5	0	0	0	p3, p1, p1
Mpzl2	2.5	0	0	0	p3, p1, p1
Ighm	2.5	0	0	0	p0, p1
Il10	2.4	0	0	0	p1
Itgav	2.4	0	0	0	p3, p3, p1
Litaf	2.3	2.2	0	0.5	p1
Cdh1	2.3	4.2	0	1.8	p1
A630035D09Rik	2.3	1.2	0	0	p1
Zdhhc2	2.2	2	0	0	p1
Abcb1b	2.2	2.4	0	0	p1
Tiparp	2.1	1.2	0	0	p1
2310031A07Rik	2.1	1.6	0	0	p1
Bcl2l1	2.1	1.4	0	0	p3, p3, p1, p1
Prdm1	2	1.1	0	0	p1
2310079F09Rik	2	1.1	0	0	p1
Slc30a1	2	0	0	0	p1
Osbpl3	2	1.2	0	0	p1, p3
Stx11	1.9	1.9	0	1	p1
Galnt3	1.9	0	0	0	p1
Reck	1.9	1.9	0	0	p1
Ahr	1.9	2.2	0	0	p1
Mtmr7	1.9	2.2	0	0	p1
Rrm2	1.9	0	0	0	p1
BB166591	1.9	0	0	0	p1

Rai14	1.9	0	0	0	p1
Scd2	1.8	0	0	0	p1
Cdc42ep3	1.8	1.2	0	0	p1
Cd7	1.8	1.4	0	0	p1
Cish	1.8	1.8	0	0	p1
Capg	1.7	1.5	0	0.6	p1
Antxr2	1.7	2.9	0	1.1	p1
Gm2695	1.7	0	0	0	p1
Klf4	1.7	1.1	0	0	p1
Sema4f	1.7	2.1	0	0	p1
Cpd	1.7	1.9	0	0	p3, p3, p3, p1, p0, p1, p3
Coro2a	1.6	1.6	0	0.7	p1
Bag3	1.6	1.2	0	0.8	p1
BC005685	1.6	0	0	0	p1
Gata3	1.6	1.1	0	0	p1
Perp	1.6	1.3	0	0	p1
Fcgr2b	1.6	1.6	0	0	p1
Baiap3	1.6	1.8	0	0	p1
Dennd5a	1.6	2.2	0	0	p1
Galm	1.6	0	0	0	p1
Mt2	1.6	0	0	0	p1
Tob1	1.6	0	0	0	p1
Itga4	1.6	1.2	0	0.7	p1, p1, p0, p1, p1
Golim4	1.6	0	0	0	p3, p1
Rora	1.6	2.2	0	0	p0, p1, p1, p1, p1, p1, p1
Themis	1.6	0	0	0	p3, p1
Fam129b	1.5	2.4	0	0.7	p1
Dmrta1	1.5	3.5	0	0.8	p1
Klrg1	1.5	1.6	0	0.9	p1
E330009J07Rik	1.5	0	0	0	p1
Slc15a3	1.5	1.2	0	0	p1
Acpl2	1.5	1.3	0	0	p1
Csf2	1.5	0	0	0	p1
Cxcl10	1.5	0	0	0	p1
Gnaq	1.5	0	0	0	p1
Efr3b	1.5	0	0	0	p1
Slco3a1	1.5	1.2	0	0	p3, p1, p3
Mx1	1.4	2.4	0	0.7	p1
Mapkapk3	1.4	2.3	0	1.1	p1
Itgam	1.4	0	0	0	p1
Bcor	1.4	0	0	0	p1
Dyrk3	1.4	1.1	0	0	p1
Ptger4	1.4	1.4	0	0	p1
Gzmm	1.4	1.4	0	0	p1
Smpdl3b	1.4	1.7	0	0	p1
Ret	1.4	1.9	0	0	p1
Car2	1.4	2.2	0	0	p1
Pdzk1	1.4	0	0	0	p1
Fes	1.4	0	0	0	p1
Abcb1a	1.4	2.2	0	0.7	p1, p3
Myo1f	1.4	2.3	0	1.3	p1, p3
Iglv1	1.4	1.1	0	0	p1, p0
Akap13	1.4	0	0	0	p2, p1
D13Ertd324e	1.3	0	0	0	p1
Sema4d	1.3	0	0	0	p1
Tulp4	1.3	0	0	0	p1
Tnfaip3	1.3	1.1	0	0	p1

Hip1	1.3	1.2	0	0	p1
Arhgap21	1.3	1.1	0	0	p1
Kif23	1.3	1.3	0	0	p1
Esm1	1.3	1.4	0	0	p1
A930041H05Rik	1.3	1.5	0	0	p1
1700025G04Rik	1.3	1.6	0	0	p1
Lmna	1.3	2.2	0	0	p1
Cited2	1.3	0	0	0	p1
Ubash3a	1.3	0	0	0	p1
Gm14005	1.3	0	0	0	p1
4930523C07Rik	1.3	0	0	0	p1, p3, p0, p1
Pde7a	1.3	0	0	0	p3, p3, p1, p3
Cd80	1.3	1.6	0	0	p0, p1
Top2a	1.2	1.4	0	0.6	p1
Stk39	1.2	1.4	0	0.7	p1
Cst7	1.2	1.6	0	1	p1
Mapkapk2	1.2	0	0	0	p1
Cx3cr1	1.2	0	0	0	p1
Fuca2	1.2	0	0	0	p1
Gca	1.2	0	0	0	p1
Wdr95	1.2	0	0	0	p1
Pik3ap1	1.2	1.1	0	0	p1
Gpr65	1.2	1.2	0	0	p1
Ifitm2	1.2	1.6	0	0	p1
Cxcr4	1.2	1.7	0	0	p1
Smad7	1.2	0	0	0	p1
5031425E22Rik	1.2	0	0	0	p1
Jmy	1.2	0	0	0	p1
BB165335	1.2	0	0	0	p1
Mylip	1.2	0	0	0	p1
Rnf19b	1.2	1.4	0	0.8	p3, p1
Fbxo30	1.2	0	0	0	p1, p1, p0
Ttc39c	1.2	1.1	0	0	p1, p1, p3
Gprin3	1.2	1.4	0	0	p1, p0
Abi2	1.2	1.4	0	0	p3, p1
Prss12	1.1	1.7	0	0.9	p1
Cd97	1.1	0	0	0	p1
AW555355	1.1	0	0	0	p1
Appl2	1.1	0	0	0	p1
Prr5l	1.1	0	0	0	p1
Optn	1.1	0	0	0	p1
Tsc22d2	1.1	0	0	0	p1
Tnfrsf25	1.1	0	0	0	p1
3830612M24	1.1	1	0	0	p1
Dennd4a	1.1	1.2	0	0	p1
Ptgfrn	1.1	1.1	0	0	p1
Mapk12	1.1	1.1	0	0	p1
Txnrd1	1.1	1.3	0	0	p1
Il2rb	1.1	1.4	0	0	p1
Nek6	1.1	1.5	0	0	p1
Adam9	1.1	1.4	0	0	p1
Jun	1.1	0	0	0	p1
3110027N22Rik	1.1	0	0	0	p1
Spry2	1.1	0	0	0	p1
Syne2	1.1	0	0	0	p1
Mllt6	1.1	0	0	0	p1
Rhoc	1.1	0	0	0.5	p1, p0
Ahnak	1.1	1.7	0	0.7	p1, p3

Bcl2	1.1	1.7	0	0.9	p1, p0
Nek7	1.1	0	0	0	p1, p0
Cd86	1.1	0	0	0	p3, p1
Fryl	1.1	1	0	0	p1, p0, p0
Jak2	1.1	1.4	0	0	p0, p1
Arsb	1.1	0	0	0	p3, p1
Iqgap2	1	0	0	0	p1
Fam26f	1	0	0	0	p1
Rnf216	1	1.1	0	0	p1
Baz1a	1	1.1	0	0	p1
Pygl	1	1.2	0	0	p1
Tanc2	1	1.2	0	0	p1
Gpr160	1	2.4	0	0	p1
Gpr15	1	3.2	0	0	p1
Ado	1	0	0	0	p1
Camk2g	1	0	0	0	p1
Rasgef1b	1	0	0	0	p1
Fap	1	0	0	0	p1
N4bp1	1	1.4	0	0.9	p0, p0, p1
Ctnna1	1	1.3	0	0	p0, p1

RP1 - Downregulated genes

Gene	FluR	FluH	TimR	TimH	Classe profils
Id3	-2.2	-2.2	0	-0.6	p1m
Mfhas1	-2.1	-1.1	0	0	p1m
Pou2af1	-2	-1.5	0	-1.1	p1m
Trib2	-2	-1.2	0	0	p1m
St6gal1	-1.9	-2.6	0	-0.9	p1m
5730508B09Rik	-1.6	-2.4	0	-0.8	p1m
Adk	-1.6	-1.8	0	-0.9	p1m, p1m, p0, p0
Ankrd46	-1.4	0	0	0	p1m
A630033H20Rik	-1.4	0	0	0	p1m
Klf7	-1.4	-2.2	0	-0.5	p1m, p0, p1m, p1m
Evi2a	-1.4	0	0	0	p3m, p1m
Gpr18	-1.3	0	0	-0.5	p1m
Tbc1d30	-1.3	0	0	0	p1m
Sema7a	-1.3	0	0	0	p1m
5430434G16Rik	-1.2	0	0	0	p1m
2810403G07Rik	-1.2	0	0	0	p1m
Glrp1	-1.2	0	0	0	p1m
Shmt1	-1.2	0	0	0	p1m
Tspan13	-1.2	-1.3	0	-0.6	p0, p1m
Osbpl9	-1.2	0	0	-0.5	p1m, p3m, p0
AI504432	-1.1	0	0	-0.9	p1m
Kcna3	-1.1	0	0	-0.5	p1m
Tcrb-J	-1.1	0	0	0	p1m
Fam118a	-1.1	0	0	0	p1m
Lck	-1.1	0	0	0	p1m
Il6st	-1.1	-1.2	0	0	p0, p0, p1m, p1m
Calcrl	-1	0	0	-1	p1m, p0
Fam134b	-1	0	0	-0.8	p1m, p3m
Atp6v0b	-1	0	0	0	p0, p1m

SUPPLEMENTARY TABLE S7. Genes with a fold change of at least 2 in the FLU-TM to Naive comparison and belonging to one of the six following profiles RP2 are shown. FC for the TIM to naive comparison are given. If the gene was found in the meta-analysis (tables S9, 10 and 11) its rank is given. For certain genes multiple probe sets are present on the Affymetrix microarrays, the profile for each probe set is given.

RP2 - Upregulated genes

Gene	FluR	FluH	TimR	TimH	Classe profils
Ifng	7.2	7.6	7	6.3	p2
Fasl	5	6.3	4.3	3.7	p2
Cxcr3	4.7	6.5	4.7	6	p2
Gnptab	3.7	3.5	3.4	2.2	p2
Xcl1	3.5	2.4	2.4	0	p2
Tnfsf9	3.3	2.2	1.9	0	p2
5430427O19Rik	3.2	4.4	2.8	3.4	p2
Ms4a4c	3	2.7	3.1	2.8	p2
Kcnk6	3	3.3	2.7	2.7	p2
Alcam	2.8	1.8	2.2	0	p3, p3, p2, p3, p2
Ccdc50	2.7	2.2	2.3	1.6	p2
Sdcbp2	2.7	3.3	2.6	2.8	p2
Gm20265	2.6	2.8	2.3	3.2	p2
Rab39b	2.6	0	2.3	0	p2
Il7r	2.6	1.1	1.7	0	p3, p2
Tbx21	2.5	4.7	2.5	3.4	p2
Mpzl3	2.5	0	1.6	0	p2
Ctla4	2.3	3	2.3	3.4	p2
Cd160	2.3	1.9	2.1	0	p2
BC016495	2.3	1.7	1.6	0	p3, p3, p2
Gm14446	2.2	1.8	1.6	1	p2
Dopey2	2.2	1.5	1.9	0	p2
Phlda1	2.2	0	1.5	0	p2
Dnaja4	2.1	2.3	1.9	1.7	p2
Sidt1	2.1	2.4	1.6	2.5	p2
Eea1	2.1	2.1	1.2	0	p2
2610027H17Rik	2.1	2.2	1.1	0	p2
Chpt1	2.1	1.9	1.7	1.2	p2, p0
Ostf1	2	0	1.7	0.5	p2
Sfi1	2	2.2	2	2.3	p2
Cdc14a	2	1	1.4	0	p2
Rabgap1l	1.9	0	1.5	0.9	p2
Rnasel	1.9	0	1.1	0	p2
Sphk1	1.9	0	1.4	0	p2
Hnrpll	1.9	0	1.3	0	p2, p3
Ctsw	1.8	1.6	1.3	1.3	p2
Ttc39b	1.8	1.9	1.9	1.7	p2
Stat4	1.8	1	1.4	0	p2
Enpp1	1.8	0	1	0	p2, p3
Samhd1	1.7	0	1.3	1.2	p2
Casp3	1.7	1.5	1.6	0.8	p2
F2rl1	1.7	0	1.4	1	p2
Cldn12	1.7	2.6	1.6	1.6	p2
Gimap7	1.7	1.6	2	1.8	p2
Slfn2	1.7	0	1.6	0	p2
Slco4a1	1.7	0	1.9	0	p2
Nfat5	1.7	1.3	1.3	0.8	p2, p3, p2, p3, p3, p2

Csda	1.7	1.9	1.1	1.2	p3, p2
Oxsm	1.7	0	0.9	0	p3, p3, p2
Hs3st3b1	1.6	3	1.2	0.9	p2
Golm1	1.6	1.6	1	1.1	p2
Ppm1j	1.6	1.7	1.1	1.3	p2
Gm13152	1.6	1.9	1.8	1.4	p2
Gvin1	1.6	1.9	1.5	1.4	p2
Pla2g16	1.6	1.8	1.2	1.7	p2
Tnfrsf9	1.6	1.4	1.1	0	p2
Irf8	1.6	1.5	1.4	0	p2
Map3k8	1.6	1.5	1.4	0	p2
Gbp7	1.6	2.3	1.2	0	p2
Il3	1.6	0	0.9	0	p2
Pfkp	1.5	0	1.6	0.4	p2
Gm8369	1.5	0	1.5	0.6	p2
Smpdl3a	1.5	0	1.3	0.6	p2
Klrd1	1.5	0	1.1	0.7	p2
1110067D22Rik	1.5	2.6	0.9	1.1	p2
Fam92a	1.5	1.3	1.3	1.1	p2
Epsti1	1.5	0	1.7	1.3	p2
Sh3bgrl	1.5	0	1	0	p2
3830408D07Rik	1.5	0	0.9	0	p2
Gls	1.5	1	1	0	p2
Slain1	1.5	0	0.9	0	p2
Iigp1	1.5	0	1.8	0	p2
Sorl1	1.5	1.4	1.2	0.8	p0, p2
Lass4	1.5	1.2	1.1	1	p2, p0
Fam55c	1.4	0	1.4	0.7	p2
Ube2t	1.4	1.1	1.1	0.9	p2
Trim30d	1.4	1.3	1.6	1.2	p2
Atp11a	1.4	1.6	1.3	1.3	p2
Chsy1	1.4	2.9	0.9	1.3	p2
Cxcr5	1.4	1.6	1.7	2.1	p2
Traf5	1.4	0	1	0	p2
Soat2	1.4	0	0.9	0	p2
Gbp2	1.4	0	0.8	0	p2
Hvcn1	1.4	0	1.3	0	p2
Nfatc2	1.4	2.3	0.8	0.8	p2, p0
Stat1	1.4	0	1.1	1.9	p2, p2, p0
Cd8a	1.4	0	1.1	0	p3, p2, p2, p2
Spata13	1.4	0	0.8	0	p3, p2
Ggh	1.3	1.4	0.7	0.8	p2
Lpxn	1.3	1.3	1	0.9	p2
Capn2	1.3	1.7	0.7	0.9	p2
Efhd2	1.3	1.8	1	1.4	p2
Zfpm1	1.3	0	0.9	0	p2
Cd72	1.3	1	0.9	0	p2
Hexb	1.3	0	0.8	0	p2
Gm2a	1.3	0	0.9	0	p2
Vps54	1.3	1.6	0.9	0.8	p2, p0, p0, p2
Gsto1	1.3	1.6	0.8	1.2	p0, p2
Stk38l	1.3	0	1.1	0	p2, p0
Ppp3cc	1.2	0	0.8	0.7	p2
Pttg1	1.2	0	1.1	0.7	p2
Ap1s2	1.2	1	1	0.7	p2

Ms4a6d	1.2	1.3	0.9	0.6	p2
B830008J18Rik	1.2	1.8	0.7	0.7	p2
Ifi47	1.2	0	1.7	0.9	p2
Rab23	1.2	0	1.4	0.9	p2
Dennd4c	1.2	1.5	0.6	1	p2
Fgf13	1.2	1.6	1.2	1.2	p2
Cd48	1.2	0	1	0	p2
Slc2a3	1.2	0	0.9	0	p2
Ptpn4	1.2	0	0.9	0	p2
5730469M10Rik	1.2	1	1.3	0	p2
Anp32a	1.2	0	0.8	0	p2
Gramd3	1.2	0	0.6	0	p2
Oxr1	1.2	0	0.8	0	p2
Rbl2	1.2	0	0.8	0	p2
Ccng2	1.2	0	0.9	0	p2
Sgpp1	1.2	0	0.9	0	p2
Abhd5	1.2	0	0.9	0.6	p2, p0
Tmem2	1.2	2	0.9	0.7	p2, p0, p0
Adora2a	1.2	0	0.7	0	p2, p3
Rgs3	1.2	1.4	0.7	0	p0, p0, p2
Lpgat1	1.2	0	1.3	0	p0, p2
Laptm5	1.1	0	0.9	0.6	p2
Dhrs7	1.1	1.1	0.8	0.7	p2
Tpi1	1.1	1.1	0.8	0.9	p2
Parp14	1.1	0	0.9	0.7	p2
Man2a1	1.1	1.1	0.8	0.7	p2
Myo1d	1.1	1.1	0.7	0.8	p2
Zc3h12d	1.1	0	1.3	1.1	p2
Cks2	1.1	0	1.1	1.9	p2
Aga	1.1	0	0.6	0	p2
Ctsb	1.1	0	0.7	0	p2
Galnt7	1.1	0	0.9	0	p2
St7l	1.1	0	0.8	0	p2
Hgsnat	1.1	1.1	0.9	0	p2
Aim2	1.1	1.1	0.6	0	p2
Lta4h	1.1	0	0.6	0	p2
Lasp1	1.1	0	0.6	0	p2
Zfp703	1.1	0	0.6	0	p2
Wipf1	1.1	0	0.7	0	p2
Prps2	1.1	0	0.7	0	p2
Plxnc1	1.1	1.5	1	0.9	p2, p0, p0
St3gal6	1.1	2.1	0.7	1	p2, p3
Vamp7	1.1	0	0.6	0	p2, p0
2410127L17Rik	1.1	0	0.7	0	p2, p0
Akap13	1.1	0	0.9	0	p2, p1
Lnx2	1	0	0.8	0.5	p2
Slu7	1	0	0.8	0.6	p2
Dym	1	0	0.6	0.6	p2
Cyp4v3	1	0	0.9	0.7	p2
Tbc1d12	1	1.5	0.9	0.8	p2
Lxn	1	1.4	0.8	0.9	p2
Ncf4	1	1.6	0.8	1.1	p2
Dnajc3	1	0	0.8	0	p2
Ccpg1	1	0	1	0	p2
Rassf5	1	0	0.6	0	p2

Adcy7	1	0	0.7	0	p2
Plk1s1	1	0	0.8	0	p2
N4bp2l1	1	0	0.8	0	p2
1500031L02Rik	1	0	0.9	0	p2
Klhl24	1	0	0.9	0	p2
Gbp8	1	0	1.1	0	p2
Slfn1	1	0	1.2	0	p2
Tmem38b	1	0	0.7	0.6	p2, p0
Clic4	1	2.2	0.9	1.6	p0, p2
Ugp2	1	0	0.9	0	p2, p0

RP2 downregulated genes

Gene	FluR	FluH	TimR	TimH	Classe profils
Hivep3	-4.8	-1.9	-4.7	-2	p2m
Ccr9	-4.7	-4.8	-4.1	-4.2	p2m
Atp1b1	-4	-4.5	-3	-3.5	p2m
F3	-3.4	0	-3.2	0	p2m
Sox4	-3.3	-4.7	-4.1	-5.3	p2m, p0
Cd24a	-3.1	-2.8	-3.9	-3.6	p2m
Acsl3	-3.1	-2.7	-2.3	-2.2	p2m
Tubb2b	-3	-4.2	-2.7	-3.6	p2m
Plaur	-3	-1.6	-2.6	-1.6	p2m
Basp1	-2.8	-2.9	-2.9	-2.8	p2m
Baz2b	-2.7	-2.6	-2.5	-2.6	p2m, p2m, p0, p2m
Itm2a	-2.6	0	-2.8	-1.8	p2m
Ldhb	-2.4	-2.8	-2.7	-2.9	p2m
Fnbp1l	-2.1	-1.7	-2	-1.9	p2m
Lipg	-2.1	0	-2.3	0	p2m
Lif	-2.1	0	-1.8	0	p2m
Ramp3	-2	-1.8	-1.5	-1.6	p2m
9430027B09Rik	-2	-1.3	-1.2	-0.9	p2m
Tcf7l2	-2	-2.1	-2	-2	p2m, p2m, p0
AI131651	-1.9	-2.8	-1.8	-2.1	p2m
Afap1	-1.9	-2.3	-1.3	-1.4	p2m
A130001G05Rik	-1.9	-1.1	-1.2	0	p2m
Cdk5r1	-1.9	0	-1.3	0	p2m
Igfbp4	-1.9	-2.4	-1.3	-1.3	p2m, p0, p0
Chst15	-1.9	0	-1.5	-0.6	p3m, p2m
Myb	-1.9	-1.5	-1.1	0	p2m, p2m, p3m
Ctse	-1.8	-2.9	-2.6	-3.8	p2m
Trat1	-1.8	-2.4	-2.1	-2.6	p2m
Cnksr3	-1.8	-1.8	-1.4	-1.5	p2m
Cap1	-1.8	-1.5	-1.5	-1.4	p2m
Ptpla	-1.8	-1.1	-1.6	-0.7	p2m
Sgip1	-1.8	0	-1.6	0	p2m
Timp2	-1.8	-2	-1.9	-2.2	p2m, p0, p0
P4ha2	-1.7	0	-1.8	-0.5	p2m
Mast4	-1.7	0	-1.5	0	p2m
Bach1	-1.7	0	-1	0	p2m
Dntt	-1.7	-1.5	-1.2	-1.3	p3m, p3m, p2m
Tdrkh	-1.6	0	-1.4	0	p2m
Itgae	-1.5	-1.9	-1.8	-2.2	p2m
Tubb2a	-1.5	-2	-1.1	-2.2	p2m
Sfmbt2	-1.5	-1.4	-1.8	-2	p2m
Vegfa	-1.5	0	-1.1	0	p2m

4930426L09Rik	-1.5	0	-1	0	p2m
Ikzf2	-1.5	-3.2	-2	-3.8	p6m, p2m, p0
Zdhhc14	-1.4	-1.7	-1.1	-1.7	p2m
Ramp1	-1.4	-1.8	-1.3	-1.6	p2m
Gsn	-1.4	-1.5	-1	-1.3	p2m
BB163080	-1.4	-2.7	-0.9	-1.2	p2m
Uqcrq	-1.4	0	-1.2	0	p2m
Rorc	-1.4	0	-1.3	0	p2m
Tnfrsf19	-1.4	0	-1.3	0	p2m
Dusp16	-1.3	-1.3	-1.7	-1.2	p2m
C230085N15Rik	-1.3	-1.4	-1.3	-1	p2m
Ets2	-1.3	0	-1.3	-0.7	p2m
2700079J08Rik	-1.3	0	-0.9	-0.6	p2m
Srpk2	-1.3	0	-1.1	-0.6	p2m
Atxn10	-1.3	0	-1	0	p2m
Papss2	-1.3	0	-1.3	0	p2m
Tmcc3	-1.3	0	-1.4	0	p2m
Leap2	-1.3	0	-1.2	0	p2m
Irak3	-1.3	0	-1.1	0	p2m
Satb1	-1.3	-1.3	-1.3	-0.9	p3m, p0, p2m, p2m, p2m
Tox	-1.2	-1.6	-1.5	-1.4	p2m
Frat2	-1.2	0	-1.1	0	p2m
Lbh	-1.2	0	-1.1	0	p2m
Cd5	-1.2	0	-1	0	p2m
Tmlhe	-1.2	-2.6	-0.7	-1.7	p2m, p0, p0, p0
Ifngr2	-1.1	-2.8	-1.2	-2.3	p2m
Gtf2ird1	-1.1	-1.1	-1	-1	p2m
Aldh2	-1.1	-1.2	-1.2	-0.8	p2m
E430014B02Rik	-1.1	-1.2	-1.2	-0.8	p2m
Tmem69	-1.1	0	-1	-0.8	p2m
Skap1	-1.1	0	-1	-0.6	p2m
Zcchc12	-1.1	0	-1	-0.5	p2m
A930002I21Rik	-1.1	0	-0.9	-0.4	p2m
D130062J21Rik	-1.1	0	-0.6	0	p2m
P2rx4	-1.1	0	-0.7	0	p2m
Aldh1b1	-1.1	0	-0.8	0	p2m
Kbtbd7	-1.1	1.1	-1.2	0	p2m
5730419I09Rik	-1.1	0	-1.4	0	p2m
Pex5	-1.1	0	-1.2	0	p2m
Slc16a10	-1.1	0	-1.1	0	p2m
Etnk1	-1.1	0	-1	0	p2m
Mmd	-1.1	0	-0.8	0	p2m
Tacc2	-1.1	0	-0.8	0	p2m
Pdss1	-1.1	0	-0.7	0	p2m
Cnn3	-1.1	0	-0.7	-0.6	p3m, p3m, p3m, p3m, p3m, p2m
Slc43a2	-1.1	-1.5	-0.7	-0.6	p2m, p0, p0
Ankrd28	-1.1	0	-1	0	p2m, p0
Prnp	-1.1	0	-1.5	0	p6m, p2m
Trim13	-1	0	-1	-1.3	p2m
Ggta1	-1	-1	-1.5	-1.2	p2m
Scmh1	-1	0	-0.8	-0.6	p2m
Hectd2	-1	0	-0.8	-0.7	p0, p2m

SUPPLEMENTARY TABLE S8. Genes with a fold change of at least 2 in the FLU-TM to Naive comparison and belonging to one of the six following profiles RP3 are shown. FC for the TIM to naive comparison are given. If the gene was found in the meta-analysis (tables S9. 10 and 11) its rank is given. For certain genes multiple probe sets are present on the Affymetrix microarrays. The profile for each probe set is given.

RP3 - Upregulated gene

Gene	FluR	FluH	TimR	TimH	Classe profils
Klrc1	8.4	9.5	6.1	6.3	p3
Plek	7.1	5.2	4.9	1.4	p3
Cd44	6.9	7.2	5	4.4	p3
Ccl5	6.6	7.3	4.6	5.6	p3
S100a6	6.2	6.4	4.7	5.6	p3
Ccr5	5.6	3.7	3.7	1.3	p3
Klrk1	5.5	5.7	3.9	4.2	p3
Itgax	5.3	4	2.9	2.2	p3
Klrc2	5.2	6.1	2.5	2.9	p3
Il18rap	4.8	3.6	3.2	2.3	p3
Gzmk	4.7	6.1	0.9	1.6	p3
Il18r1	4.5	2.7	2.9	1.4	p3
Eomes	4.5	5	1.6	1.6	p3
Mdfic	4.5	4.7	3.5	2.5	p3
Havcr2	4.3	2	2.5	0	p3
Plscr1	4	3.8	2.8	2.5	p3
Gzmb	4	4.1	2.3	2.1	p3
Runx2	3.9	5.4	2	2.6	p3
Nt5e	3.8	4.6	1.6	2	p3
Sv2c	3.7	1.6	1	0	p3
Ptger2	3.7	2.7	2.3	0	p3
Serpinb6b	3.6	2.1	2.7	0.9	p3
Gbp3	3.5	2.7	1.6	0.9	p3
Klrc3	3.5	4.9	1	1.9	p3
Bhlhe40	3.4	3.7	1.1	1.4	p3
Casp1	3.4	4.4	1.7	2.1	p3
Rgs16	3.4	3.5	2.1	0	p3
Nfil3	3.3	2	2.3	0.9	p3
Casp4	3.3	4.2	1.6	2.1	p3
Serpinb9	3.2	2.5	2.3	0	p3
Spin4	3.1	0	1.6	0	p3
Gzma	3	2.7	1.7	1.2	p3
Vim	2.9	1.9	1.4	0	p3
Zbtb1	2.8	0	1.4	0	p3
Il1rl1	2.8	0	1.1	0	p3
D330040H18Rik	2.8	0	1.6	0	p3
6330509M05Rik	2.7	2.7	1.2	1.5	p3
Atxn7l1	2.6	1.5	1.7	0.6	p3
Cxcr6	2.6	3.4	1.1	0.9	p3
Serpinb6a	2.6	2.7	1.4	1.4	p3
Ctla2b	2.6	3.3	1.7	1.8	p3
Plac8	2.6	3	1.7	2.1	p3
AA467197	2.6	2.6	0.7	0	p3
Chst11	2.4	2	1	1	p3
Anxa2	2.4	2.5	1.2	1.3	p3
Adam19	2.4	2.6	1.7	2.2	p3
Ell2	2.4	4.3	1.2	2.5	p3
Srxn1	2.4	0	1.3	0	p3
Zbtb32	2.4	0	1.1	0	p3
LOC641050	2.3	1.3	1.2	1.1	p3
Fam129a	2.3	2.9	1.4	1.5	p3
Ctla2a	2.3	2.6	1.4	1.7	p3
Rgs1	2.3	3.9	1.4	2	p3
Camk2n1	2.3	3.1	1.6	2.2	p3
Ifih1	2.3	0	0.8	0	p3

Kcnk5	2.2	2.3	0.8	0.5	p3
Smyd1	2.2	1.2	0.9	0.9	p3
Cd47	2.2	1.5	1.2	0.6	p3
Nkg7	2.2	2.2	1	1	p3
Atxn1	2.2	1.6	1.3	1.2	p3
Rnf157	2.2	1.9	1.1	0	p3
4930486L24Rik	2.1	2	1.2	0.8	p3
2010111I01Rik	2.1	1.5	1.2	0.9	p3
Aim1	2.1	2	1	0	p3
Ddx43	2	1.9	1	0.7	p3
Fam46c	2	0	0.9	0.9	p3
Atf6	2	2.4	1.1	1.6	p3
H2-Q10	2	2	1	1.1	p3
Lgals1	2	2.1	1.1	1.4	p3
Hopx	2	3	1.4	2.1	p3
Il10ra	2	1.8	1	0	p3
Usp18	1.9	2.1	1	0.9	p3
Tmbim1	1.9	1.5	0.9	0	p3
Ifi203	1.8	1.8	1.1	0.7	p3
Acot7	1.8	2	1	1.1	p3
Crot	1.8	0	0.9	0	p3
Elk3	1.8	1.2	0.8	0	p3
Rbpj	1.7	1.5	0.7	0	p3
Dusp14	1.7	0	0.6	0	p3
4933415E08Rik	1.7	0	0.8	0	p3
Ifit3	1.6	2.4	0.8	1.1	p3
3110037C07Rik	1.6	0	0.5	0	p3
AI120166	1.6	0	0.9	0	p3
Zbtb25	1.6	0	0.9	0	p3
Cd52	1.5	1	0.8	0.8	p3
Tnfrsf18	1.5	1.1	0.7	0	p3
Arhgap26	1.4	1.6	0.5	0.7	p3
Atp10a	1.4	1.1	0.7	0	p3
Sytl3	1.4	1.6	0.7	0	p3
4930431P03Rik	1.4	0	0.6	0	p3
A430103D13Rik	1.4	0	0.8	0	p3
Nckap1	1.3	0	0.8	0.5	p3
Jdp2	1.3	1.8	0.8	0.6	p3
Slc39a4	1.3	1.5	0.7	0.9	p3
Kbtbd11	1.2	1.1	0.8	0.8	p3
Nfe2l2	1.2	0	0.6	0	p3
Nfatc3	1.2	0	0.6	0	p3
Cd2	1.1	1.1	0.7	0.4	p3
Ksr1	1.1	1	0.6	0.6	p3
Grina	1	0	0.5	0.7	p3
Osbpl3	4.3	3	0.9	0	p1. p3
Mpzl2	3.7	0	0.9	0	p3. p1. p1
BC016495	3.6	2.3	2.8	1.1	p3. p3. p2
Zeb2	3.5	1.1	1.4	0	p3. p1. p1. p1
Cpd	3.4	4.7	1.6	2.4	p3. p3. p3. p1. p0. p1. p3
Ptprj	3.4	2.2	0.9	0	p1. p1. p1. p3
Ttc39c	3.2	3.1	0.9	0	p1. p1. p3
Cobll1	3.1	4.3	1.7	2	p3. p0
Alcam	2.8	1.8	1.5	0.7	p3. p3. p2. p3. p2
Cd86	2.8	2.6	1.5	1.3	p3. p1
Ahnak	2.7	3.2	1.4	1.9	p1. p3
Nrp1	2.6	3.4	1.5	2.6	p3. p3. p0. p3
Socs3	2.6	0	0.7	0	p3. p1. p1
Il7r	2.6	0	1.5	0	p3. p2
Itgav	2.6	1.7	1.4	0	p3. p3. p1
Myo1f	2.5	3.2	0.9	1.8	p1. p3
Itgb1	2.3	3	1.2	1.2	p3. p3. p0
Golim4	2.3	2.8	1.2	1.4	p3. p1
St3gal6	2.2	3.2	1.3	1.5	p2. p3

Hnrpll	2.2	0	1.3	0	p2. p3
Arsb	2	1.1	1.2	0.8	p3. p1
Endod1	2	3.8	1	2.7	p0. p0. p3. p0
Abcb1a	2	2.2	0.9	0	p1. p3
Bcl2l1	1.9	1.2	0.8	0	p3. p3. p1. p1
Slco3a1	1.9	1.3	0.8	0	p3. p1. p3
Nfat5	1.8	1.1	1.2	0.8	p2. p3. p2. p3. p3. p2
Odc1	1.7	2.7	1.1	1.7	p0. p3. p3
Cd8a	1.6	1	1.1	0.6	p3. p2. p2. p2
Oxsm	1.6	0	0.9	0	p3. p3. p2
B4galt5	1.5	1.2	0.9	0.6	p3. p0
Racgap1	1.5	1.8	0.7	0.8	p0. p3
Abi2	1.4	1.5	0.6	0.7	p3. p1
Spata13	1.4	0	0.7	0	p3. p2
Themis	1.4	0	0.5	0	p3. p1
Smad3	1.3	1.2	0.4	0.4	p3. p0
Enpp1	1.3	0	0.6	0	p2. p3
Csda	1.2	1.4	0.8	0.9	p3. p2
Pde7a	1.2	0	0.6	0	p3. p3. p1. p3
Adora2a	1.2	0	0.6	0	p2. p3
Rnf19b	1.1	1.3	0.5	0.8	p3. p1
4930523C07Rik	1	0	0.5	0	p1. p3. p0. p1

RP3 - downregulated

Gene	FluR	FluH	TimR	TimH	Classe profils
Xkrx	-3.6	-1.9	-2.9	-1.8	p3m
Cyp11a1	-3.3	0	-1.3	0	p3m
4930520K10Rik	-3.1	-1.2	-1.9	0	p3m
Utp14b	-3	-1.7	-2	-1.6	p3m
2010007H06Rik	-2.2	0	-1.2	0	p3m
Ephx1	-2.1	-2.3	-1.3	-1.3	p3m
Igf1r	-2.1	-2.8	-1.2	-1.1	p3m
Akap2	-1.8	0	-1.2	-1.1	p3m
Bambi-ps1	-1.6	-3.8	-0.7	-1	p3m
Sh3bp2	-1.6	0	-0.8	0	p3m
Bend4	-1.5	-1.1	-0.8	-0.9	p3m
Rgs10	-1.5	0	-0.7	0	p3m
Sft2d2	-1.2	0	-0.6	0	p3m
Map4k4	-1.2	0	-0.8	0	p3m
Pycr1	-1.2	0	-0.6	0	p3m
Rabggta	-1.1	0	-0.5	0	p3m
Tmem108	-1	0	-0.5	-0.5	p3m
Ldlr	-1	0	-0.5	0	p3m
Ece2	-1	0	-0.5	0	p3m
Cnn3	-4.1	-4.6	-2.7	-3.5	p3m. p3m. p3m. p3m. p3m. p2m
Dntt	-3.9	-4.2	-3.1	-3.4	p3m. p3m. p2m
Chst15	-3	-2.1	-1.9	-1.5	p3m. p2m
Myb	-1.9	-1.3	-1	0	p2m. p2m. p3m
Evi2a	-1.7	0	-0.9	0	p3m. p1m
Osbpl9	-1.4	0	-0.4	0	p1m. p3m. p0
Satb1	-1.3	-1.1	-0.8	-1	p3m. p0. p2m. p2m. p2m
Fam134b	-1.3	-1.2	-0.6	-0.7	p1m. p3m

SUPPLEMENTARY TABLE S9. Meta-analysis was performed using the RankProd on 8 datasets comparing the gene expression signature of memory CD8 T cells compared to naive CD8 T cells in homeostatic conditions. * Fisher enrichment test p-values of discretized profiles (columns) in the differential expression gene lists (Memory versus naive comparison) from studies considered for the Rankprod meta-analysis (rows).

List Name	Pathogen	TCR Transgenic	Time post priming	Reference	GSE file	HP2*	HP3*
VVP1	Vaccinia-NP68	F5	48-56	Platine1	GSE70763	1,36E-164	2,67E-167
LP2	Listeria-NP68	F5	48-54	Platine2	GSE86601	1,54E-90	9,19E-114
LImm1	Listeria-Ova	OT1	45	ImmGen	GSE15907	1,03E-35	2,03E-79
LImm2	Listeria-Ova	0T1	100	ImmGen	GSE15907	1,78E-33	6,83E-71
LW	att-Listeria-Ova	OT1	85-95	Wirth et al 2011	GSE21360	7,94E-59	5,27E-96
VSVImm1	VSV	OT1	45	ImmGen	GSE15907	7,33E-30	3,03E-63
VSVImm2	VSV	OT1	106	ImmGen	GSE15907	5,15E-05	2,49E-17
LCMV	LCMV	P14	60-120	Sarkar et al 2008	GSE10239	3,07E-19	3,51E-26

SUPPLEMENTARY TABLE S10. List of up-regulated genes with their ranking in the meta-analysis performed as described in the Methods. The first column is giving the gene rank according to the gene rank product (the largest FC across studies are associated with the smallest Rank Product). The last column is the average expression levels under two conditions (Memory and Naive) given as a log2 fold change. List of up-regulated genes with their ranking in the meta-analysis performed as described in the methods.

Rank	Name	Description	log2FC(Mem vs Na)
1	Klrc1	killer cell lectin-like receptor subfamily C. member 1	5.92
2	Ccl5	chemokine (C-C motif) ligand 5	5.21
3	S100a6	S100 calcium binding protein A6 (calcyclin)	5.01
4	Cxcr3	chemokine (C-X-C motif) receptor 3	3.93
5	Ifng	interferon gamma	3.83
6	Plek	pleckstrin	3.95
7	Ccr5	chemokine (C-C motif) receptor 5	3.84
8	Itgax	integrin alpha X	3.7
9	Klrk1	killer cell lectin-like receptor subfamily K. member 1	3.8
10	Fasl	Fas ligand (TNF superfamily. member 6)	3.5
11	Cd44	CD44 antigen	3.33
12	Il18rap	interleukin 18 receptor accessory protein	3.56
13	Klrc2	killer cell lectin-like receptor subfamily C. member 2	3.54
14	Tbx21	T-box 21	3.52
15	S100a4	S100 calcium binding protein A4	3.23
16	Gzmk	granzyme K	3.04
17	Kcnj8	potassium inwardly-rectifying channel. subfamily J. member 8	2.96
18	Casp1	caspase 1	2.83
19	Id2	inhibitor of DNA binding 2	3.09
20	Runx2	runt related transcription factor 2	2.79
21	Gzmb	granzyme B	2.93
22	Ccr2	chemokine (C-C motif) receptor 2	3.16
23	Bhlhe40	basic helix-loop-helix family. member e40	2.77
24	Nt5e	5' nucleotidase. ecto	2.77
25	Slamf7	SLAM family member 7	3.04
26	Anxa1	annexin A1	2.99
27	Ctla2a	cytotoxic T lymphocyte-associated protein 2 alpha	2.83
28	Klrg1	killer cell lectin-like receptor subfamily G. member 1	2.62
29	Ctla2b	cytotoxic T lymphocyte-associated protein 2 beta	2.5
30	Ttc39c	tetratricopeptide repeat domain 39C	2.52
31	Hopx	HOP homeobox	2.28
32	Dmrta1	doublesex and mab-3 related transcription factor like family	2.33
33	5430427O19Rik	RIKEN cDNA 5430427O19 gene	2.16
34	Itgb1	integrin beta 1 (fibronectin receptor beta)	2.86
35	Casp4	caspase 4. apoptosis-related cysteine peptidase	2.04
36	Myo1f	myosin IF	2.38
37	Ccl3	chemokine (C-C motif) ligand 3	2.43
38	Klrc3	killer cell lectin-like receptor subfamily C. member 3	2.37
39	Rgs1	regulator of G-protein signaling 1	2.13
40	Ahnak	AHNAK nucleoprotein (desmoyokin)	2.29
41	Fcgr2b	Fc receptor. IgG. low affinity IIb	2.53
42	Eomes	eomesodermin homolog (Xenopus laevis)	1.82
43	Cobll1	Cobl-like 1	2.05
44	Ptprj	protein tyrosine phosphatase. receptor type. J	2.49
45	Cxcr6	chemokine (C-X-C motif) receptor 6	1.97
46	Smpdl3b	sphingomyelin phosphodiesterase. acid-like 3B	2.25
47	Camk2n1	calcium/calmodulin-dependent protein kinase II inhibitor 1	1.95

48	Osbpl3	oxysterol binding protein-like 3	2.51
49	Il18r1	interleukin 18 receptor 1	2.22
50	Nrp1	neuropilin 1	2.41
51	Anxa2	annexin A2	2.15
52	Kcnk5	potassium channel. subfamily K. member 5	2.15
53	Zeb2	zinc finger E-box binding homeobox 2	2.11
54	Il2ra	interleukin 2 receptor. alpha chain	1.82
55	Zdhhc2	zinc finger. DHHC domain containing 2	2.02
56	Gnptab	N-acetylglucosamine-1-phosphate transferase. alpha and beta	1.8
57	Cdh1	cadherin 1	1.78
58	St3gal6	ST3 beta-galactoside alpha-2.3-sialyltransferase 6	1.83
59	Lgals1	lectin. galactose binding. soluble 1	2.12
60	Yes1	Yamaguchi sarcoma viral (v-yes) oncogene homolog 1	1.65
61	Fam129a	family with sequence similarity 129. member A	2.03
62	Itga1	integrin alpha 1	2.32
63	Gzmm	granzyme M (lymphocyte met-ase 1)	1.79
64	H2-Q10	histocompatibility 2. Q region locus 10	1.7
65	Ttc39b	tetratricopeptide repeat domain 39B	1.7
66	1700025G04Rik	RIKEN cDNA 1700025G04 gene	1.91
67	Car5b	carbonic anhydrase 5b. mitochondrial	2.03
68	Chsy1	chondroitin sulfate synthase 1	1.71
69	Xcl1	chemokine (C motif) ligand 1	1.55
70	Entpd1	ectonucleoside triphosphate diphosphohydrolase 1	1.72
71	Endod1	endonuclease domain containing 1	1.68
72	Chst11	carbohydrate sulfotransferase 11	1.56
73	Mdfic	MyoD family inhibitor domain containing	1.79
74	Ppm1j	protein phosphatase 1J	1.62
75	Il12rb2	interleukin 12 receptor. beta 2	1.64
76	Ccl4	chemokine (C-C motif) ligand 4	1.71
77	Ell2	elongation factor RNA polymerase II 2	1.43
78	Tnfsf14	tumor necrosis factor (ligand) superfamily. member 14	1.56
79	Asns	asparagine synthetase	1.47
80	Adam19	a disintegrin and metallopeptidase domain 19 (meltrin beta)	1.44
81	Sytl2	synaptotagmin-like 2	1.55
82	S1pr5	sphingosine-1-phosphate receptor 5	2.04
83	Lrrk1	RIKEN cDNA 5730590G19 gene	1.81
84	Nrarp	Notch-regulated ankyrin repeat protein	1.5
85	Atf6	activating transcription factor 6	1.49
86	Pglyrp1	peptidoglycan recognition protein 1	1.56
87	Crtam	cytotoxic and regulatory T cell molecule	1.45
88	Ly6a	lymphocyte antigen 6 complex. locus A	1.31
89	Sdcbp2	trafficking protein particle complex 4	1.43
90	Acot7	acyl-CoA thioesterase 7	1.41
91	Ccdc50	coiled-coil domain containing 50	1.31
92	Klrb1c	killer cell lectin-like receptor subfamily B member 1C	2.3
93	F2r	coagulation factor II (thrombin) receptor	1.4
94	Aqp9	aquaporin 9	1.66
95	Dennd5a	DENN/MADD domain containing 5A	1.36
96	Soat2	sterol O-acyltransferase 2	1.5
97	5830416P10Rik	RIKEN cDNA 5830416P10 gene	1.33
98	Il10ra	interleukin 10 receptor. alpha	1.49
99	Gata3	GATA binding protein 3	1.27
100	Pogk	pogo transposable element with KRAB domain	1.53
101	Gzma	granzyme A	1.34
102	Slc25a24	solute carrier family 25 (mitochondrial carrier. phosphate c	1.54

103	Rora	RAR-related orphan receptor alpha	1.42
104	Pion	pigeon homolog (Drosophila)	1.38
105	Plscr1	phospholipid scramblase 1	1.4
106	Itpripl2	inositol 1.4.5-triphosphate receptor interacting protein-lik	1.18
107	Il2rb	interleukin 2 receptor. beta chain	1.58
108	Mapre2	microtubule-associated protein. RP/EB family. member 2	1.71
109	Dock5	dedicator of cytokinesis 5	1.42
110	Gng2	guanine nucleotide binding protein (G protein). gamma 2	1.29
111	Gpr183	G protein-coupled receptor 183	1.18
112	Arhgap26	Rho GTPase activating protein 26	1.43
113	Pik3ap1	phosphoinositide-3-kinase adaptor protein 1	1.84
114	Serpinb9	serine (or cysteine) peptidase inhibitor. clade B. member 9	1.1
115	N4bp1	NEDD4 binding protein 1	1.2
116	Kcnk6	potassium channel. subfamily K. member 7	1.21
117	Ms4a6d	membrane-spanning 4-domains. subfamily A. member 6D	1.2
118	Antxr2	anthrax toxin receptor 2	1.1
119	Csda	cold shock domain protein A	1.19
120	Fgf13	fibroblast growth factor 13	1.22
121	Fgl2	fibrinogen-like protein 2	1.37
122	Lancl3	LanC lantibiotic synthetase component C-like 3 (bacterial)	1.17
123	AA467197	expressed sequence AA467197	1.22
124	Efhd2	EF hand domain containing 2	1.35
125	Dkkl1	dickkopf-like 1	1.37
126	Prss12	protease. serine. 12 neurotrypsin (motopsin)	1.26
127	Bcl2l1	BCL2-like 1	1.11
128	Capn2	calpain 2	1.2
129	Ier3	immediate early response 3	1.19
130	Ern1	endoplasmic reticulum (ER) to nucleus signalling 2	1.12
131	Tspan31	tetraspanin 31	1.18
132	Tmem154	transmembrane protein 154	1.22
133	Wfikkn2	WAP. follistatin/kazal. immunoglobulin. kunitz and netrin do	1.22
134	Aim1	absent in melanoma 1	1.11
135	Arl4d	ADP-ribosylation factor-like 4D	1.22
136	Gab3	growth factor receptor bound protein 2-associated protein 3	1.09
137	Dock9	dedicator of cytokinesis 9	1.1
138	Ms4a4c	membrane-spanning 4-domains. subfamily A. member 4C	0.79
139	Abcb1a	ATP-binding cassette. sub-family B (MDR/TAP). member 1A	1.06
140	Cst7	cystatin F (leukocystatin)	1.06
141	Tmem2	transmembrane protein 2	1.08
142	Cx3cr1	chemokine (C-X3-C) receptor 1	1.86
143	Nkg7	natural killer cell group 7 sequence	1.03
144	Hs3st3b1	heparan sulfate (glucosamine) 3-O-sulfotransferase 3B1	1.06
145	Tnfrsf1b	tumor necrosis factor receptor superfamily. member 1b	1.05
146	Adora2a	adenosine A2a receptor	1.14
147	Acsbg1	acyl-CoA synthetase bubblegum family member 1	1.05
148	Raph1	Ras association (RalGDS/AF-6) and pleckstrin homology domain	1.08
149	Atxn7l1	ataxin 7-like 1	1.11
150	Cdc42ep3	CDC42 effector protein (Rho GTPase binding) 3	1.18
151	Hip1	huntingtin interacting protein 1	1.06
152	Prdm1	PR domain containing 1. with ZNF domain	1.16
153	Prkar2a	protein kinase. cAMP dependent regulatory. type II alpha	0.97
154	Itga4	integrin alpha 4	1.36
155	Zyx	zyxin	1.14
156	Baiap3	BAI1-associated protein 3	1.16
157	Atp2b4	ATPase. Ca++ transporting. plasma membrane 4	1.25

158	Rnf138	ring finger protein 138	0.95
159	Cish	cytokine inducible SH2-containing protein	1.04
160	Gna15	guanine nucleotide binding protein. alpha 15	1.4
161	Alcam	activated leukocyte cell adhesion molecule	1.01
162	Golim4	golgi integral membrane protein 4	0.89
163	Gimap7	GTPase. IMAP family member 7	0.92
164	Arsb	arylsulfatase B	0.99
165	Gpr160	G protein-coupled receptor 160	1.04
166	Smad3	MAD homolog 3 (Drosophila)	1.26
167	Lax1	lymphocyte transmembrane adaptor 1	0.94
168	Gbp3	guanylate binding protein 3	0.82
169	Coro2a	coronin. actin binding protein 2A	0.99
170	Cldn12	claudin 12	0.89
171	Ptpn13	protein tyrosine phosphatase. non-receptor type 13	1
172	Gramd1b	GRAM domain containing 1B	0.97
173	Nfatc2	nuclear factor of activated T-cells. cytoplasmic. calcineuri	0.99
174	Rgs3	regulator of G-protein signaling 3	0.98
175	Dennd4a	DENN/MADD domain containing 4A	1.52
176	Dapk2	death-associated protein kinase 2	1.22
177	Sytl3	synaptotagmin-like 3	1.01
178	Dennd4c	DENN/MADD domain containing 4C	0.9
179	Snx10	sorting nexin 10	0.96
180	Prf1	perforin 1 (pore forming protein)	1.25
181	Ggh	gamma-glutamyl hydrolase	1.06
182	Odc1	ornithine decarboxylase. structural 1	0.9
183	6330512M04Rik	RIKEN cDNA 6330512M04 gene	1.18
184	Cldnd1	claudin domain containing 1	1
185	Sntb2	syntrophin. basic 2	0.94
186	Cpd	carboxypeptidase D	1
187	Stard10	START domain containing 10	1.26
188	Chpt1	choline phosphotransferase 1	0.87
189	Itgal	integrin alpha L	1.17
190	2010002N04Rik	RIKEN cDNA 2010002N04 gene	0.88
191	Rhoc	ras homolog gene family. member C	0.92
192	Pla2g16	phospholipase A2. group XVI	0.84
193	Vim	vimentin	1.09
194	Mcart6	mitochondrial carrier triple repeat 6	0.74
195	AW112010	expressed sequence AW112010	0.76
196	1110067D22Rik	RIKEN cDNA 1110067D22 gene	0.91
197	Wdr95	WD40 repeat domain 95	0.99
198	Zbtb7b	zinc finger and BTB domain containing 7B	1.14
199	Jdp2	Jun dimerization protein 2	0.83
200	Serpinb6b	serine (or cysteine) peptidase inhibitor. clade B. member 6b	0.83
201	Pou6f1	POU domain. class 6. transcription factor 1	0.95
202	Mx1	myxovirus (influenza virus) resistance 1	0.78
203	Sidt1	SID1 transmembrane family. member 1	0.86
204	Prr13	proline rich 13	0.89
205	Pou2f2	POU domain. class 2. transcription factor 2	0.81
206	Il7r	interleukin 7 receptor	1.01
207	Ncald	neurocalcin delta	1.05
208	Mapkapk3	mitogen-activated protein kinase-activated protein kinase 3	0.73
209	Prkce	protein kinase C. epsilon	0.94
210	Nfil3	nuclear factor. interleukin 3. regulated	0.92
211	Stk39	serine/threonine kinase 39. STE20/SPS1 homolog (yeast)	0.85
212	Dusp5	dual specificity phosphatase 5	1.14

213	Dgkh	diacylglycerol kinase. eta	0.92
214	Emp1	epithelial membrane protein 1	1.14
215	Ctsw	cathepsin W	0.76
216	Bcl2	B-cell leukemia/lymphoma 2	0.61
217	Ctla4	cytotoxic T-lymphocyte-associated protein 4	0.88
218	Fam129b	family with sequence similarity 129. member B	0.88
219	Slc39a4	solute carrier family 39 (zinc transporter). member 4	0.78
220	Tmem51	transmembrane protein 51	0.81
221	Gvin1	GTPase. very large interferon inducible 1	0.78
222	Zcchc18	zinc finger. CCHC domain containing 18	0.79
223	Nod1	nucleotide-binding oligomerization domain containing 1	0.89
224	4930486L24Rik	RIKEN cDNA 4930486L24 gene	0.81
225	Rap1gap2	RAP1 GTPase activating protein 2	1.37
226	Cd160	CD160 antigen	0.85
227	Stx11	syntaxin 11	0.85
228	Inpp5d	inositol polyphosphate-5-phosphatase D	0.9
229	Map3k8	mitogen-activated protein kinase kinase kinase 8	0.76
230	Hspa1a	heat shock protein 1A	0.99
231	Mettl7a1	methyltransferase like 7A1	0.88
232	Dnaja4	DnaJ (Hsp40) homolog. subfamily A. member 4	0.8
233	Cysltr2	cysteinyl leukotriene receptor 2	0.78
234	Unc119	unc-119 homolog (C. elegans)	0.88
235	Dtx4	deltex 4 homolog (Drosophila)	0.77
236	Vmp1	vacuole membrane protein 1	0.61
237	Cd82	CD82 antigen	0.74
238	Myadm	myeloid-associated differentiation marker	0.83
239	Tmbim1	transmembrane BAX inhibitor motif containing 1	0.75
240	Sh2d2a	SH2 domain protein 2A	0.75
241	Lamc1	laminin. gamma 1	0.86
242	Tnfrsf25	tumor necrosis factor receptor superfamily. member 25	0.79
243	Lgals3	lectin. galactose binding. soluble 3	1.15
244	Epas1	endothelial PAS domain protein 1	0.85
245	Nbeal2	neurobeachin-like 2	1.01
246	Rnf157	ring finger protein 157	0.62
247	Pcgf2	polycomb group ring finger 2	0.86
248	Naip2	NLR family. apoptosis inhibitory protein 2	0.94
249	BC005685	cDNA sequence BC005685	0.48
250	Dstn	destrin	0.77
251	Rap2a	RAS related protein 2a	0.8
252	Racgap1	Rac GTPase-activating protein 1	0.8
253	Il10rb	interleukin 10 receptor. beta	0.94
254	Usp18	ubiquitin specific peptidase 18	0.41
255	Ust	uronyl-2-sulfotransferase	0.76
256	Pvt1	plasmacytoma variant translocation 1	0.76
257	Klrb1f	killer cell lectin-like receptor subfamily B member 1F	1.07
258	Slc4a7	solute carrier family 4. sodium bicarbonate cotransporter. m	0.9
259	Hcst	hematopoietic cell signal transducer	0.72
260	Gbp7	guanylate binding protein 7	0.49
261	Ptpn4	protein tyrosine phosphatase. non-receptor type 4	1.03
262	Ifit3	interferon-induced protein with tetratricopeptide repeats 3	0.21
263	Ppp3cc	protein phosphatase 3. catalytic subunit. gamma isoform	0.71
264	B4galt5	UDP-Gal:betaGlcNAc beta 1.4-galactosyltransferase. polypepti	0.82
265	Pfkp	phosphofructokinase. platelet	0.92
266	F2rl2	coagulation factor II (thrombin) receptor-like 2	1.07
267	Scpep1	serine carboxypeptidase 1	0.78

268	Sla	src-like adaptor	0.79
269	Glrx	glutaredoxin	0.71
270	Abi2	abl-interactor 2	0.63
271	Icos	inducible T-cell co-stimulator	0.66
272	Gprin3	GPRIN family member 3	0.81
273	Casp3	caspase 3	0.66
274	Wwp1	WW domain containing E3 ubiquitin protein ligase 1	0.89
275	Ifitm3	interferon induced transmembrane protein 3	0.28
276	Dok2	docking protein 2	0.83
277	Mapk11	mitogen-activated protein kinase 11	0.71
278	BC016495	cDNA sequence BC016495	0.58
279	Plekhg3	pleckstrin homology domain containing. family G (with RhoGef	0.72
280	Pde2a	phosphodiesterase 2A. cGMP-stimulated	0.76
281	Golm1	golgi membrane protein 1	0.65
282	Lpin2	lipin 2	0.71
283	Tmem159	transmembrane protein 159	0.64
284	Galnt3	UDP-N-acetyl-alpha-D-galactosamine:polypeptide N-acetylgalac	0.87
285	Ptpn3	protein tyrosine phosphatase. non-receptor type 3	0.76
286	Eea1	early endosome antigen 1	0.91
287	Gpc1	glypican 1	0.74
288	Gfod1	glucose-fructose oxidoreductase domain containing 1	0.81
289	Chic1	cysteine-rich hydrophobic domain 1	0.75
290	Rab8b	RAB8B. member RAS oncogene family	0.61
291	Crybg3	beta-gamma crystallin domain containing 3	1.03
292	Sema4f	sema domain. immunoglobulin domain (Ig). TM domain. and shor	0.59
293	Mthfs	5. 10-methenyltetrahydrofolate synthetase	0.55
294	Lass4	LAG1 homolog. ceramide synthase 4	0.72
295	Pelo	pelota homolog (Drosophila)	0.75
296	Trip4	thyroid hormone receptor interactor 4	0.77
297	Susd3	sushi domain containing 3	0.68
298	Lpxn	leupaxin	0.56
299	A930038C07Rik	RIKEN cDNA A930038C07 gene	0.65
300	Hmgb2	high mobility group box 2	1.04
301	Rnf216	ring finger protein 216	1.01
302	Stat4	signal transducer and activator of transcription 4	0.72
303	Plac8	placenta-specific 8	-0.27
304	Acpl2	acid phosphatase-like 2	0.6
305	Adam8	a disintegrin and metallopeptidase domain 8	0.69
306	Mtmr7	myotubularin related protein 7	0.71
307	Lmna	lamin A	0.69
308	Ssh1	slingshot homolog 1 (Drosophila)	0.69
309	Ifngr1	interferon gamma receptor 1	0.83
310	Smyd1	SET and MYND domain containing 1	0.68
311	C230081A13Rik	RIKEN cDNA C230081A13 gene	0.64
312	Il15	interleukin 15	0.79
313	Grina	glutamate receptor. ionotropic. N-methyl D-aspartate-associa	0.65
314	Rptor	regulatory associated protein of MTOR. complex 1	0.73
315	Lxn	latexin	0.62
316	Atxn1	ataxin 1	0.78
317	Rab19	RAB19. member RAS oncogene family	0.55
318	Apobec2	apolipoprotein B mRNA editing enzyme. catalytic polypeptide	0.55
319	Apobr	apolipoprotein B receptor	0.73
320	Ikzf3	IKAROS family zinc finger 3	0.7
321	Usp48	ubiquitin specific peptidase 48	0.82
322	Serpinb6a	serine (or cysteine) peptidase inhibitor. clade B. member 6a	0.61

323	Socs2	suppressor of cytokine signaling 2	0.72
324	Ehbp1l1	EH domain binding protein 1-like 1	0.66
325	Ctsd	cathepsin D	0.72
326	Ppp1r12a	protein phosphatase 1. regulatory (inhibitor) subunit 12A	0.85
327	Slc2a3	solute carrier family 2 (facilitated glucose transporter). m	0.6
328	Carhsp1	calcium regulated heat stable protein 1	0.71
329	Lcn4	lipocalin 4	0.38
330	Errfi1	ERBB receptor feedback inhibitor 1	0.95
331	Oplah	5-oxoprolinase (ATP-hydrolysing)	0.47
332	Snx14	sorting nexin 14	0.64
333	Frmd4b	FERM domain containing 4B	0.31
334	Ptger4	prostaglandin E receptor 4 (subtype EP4)	0.46
335	Ncf4	neutrophil cytosolic factor 4	0.54
336	Pik3r5	phosphoinositide-3-kinase. regulatory subunit 5. p101	0.73
337	Il15ra	interleukin 15 receptor. alpha chain	0.65
338	Tef	thyrotroph embryonic factor	0.74
339	Hip1r	huntingtin interacting protein 1 related	0.67
340	Cd7	CD7 antigen	0.65
341	Reck	reversion-inducing-cysteine-rich protein with kazal motifs	0.51
342	Tmem37	transmembrane protein 37	0.66
343	Ccl9	chemokine (C-C motif) ligand 9	1.1
344	L1cam	L1 cell adhesion molecule	0.77
345	Fam110a	family with sequence similarity 110. member A	0.55
346	Ddx28	DEAD (Asp-Glu-Ala-Asp) box polypeptide 28	0.48
347	Atp10a	ATPase. class V. type 10A	0.72
348	Slco3a1	solute carrier organic anion transporter family. member 3a1	0.66
349	3830406C13Rik	RIKEN cDNA 3830406C13 gene	0.38
350	Bbs12	Bardet-Biedl syndrome 12 (human)	0.53
351	Ksr1	kinase suppressor of ras 1	0.64
352	Atp8b4	ATPase. class I. type 8B. member 4	0.74
353	Stap1	signal transducing adaptor family member 1	0.31
354	Abcb1b	ATP-binding cassette. sub-family B (MDR/TAP). member 1B	0.28
355	Cyfip1	cytoplasmic FMR1 interacting protein 1	0.89
356	Atp2b1	ATPase. Ca++ transporting. plasma membrane 1	0.77
357	Sorl1	sortilin-related receptor. LDLR class A repeats-containing	0.48
358	Vopp1	vesicular. overexpressed in cancer. prosurvival protein 1	0.42
359	Cpne3	copine III	0.79
360	Epsti1	epithelial stromal interaction 1 (breast)	0.78
361	Tm6sf1	transmembrane 6 superfamily member 1	0.5
362	Lcp1	lymphocyte cytosolic protein 1	0.6
363	Pik3cg	phosphoinositide-3-kinase. catalytic. gamma polypeptide	0.49
364	Rhoq	ras homolog gene family. member Q	0.57
365	Pvr	poliovirus receptor-related 2	0.69
366	Man2a1	mannosidase 2. alpha 1	0.69
367	Katnb1	katanin p80 (WD40-containing) subunit B 1	0.88
368	Gpr15	G protein-coupled receptor 15	0.73
369	2310044G17Rik	RIKEN cDNA 2310044G17 gene	0.61
370	Cd80	CD80 antigen	0.87
371	Cd28	CD28 antigen	0.76
372	Batf	basic leucine zipper transcription factor. ATF-like	0.49
373	D19Ertd737e	DNA segment. Chr 19. ERATO Doi 737. expressed	0.69
374	Gsto1	glutathione S-transferase omega 1	0.39
375	Bspry	B-box and SPRY domain containing	0.71
376	Bag3	BCL2-associated athanogene 3	0.63
377	Atp6v0d2	ATPase. H+ transporting. lysosomal V0 subunit D2	0.64

378	Nptn	neuroplastin	0.7
379	2010111I01Rik	RIKEN cDNA 2010111I01 gene	0.48
380	Gpr68	G protein-coupled receptor 68	0.49
381	Ifi203	interferon activated gene 203	0.42
382	Ifitm1	interferon induced transmembrane protein 1	0.67
383	Mmgt2	membrane magnesium transporter 2	0.5
384	Ube2g2	ubiquitin-conjugating enzyme E2G 2	0.71
385	Insl6	insulin-like 6	0.63
386	Pdcd1lg2	programmed cell death 1 ligand 2	0.45
387	Pmaip1	phorbol-12-myristate-13-acetate-induced protein 1	0.64
388	Lime1	Lck interacting transmembrane adaptor 1	0.5
389	Ptger2	prostaglandin E receptor 2 (subtype EP2)	0.52
390	Enpp1	ectonucleotide pyrophosphatase/phosphodiesterase 1	0.58
391	Litaf	LPS-induced TN factor	0.56
392	Arhgap18	Rho GTPase activating protein 18	0.63
393	Pak6	p21 protein (Cdc42/Rac)-activated kinase 6	0.69
394	Impact	imprinted and ancient	0.6
395	Clic4	chloride intracellular channel 4 (mitochondrial)	0.52
396	Edaradd	EDAR (ectodysplasin-A receptor)-associated death domain	0.71
397	Sephs2	selenophosphate synthetase 2	0.64
398	Slamf1	signaling lymphocytic activation molecule family member 1	0.41
399	Zfpm1	zinc finger protein. multitype 1	0.62
400	Ttc7b	tetratricopeptide repeat domain 7B	0.78
401	Sfi1	Sfi1 homolog. spindle assembly associated (yeast)	0.57
402	Wee1	WEE 1 homolog 1 (S. pombe)	0.54
403	Tnfrsf9	tumor necrosis factor receptor superfamily. member 9	0.14
404	2610029I01Rik	RIKEN cDNA 2610029I01 gene	0.4
405	Bmpr1a	bone morphogenetic protein receptor. type 1A	0.49
406	Ap1s2	adaptor-related protein complex 1. sigma 2 subunit	0.19
407	Thtpa	thiamine triphosphatase	0.59
408	Ctse	cathepsin E	0.11
409	Pttg1	pituitary tumor-transforming gene 1	0.6
410	Bivm	basic. immunoglobulin-like variable motif containing	0.53
411	Ank	progressive ankylosis	0.59
412	Rpa2	replication protein A2	0.61
413	Plxnc1	plexin C1	0.6
414	Sos2	son of sevenless homolog 2 (Drosophila)	0.66
415	Cxcr4	chemokine (C-X-C motif) receptor 4	0.53
416	Ret	ret proto-oncogene	0.66
417	Atp11a	ATPase. class VI. type 11A	0.47
418	Lysmd2	LysM. putative peptidoglycan-binding. domain containing 2	0.43
419	1110058L19Rik	RIKEN cDNA 1110058L19 gene	0.5
420	As3mt	arsenic (+3 oxidation state) methyltransferase	0.63
421	Cd86	CD86 antigen	0.4
422	Gabarapl2	gamma-aminobutyric acid (GABA) A receptor-associated protein	0.78
423	Myo5a	myosin VA	0.59
424	Anxa6	annexin A6	0.78
425	Fnip2	folliculin interacting protein 2	0.53
426	Tmem163	transmembrane protein 163	0.92
427	Vps54	vacuolar protein sorting 54 (yeast)	0.3
428	Ifi30	interferon gamma inducible protein 30	0.57
429	Tmem140	transmembrane protein 140	0.39
430	Grap	GRB2-related adaptor protein	0.54
431	Nmnat2	nicotinamide nucleotide adenylyltransferase 2	0.48
432	Ntng2	netrin G2	0.59

433	Lmnb1	lamin B1	0.54
434	Asrgl1	asparaginase like 1	0.5
435	St6galnac4	ST6 (alpha-N-acetyl-neuraminyl-2.3-beta-galactosyl-1.3)-N-ac	0.35
436	Ppp1r11	protein phosphatase 1. regulatory (inhibitor) subunit 11	0.61
437	Pld2	phospholipase D2	0.62
438	Lair1	leukocyte-associated Ig-like receptor 1	0.56
439	Mettl21d	methyltransferase like 21D	0.16
440	Mrpl52	mitochondrial ribosomal protein L52	0.2
441	S1pr2	sphingosine-1-phosphate receptor 2	0.45
442	Gm14005	predicted gene 14005	0.54
443	Gdf11	growth differentiation factor 11	0.79
444	Pde7a	phosphodiesterase 7A	0.65
445	Cd47	CD47 antigen (Rh-related antigen. integrin-associated signal	0.46
446	Itgb2	integrin beta 2	0.55
447	sept-11	septin 11	0.44
448	Zfp3	zinc finger protein 3	0.59
449	Sypl	synaptophysin-like protein	0.31
450	Plekhf1	pleckstrin homology domain containing. family F (with FYVE d	0.56
451	Gas7	growth arrest specific 7	0.74
452	Cd38	CD38 antigen	0.7
453	Tyrobp	TYRO protein tyrosine kinase binding protein	0.52
454	2410022L05Rik	RIKEN cDNA 2410022L05 gene	0.52
455	Nr1d2	nuclear receptor subfamily 1. group D. member 2	0.65
456	Arl4c	ADP-ribosylation factor-like 4C	0.63
457	Samhd1	SAM domain and HD domain. 1	0.5
458	Fbxw8	F-box and WD-40 domain protein 8	0.57
459	Dse	dermatan sulfate epimerase	0.4
460	Nqo2	NAD(P)H dehydrogenase. quinone 2	0.58
461	Plekhm3	pleckstrin homology domain containing. family M. member 3	0.62
462	Gimap4	GTPase. IMAP family member 4	0.54
463	H2-Q8	histocompatibility 2. Q region locus 8	0.63
464	Myo3b	myosin IIIB	0.81
465	Klf10	Kruppel-like factor 10	0.54
466	Kbtbd11	kelch repeat and BTB (POZ) domain containing 11	0.5
467	Kdelc1	KDEL (Lys-Asp-Glu-Leu) containing 1	0.36
468	Nedd4	neural precursor cell expressed. developmentally down-regula	0.56
469	Fam26f	family with sequence similarity 26. member F	0.15
470	Med7	mediator complex subunit 7	0.32
471	Ptpre	protein tyrosine phosphatase. receptor type. E	0.61
472	Cpox	coproporphyrinogen oxidase	0.3
473	Fpgt	fucose-1-phosphate guanylyltransferase	0.21
474	Rbpj	recombination signal binding protein for immunoglobulin kapp	0.31
475	Jak2	Janus kinase 2	0.32
476	Mlkl	mixed lineage kinase domain-like	0.63
477	Tnfrsf18	tumor necrosis factor receptor superfamily. member 18	0.28
478	Fcho2	FCH domain only 2	0.6
479	Cyba	cytochrome b-245. alpha polypeptide	0.29
480	2700089E24Rik	RIKEN cDNA 2700089E24 gene	0.32
481	Cdkn1a	cyclin-dependent kinase inhibitor 1A (P21)	0.51
482	Slc15a3	solute carrier family 15. member 3	0.5
483	Sema4a	sema domain. immunoglobulin domain (Ig). transmembrane domai	0.47
484	Iqgap2	IQ motif containing GTPase activating protein 2	0.71
485	Rnf19b	ring finger protein 19B	0.5
486	Pde8a	phosphodiesterase 8A	0.55
487	Dynlt3	dynein light chain Tctex-type 3	0.04

488	Rdm1	RAD52 motif 1	0.42
489	Hk2	hexokinase 2	0.44
490	Rabgap1l	RAB GTPase activating protein 1-like	0.53
491	Tulp4	tubby like protein 4	0.41
492	Trim30d	tripartite motif-containing 30D	0.22
493	Nsmaf	neutral sphingomyelinase (N-SMase) activation associated fac	0.54
494	Rcn1	reticulocalbin 1	0.36
495	Dopey2	dopey family member 2	0.43
496	Tmtc4	transmembrane and tetratricopeptide repeat containing 4	0.51
497	Ifnar2	interferon (alpha and beta) receptor 2	0.46
498	1110001A16Rik	RIKEN cDNA 1110001A16 gene	0.46
499	Wdfy1	WD repeat and FYVE domain containing 1	0.44
500	Ubc	ubiquitin A-52 residue ribosomal protein fusion product 1	0.6
501	Itgam	integrin alpha M	0.63
502	Ebpl	emopamil binding protein-like	0.42
503	Dyrk3	dual-specificity tyrosine-(Y)-phosphorylation regulated kina	0.44
504	Abhd14a	abhydrolase domain containing 14A	0.46
505	H1f0	H1 histone family. member 0	0.6
506	Scrn3	secernin 3	0.53
507	Plcd1	phospholipase C. delta 1	0.69
508	Cyb5r4	cytochrome b5 reductase 4	0.53
509	Txndc5	thioredoxin domain containing 5	0.62
510	Fyn	Fyn proto-oncogene	0.64
511	Nek6	NIMA (never in mitosis gene a)-related expressed kinase 6	0.44
512	Dcbld2	discoidin. CUB and LCCL domain containing 2	0.45
513	St3gal4	ST3 beta-galactoside alpha-2.3-sialyltransferase 4	0.57
514	Havcr2	hepatitis A virus cellular receptor 2	0.43
515	Phf21b	PHD finger protein 21B	0.53
516	Pdcd1	programmed cell death 1	0.82
517	Vkorc1	vitamin K epoxide reductase complex. subunit 1	0.29
518	Irak2	interleukin-1 receptor-associated kinase 2	0.46
519	Prkcz	protein kinase C. zeta	0.57
520	Zfp69	zinc finger protein 84	0.44
521	Gapdh	glyceraldehyde-3-phosphate dehydrogenase	0.7
522	Parp8	poly (ADP-ribose) polymerase family. member 8	0.5
523	Rab39b	RAB39B. member RAS oncogene family	0.46
524	Krtcap2	keratinocyte associated protein 2	0.3
525	Aplp2	amyloid beta (A4) precursor-like protein 2	0.7
526	Etfb	electron transferring flavoprotein. beta polypeptide	0.48
527	Smap1	stromal membrane-associated protein 1	0.69
528	H2-Q7	histocompatibility 2. Q region locus 7	0.52
529	Map2k3	mitogen-activated protein kinase kinase 3	0.34
530	Fam92a	family with sequence similarity 92. member A	0.39
531	Nek7	NIMA (never in mitosis gene a)-related expressed kinase 7	0.44
532	Tbc1d12	TBC1D12: TBC1 domain family. member 12	0.46
533	Nckap1	NCK-associated protein 1	0.4
534	Nudt4	nudix (nucleoside diphosphate linked moiety X)-type motif 4	0.49
535	Mki67	antigen identified by monoclonal antibody Ki 67	0.37
536	1700049G17Rik	RIKEN cDNA 1700049G17 gene	0.38
537	Igf2r	insulin-like growth factor 2 receptor	0.64
538	BC004004	cDNA sequence BC004004	0.36
539	Cd2	CD2 antigen	0.49
540	Tbkbp1	TBK1 binding protein 1	0.58
541	Ppp1r3e	protein phosphatase 1. regulatory (inhibitor) subunit 3E	0.46
542	Fam49a	family with sequence similarity 49. member A	0.59

543	Kdm4d	lysine (K)-specific demethylase 4D	0.52
544	Fam19a3	family with sequence similarity 19. member A3	0.61
545	Cmc1	COX assembly mitochondrial protein homolog (S. cerevisiae)	0.37
546	Slc16a6	solute carrier family 16 (monocarboxylic acid transporters).	0.46
547	Rhbdf2	rhomboid 5 homolog 2 (Drosophila)	0.5
548	Tk1	thymidine kinase 1	0.63
549	Zbtb42	zinc finger and BTB domain containing 42	0.46
550	Crot	carnitine O-octanoyltransferase	0.44
551	Armc7	armadillo repeat containing 7	0.54
552	Abhd14b	abhydrolase domain containing 14b	0.32
553	Traf1	TNF receptor-associated factor 1	0.53
554	Capg	capping protein (actin filament). gelsolin-like	0.56
555	Polk	polymerase (DNA directed). kappa	0.37
556	Cd40lg	CD40 ligand	0.13
557	Prdx4	peroxiredoxin 4	0.48
558	Pdgfb	platelet derived growth factor. B polypeptide	0.68
559	Cnrip1	cannabinoid receptor interacting protein 1	0.64
560	Clstn1	calsyntenin 1	0.49
561	Dnajc15	DnaJ (Hsp40) homolog. subfamily C. member 15	0.37
562	Plagl1	pleiomorphic adenoma gene-like 1	0.37
563	Slc3a2	solute carrier family 3 (activators of dibasic and neutral a	0.28
564	Tdrd7	tudor domain containing 7	0.34
565	Osbpl5	oxysterol binding protein-like 5	0.51
566	Thap3	THAP domain containing. apoptosis associated protein 3	0.49
567	Tmed3	transmembrane emp24 domain containing 3	0.21
568	Tesk1	testis specific protein kinase 1	0.5
569	Crip1	cysteine-rich protein 1 (intestinal)	0.49
570	Slc41a2	solute carrier family 41. member 2	0.52
571	Cldnd2	claudin domain containing 2	0.52
572	Tyk2	tyrosine kinase 2	0.34
573	Impa2	inositol (myo)-1(or 4)-monophosphatase 2	0.41
574	Cpeb2	cytoplasmic polyadenylation element binding protein 2	0.49
575	Laptm5	lysosomal-associated protein transmembrane 5	0.54
576	Calm2	calmodulin 1	0.8
577	Rara	retinoic acid receptor. alpha	0.56
578	Hgsnat	heparan-alpha-glucosaminide N-acetyltransferase	0.49
579	Selm	selenoprotein M	0.4
580	Angptl4	angiopoietin-like 4	0.42
581	Rab23	RAB23. member RAS oncogene family	0.33
582	Mapk12	mitogen-activated protein kinase 12	0.51
583	Cks2	CDC28 protein kinase regulatory subunit 2	0.01
584	Plekhb2	pleckstrin homology domain containing. family B (evectins) m	0.43
585	Asap2	ArfGAP with SH3 domain. ankyrin repeat and PH domain 2	0.54
586	Prune	prune homolog (Drosophila)	0.53
587	2310046K01Rik	RIKEN cDNA 2310046K01 gene	0.39
588	S100a11	S100 calcium binding protein A11 (calgizzarin)	0.79
589	Txnrd1	thioredoxin reductase 1	0.3
590	Tmem180	transmembrane protein 180	0.41
591	Ehd3	EH-domain containing 3	0.6
592	Ubxn2b	UBX domain protein 2B	0.34
593	Rbm47	RNA binding motif protein 47	0.46
594	Zbtb38	zinc finger and BTB domain containing 38	0.54
595	Vcl	vinculin	0.47
596	Crb3	crumbs homolog 3 (Drosophila)	0.46
597	Chst2	carbohydrate sulfotransferase 2	0.42

598	Fbxw7	F-box and WD-40 domain protein 7	0.35
599	2210411K11Rik	RIKEN cDNA 2210411K11 gene	0.41
600	Samsn1	SAM domain. SH3 domain and nuclear localization signals. 1	0.29
601	Gpr132	G protein-coupled receptor 132	0.68
602	Dnajb6	DnaJ (Hsp40) homolog. subfamily B. member 6	0.39
603	Obfc2a	oligonucleotide/oligosaccharide-binding fold containing 2A	0.51
604	Ralgds	ral guanine nucleotide dissociation stimulator	0.4
605	9630033F20Rik	RIKEN cDNA 9630033F20 gene	0.36
606	Atxn2l	ataxin 2-like	0.52
607	Tnfaip3	tumor necrosis factor. alpha-induced protein 3	0.68
608	Cblb	Casitas B-lineage lymphoma b	0.41
609	Tgoln1	trans-golgi network protein	0.52
610	Aars	alanyl-tRNA synthetase	0.38
611	Sfxn1	sideroflexin 1	0.1
612	Ostf1	osteoclast stimulating factor 1	0.42
613	Ei24	etoposide induced 2.4 mRNA	0.17
614	Igk	immunoglobulin kappa chain complex	0.39
615	Cd8a	CD8 antigen. alpha chain	0.38
616	B3gnt5	UDP-GlcNAc:betaGal beta-1.3-N-acetylglucosaminyltransferase	0.32
617	Wbp1	WW domain binding protein 1	0.38
618	Cecr5	cat eye syndrome chromosome region. candidate 5 homolog (hum	0.43
619	Ubash3a	ubiquitin associated and SH3 domain containing. A	0.4
620	Tg	thyroglobulin	0.37
621	Zfp821	zinc finger protein 821	0.39
622	Pear1	platelet endothelial aggregation receptor 1	0.45
623	Hint2	histidine triad nucleotide binding protein 2	0.26
624	Ifit2	interferon-induced protein with tetratricopeptide repeats 2	0.07
625	Calhm2	calcium homeostasis modulator 2	0.39
626	Nfat5	nuclear factor of activated T-cells 5	0.36
627	Whsc1	Wolf-Hirschhorn syndrome candidate 1 (human)	0.39
628	Dhrs7	dehydrogenase/reductase (SDR family) member 7	0.2
629	Tesc	tescalcin	0.29
630	Blcap	bladder cancer associated protein homolog (human)	0.3
631	Mfsd6	major facilitator superfamily domain containing 6	0.34
632	Fryl	furry homolog-like (Drosophila)	0.38
633	Rhof	ras homolog gene family. member f	0.26
634	Padi2	peptidyl arginine deiminase. type II	0.22
635	Nr1d1	nuclear receptor subfamily 1. group D. member 1	0.5
636	Rabep2	rabaptin. RAB GTPase binding effector protein 2	0.47
637	Ubash3b	ubiquitin associated and SH3 domain containing. B	0.28
638	Mns1	meiosis-specific nuclear structural protein 1	0.38
639	D8Ertd82e	DNA segment. Chr 8. ERATO Doi 82. expressed	0.32
640	Ptpn22	protein tyrosine phosphatase. non-receptor type 22 (lymphoid	0.53
641	Phf1	PHD finger protein 1	0.55
642	Ccdc107	coiled-coil domain containing 107	0.28
643	Tnfsf10	tumor necrosis factor (ligand) superfamily. member 10	0.13
644	Syngr2	synaptogyrin 2	0.42
645	AY036118	cDNA sequence AY036118	0.89
646	Sec24d	Sec24 related gene family. member D (S. cerevisiae)	0.44
647	Eif4ebp1	eukaryotic translation initiation factor 4E binding protein	0.33
648	Cd68	CD68 antigen	0.57
649	Rinl	Ras and Rab interactor-like	0.41
650	Med12l	mediator of RNA polymerase II transcription. subunit 12 homo	0.36
651	Surf1	surfeit gene 1	0.31
652	Frrs1	ferric-chelate reductase 1	0.24

653	1810027O10Rik	RIKEN cDNA 1810027O10 gene	0.23
654	Gem	GTP binding protein (gene overexpressed in skeletal muscle)	0.42
655	Mrpl44	mitochondrial ribosomal protein L44	0.24
656	Gpr137b-ps	G protein-coupled receptor 137B. pseudogene	0.43
657	1110008P14Rik	RIKEN cDNA 1110008P14 gene	0.4
658	Haus4	HAUS augmin-like complex. subunit 4	0.41
659	Mthfr	5.10-methylenetetrahydrofolate reductase	0.4
660	Ide	insulin degrading enzyme	0.46
661	Glipr2	GLI pathogenesis-related 2	0.36
662	Gpr155	G protein-coupled receptor 155	0.61
663	Mknk2	MAP kinase-interacting serine/threonine kinase 2	0.53
664	Sema4d	sema domain. immunoglobulin domain (Ig). transmembrane domai	0.43
665	Ly96	lymphocyte antigen 96	0.21
666	Rttn	rotatin	0.23
667	Rgs2	regulator of G-protein signaling 2	0.66
668	Serinc3	serine incorporator 3	0.58
669	Pip4k2b	phosphatidylinositol-5-phosphate 4-kinase. type II. beta	0.33
670	Alox8	arachidonate 8-lipoxygenase	0.57
671	Pim1	proviral integration site 1	0.69
672	Slc9a9	solute carrier family 9 (sodium/hydrogen exchanger). member	0.57
673	Naip5	NLR family. apoptosis inhibitory protein 5	0.48
674	Tmem8	transmembrane protein 8 (five membrane-spanning domains)	0.41
675	H2afz	H2A histone family. member Z	0.49
676	Abi3	ABI gene family. member 3	0.19
677	Grk5	G protein-coupled receptor kinase 5	0.35
678	Pycard	PYD and CARD domain containing	0.22
679	Slc35f2	solute carrier family 35. member F2	0.1
680	Gas2l1	growth arrest-specific 2 like 1	0.4
681	Tbl2	transducin (beta)-like 2	0.52
682	Cyp4v3	cytochrome P450. family 4. subfamily v. polypeptide 3	0.2
683	Ryk	receptor-like tyrosine kinase	0.56
684	Alad	aminolevulinate. delta-. dehydratase	0.39
685	Nr4a2	nuclear receptor subfamily 4. group A. member 2	0.51
686	Bcl6	B-cell leukemia/lymphoma 6	0.2
687	Hps4	Hermansky-Pudlak syndrome 4 homolog (human)	0.21
688	Marveld2	MARVEL (membrane-associating) domain containing 2	0.34
689	5330426P16Rik	RIKEN cDNA 5330426P16 gene	0.43
690	Pigc	phosphatidylinositol glycan anchor biosynthesis. class C	0.23
691	Chac1	ChaC. cation transport regulator-like 1 (E. coli)	0.4
692	Pxmp3	peroxisomal membrane protein 3	-0.05
693	Nradd	neurotrophin receptor associated death domain	0.5
694	Cd22	CD22 antigen	0.63
695	Pbx3	pre B-cell leukemia transcription factor 3	0.49
696	Dym	dymeclin	0.39
697	Glipr1	GLI pathogenesis-related 1 (glioma)	0.24
698	Rnf39	ring finger protein 39	0.41
699	Pygl	liver glycogen phosphorylase	0.43
700	Gpr174	G protein-coupled receptor 174	0.09
701	Tnfsf8	tumor necrosis factor (ligand) superfamily. member 8	-0.06
702	Ifitm2	interferon induced transmembrane protein 2	0.48
703	Pias3	protein inhibitor of activated STAT 3	0.45
704	Gla	galactosidase. alpha	0.28
705	Mid1ip1	Mid1 interacting protein 1 (gastrulation specific G12-like (	0.31
706	Fosb	FBJ osteosarcoma oncogene B	0.81
707	Irf8	interferon regulatory factor 8	0.33

708	Prkca	protein kinase C. alpha	0.46
709	Aen	apoptosis enhancing nuclease	0.16
710	D16Ertd472e	DNA segment. Chr 16. ERATO Doi 472. expressed	0.07
711	Tmem55a	transmembrane protein 55A	0.2
712	Zdhhc18	zinc finger. DHHC domain containing 18	0.4
713	Zfp937	zinc finger protein 937	0.51
714	Ppp2r2c	protein phosphatase 2 (formerly 2A). regulatory subunit B (P	0.39
715	Wipf1	WAS/WASL interacting protein family. member 1	0.41
716	Sorbs1	sorbin and SH3 domain containing 1	0.44

SUPPLEMENTARY TABLE S11. List of down-regulated genes with their ranking in the meta-analysis performed as described in the Methods. The first column is giving the gene rank according to the gene rank product (the largest FC across studies are associated with the smallest Rank Product). The last column is the average expression levels under two conditions (Memory and Naive) given as a log2 fold change. List of up-regulated genes with their ranking in the meta-analysis performed as described in the methods.

Rank	Name	Description	log2FC(Mem vs Na)
1	Ccr9	chemokine (C-C motif) receptor 9	-4.05
2	Atp1b1	ATPase, Na+/K+ transporting, beta 1 polypeptide	-3.89
3	St6gal1	beta galactoside alpha 2,6 sialyltransferase 1	-3.48
4	Igfbp4	insulin-like growth factor binding protein 4	-3.43
5	Ifngr2	interferon gamma receptor 2	-2.69
6	Ephx1	epoxide hydrolase 1, microsomal	-2.94
7	Itgae	integrin alpha E, epithelial-associated	-2.7
8	Cnn3	calponin 3, acidic	-2.6
9	Xkrx	X Kell blood group precursor related X linked	-2.41
10	Ift80	intraflagellar transport 80 homolog (Chlamydomonas)	-2.46
11	Ikzf2	IKAROS family zinc finger 2	-1.95
12	Timp2	tissue inhibitor of metalloproteinase 2	-2.27
13	Id3	inhibitor of DNA binding 3	-1.95
14	Actn1	actinin, alpha 1	-2.11
15	Slc6a19	solute carrier family 6 (neurotransmitter transporter), memb	-2.27
16	Sox4	SRY-box containing gene 4	-2.13
17	Dntt	deoxynucleotidyltransferase, terminal	-1.82
18	Trat1	T cell receptor associated transmembrane adaptor 1	-2.04
19	Il6st	interleukin 6 signal transducer	-2.38
20	Mtss1	metastasis suppressor 1	-1.84
21	Rapgef4	Rap guanine nucleotide exchange factor (GEF) 4	-2.27
22	Chst15	carbohydrate (N-acetylgalactosamine 4-sulfate 6-O) sulfotran	-2.02
23	2610019F03Rik	RIKEN cDNA 2610019F03 gene	-2.11
24	Cd24a	CD24a antigen	-1.63
25	Ramp1	receptor (calcitonin) activity modifying protein 1	-1.66
26	Lass6	LAG1 homolog, ceramide synthase 6	-1.78
27	Gsn	gelsolin	-1.77
28	Trib2	tribbles homolog 2 (Drosophila)	-1.81
29	Ppic	peptidylprolyl isomerase C	-1.48
30	Dapl1	death associated protein-like 1	-2.11
31	Plekho1	pleckstrin homology domain containing, family O member 1	-1.52
32	Igf1r	insulin-like growth factor I receptor	-1.48
33	Tox	thymocyte selection-associated high mobility group box	-1.47
34	Lef1	lymphoid enhancer binding factor 1	-1.47
35	Gpr146	G protein-coupled receptor 146	-1.55
36	Usp28	ubiquitin specific peptidase 28	-1.76
37	Acsl3	acyl-CoA synthetase long-chain family member 3	-1.36
38	Cxxc5	CXXC finger 5	-1.48
39	Ccr7	chemokine (C-C motif) receptor 7	-1.89
40	Inadl	InaD-like (Drosophila)	-1.41
41	Sgk3	serum/glucocorticoid regulated kinase 3	-1.58
42	Sesn1	sestrin 1	-1.53
43	Plaur	plasminogen activator, urokinase receptor	-1.18
44	Tubb2b	tubulin, beta 2B	-1.3
45	Smc4	structural maintenance of chromosomes 4	-1.56

46	Afap1	actin filament associated protein 1	-1.41
47	Ralgps2	Ral GEF with PH domain and SH3 binding motif 2	-1.36
48	5730508B09Rik	RIKEN cDNA 5730508B09 gene	-1.55
49	Sell	selectin, lymphocyte	-1.52
50	Il6ra	interleukin 6 receptor, alpha	-1.21
51	Tspan32	tetraspanin 32	-1.4
52	Cyth3	cytohesin 3	-1.33
53	Pdk1	3-phosphoinositide dependent protein kinase 1	-1.47
54	St8sia1	ST8 alpha-N-acetyl-neuraminide alpha-2,8-sialyltransferase 1	-1.97
55	Slc43a2	solute carrier family 43, member 2	-1.33
56	Baz2b	bromodomain adjacent to zinc finger domain, 2B	-1.13
57	Adcy6	adenylate cyclase 6	-1.04
58	Adk	adenosine kinase	-1.28
59	Ldhb	lactate dehydrogenase B	-1.17
60	Dusp10	dual specificity phosphatase 10	-1.02
61	Rgs10	regulator of G-protein signalling 10	-1.34
62	Basp1	brain abundant, membrane attached signal protein 1	-1.02
63	Als2cl	ALS2 C-terminal like	-1.55
64	Sepp1	selenoprotein P, plasma, 1	-1.23
65	Amigo2	adhesion molecule with Ig like domain 2	-1.32
66	Treml2	triggering receptor expressed on myeloid cells-like 2	-1.44
67	Pik3ip1	phosphoinositide-3-kinase interacting protein 1	-1.27
68	Sfmbt2	Scm-like with four mbt domains 2	-1.19
69	Pecam1	platelet/endothelial cell adhesion molecule 1	-1.12
70	1190002H23Rik	RIKEN cDNA 1190002H23 gene	-1.09
71	Nipal1	NIPA-like domain containing 1	-1.14
72	Etv3	ets variant gene 3	-1.34
73	Bcl9	B-cell CLL/lymphoma 9	-1.3
74	Tubb2a	tubulin, beta 2A	-0.93
75	Lipa	lysosomal acid lipase A	-1.21
76	Usp6nl	USP6 N-terminal like	-1.27
77	Cd55	CD55 antigen	-1.32
78	Tmie	transmembrane inner ear	-1.23
79	Rras2	related RAS viral (r-ras) oncogene homolog 2	-1.22
80	Ext1	exostoses (multiple) 1	-1.17
81	Fam101b	family with sequence similarity 101, member B	-1.33
82	N4bp2	NEDD4 binding protein 2	-1.34
83	Abca3	ATP-binding cassette, sub-family A (ABC1), member 3	-1.2
84	Rab3ip	RAB3A interacting protein	-1.37
85	Ampd1	adenosine monophosphate deaminase 1	-1.41
86	Cep97	centrosomal protein 97	-1.19
87	Qser1	glutamine and serine rich 1	-1.04
88	Cd81	CD81 antigen	-0.83
89	Spred1	sprouty protein with EVH-1 domain 1, related sequence	-1.04
90	Idh2	isocitrate dehydrogenase 2 (NADP+), mitochondrial	-1.11
91	Itm2a	integral membrane protein 2A	-1.16
92	2410066E13Rik	RIKEN cDNA 2410066E13 gene	-1.19
93	Lztfl1	leucine zipper transcription factor-like 1	-1.26
94	Gria3	glutamate receptor, ionotropic, AMPA3 (alpha 3)	-1.05
95	Eng	endoglin	-1.05
96	Spnb2	spectrin beta 2	-0.96
97	H2-Oa	histocompatibility 2, O region alpha locus	-1.1
98	Scml4	sex comb on midleg-like 4 (Drosophila)	-1

99	Tcf7l2	transcription factor 7-like 2, T-cell specific, HMG-box	-0.71
100	Prkd2	protein kinase D2	-0.97
101	Zdhhc14	zinc finger, DHHC domain containing 14	-0.7
102	Slc16a5	solute carrier family 16 (monocarboxylic acid transporters),	-1.43
103	Tspan13	tetraspanin 13	-1.16
104	Gtf2ird1	general transcription factor II I repeat domain-containing 1	-0.89
105	Calcrl	calcitonin receptor-like	-1.22
106	Hdac4	histone deacetylase 5	-0.91
107	Slc16a10	solute carrier family 16 (monocarboxylic acid transporters),	-1.09
108	Tet1	tet oncogene 1	-1.39
109	Hectd2	HECT domain containing 2	-1.08
110	Bach2	BTB and CNC homology 2	-1.14
111	Kif13a	kinesin family member 13A	-0.81
112	Egr2	early growth response 2	-1.12
113	Ttc28	tetratricopeptide repeat domain 28	-0.89
114	Aff3	AF4/FMR2 family, member 3	-0.94
115	Serinc5	serine incorporator 5	-0.85
116	Spred2	sprouty-related, EVH1 domain containing 2	-1.07
117	Acvr1b	activin A receptor, type 1B	-1.14
118	Abca1	ATP-binding cassette, sub-family A (ABC1), member 1	-0.79
119	Sh3pxd2a	SH3 and PX domains 2A	-1.11
120	Nrip1	nuclear receptor interacting protein 1	-0.93
121	Tec	tec protein tyrosine kinase	-0.93
122	Tsc22d1	TSC22 domain family, member 1	-0.99
123	Slamf6	SLAM family member 6	-0.96
124	Stambpl1	STAM binding protein like 1	-0.89
125	Pacsin1	protein kinase C and casein kinase substrate in neurons 1	-1.07
126	Elovl6	ELOVL family member 6, elongation of long chain fatty acids	-0.88
127	Pou2af1	POU domain, class 2, associating factor 1	-0.96
128	Zbtb10	zinc finger and BTB domain containing 10	-0.73
129	Ttc3	tetratricopeptide repeat domain 3	-1.07
130	Nr4a1	nuclear receptor subfamily 4, group A, member 1	-0.97
131	Nab2	Ngfi-A binding protein 2	-0.92
132	Ggt1	gamma-glutamyltransferase 1	-1.21
133	Armcx2	armadillo repeat containing, X-linked 2	-1.14
134	Cpm	carboxypeptidase M	-1.26
135	Arhgef11	Rho guanine nucleotide exchange factor (GEF) 11	-0.73
136	Ramp3	receptor (calcitonin) activity modifying protein 3	-0.63
137	Art2b	ADP-ribosyltransferase 2b	-1.48
138	Cnksr3	Cnksr family member 3	-0.72
139	Ncf1	neutrophil cytosolic factor 1	-1.25
140	App	amyloid beta (A4) precursor protein	-1.04
141	Rbm38	RNA binding motif protein 38	-0.9
142	Dnmt3a	DNA methyltransferase 3A	-1.03
143	Mgst2	microsomal glutathione S-transferase 2	-1.19
144	Insr	insulin receptor	-0.89
145	Ctsl	cathepsin L	-0.93
146	Lclat1	lysocardiolipin acyltransferase 1	-1.01
147	Satb1	special AT-rich sequence binding protein 1	-0.97
148	Laptm4b	lysosomal-associated protein transmembrane 4B	-0.74
149	Plekhg2	pleckstrin homology domain containing, family G (with RhoGef	-0.86
150	Kif1b	kinesin family member 1B	-0.87
151	Mex3b	mex3 homolog B (C. elegans)	-0.92

152	Twsg1	twisted gastrulation homolog 1 (Drosophila)	-0.77
153	Sqrdl	sulfide quinone reductase-like (yeast)	-0.63
154	Rassf3	Ras association (RalGDS/AF-6) domain family member 3	-0.76
155	Ets2	E26 avian leukemia oncogene 2, 3' domain	-0.99
156	Ndrg1	N-myc downstream regulated gene 1	-0.86
157	Fam134b	family with sequence similarity 134, member B	-0.94
158	Dusp16	dual specificity phosphatase 16	-0.74
159	Pard6g	par-6 partitioning defective 6 homolog gamma (C. elegans)	-0.76
160	Atp8a1	ATPase, aminophospholipid transporter (APLT), class I, type	-1.04
161	Marcks	myristoylated alanine rich protein kinase C substrate	-0.78
162	Cyb5	cytochrome b-5	-1.08
163	Map4k2	mitogen-activated protein kinase kinase kinase kinase 2	-0.86
164	Bend5	BEN domain containing 5	-0.65
165	Ctsc	cathepsin C	-0.92
166	Pip4k2a	phosphatidylinositol-5-phosphate 4-kinase, type II, alpha	-0.94
167	Dzip1	DAZ interacting protein 1	-1.06
168	Cytip	cytohesin 1 interacting protein	-0.94
169	Hspb11	heat shock protein family B (small), member 11	-0.89
170	Cmah	cytidine monophospho-N-acetylneuraminic acid hydroxylase	-0.73
171	Irs2	insulin receptor substrate 2	-0.63
172	Irf6	interferon regulatory factor 6	-0.77
173	Pde4b	phosphodiesterase 4B, cAMP specific	-0.82
174	AI504432	expressed sequence AI504432	-0.88
175	Adamts6	a disintegrin-like and metallopeptidase (reprolysin type) wi	-0.77
176	Myb	myeloblastosis oncogene	-1.02
177	Mfhas1	malignant fibrous histiocytoma amplified sequence 1	-0.95
178	Tcf4	transcription factor 4	-0.58
179	Rnf19a	ring finger protein 19A	-0.92
180	Mettl8	methyltransferase like 8	-0.94
181	Ssbp2	single-stranded DNA binding protein 2	-0.84
182	Mpp1	membrane protein, palmitoylated	-1.03
183	Gprc5b	G protein-coupled receptor, family C, group 5, member B	-0.74
184	Tmem50b	transmembrane protein 50B	-1.11
185	Klf7	Kruppel-like factor 7 (ubiquitous)	-0.77
186	Nedd4l	neural precursor cell expressed, developmentally down-regula	-0.8
187	Dag1	dystroglycan 1	-0.83
188	Ppm1h	protein phosphatase 1H (PP2C domain containing)	-0.73
189	Egr1	early growth response 1	-0.76
190	Slfn5	schlafen 5	-0.89
191	Tcrg-V1	T-cell receptor gamma, variable 1	-0.67
192	Kdm5b	lysine (K)-specific demethylase 5B	-0.8
193	Rhebl1	Ras homolog enriched in brain like 1	-0.62
194	Tgfbr3	transforming growth factor, beta receptor III	-0.84
195	Dgkd	diacylglycerol kinase, theta	-0.8
196	A930005H10Rik	RIKEN cDNA A930005H10 gene	-0.68
197	Scamp1	secretory carrier membrane protein 1	-0.65
198	Dirc2	disrupted in renal carcinoma 2 (human)	-0.92
199	Scmh1	sex comb on midleg homolog 1	-0.64
200	Ccdc53	coiled-coil domain containing 53	-0.94
201	Tnfsf11	tumor necrosis factor (ligand) superfamily, member 11	-0.34
202	Nfix	nuclear factor I/X	-0.72
203	Tnik	TRAF2 and NCK interacting kinase	-0.76
204	Tns1	tensin 1	-0.41

205	Dusp6	dual specificity phosphatase 6	-0.97
206	Frmd4a	FERM domain containing 4A	-0.76
207	Rab4a	RAB4A, member RAS oncogene family	-0.84
208	Tlr1	toll-like receptor 1	-1.12
209	Hivep3	human immunodeficiency virus type I enhancer binding protein	-0.63
210	Eif2ak3	eukaryotic translation initiation factor 2 alpha kinase 3	-0.61
211	D130062J21Rik	RIKEN cDNA D130062J21 gene	-0.72
212	Tpcn1	two pore channel 1	-0.55
213	Rgmb	RGM domain family, member B	-0.65
214	Slc36a4	solute carrier family 36 (proton/amino acid symporter), memb	-1.01
215	Lrrc42	leucine rich repeat containing 42	-0.86
216	P2rx4	purinergic receptor P2X, ligand-gated ion channel 4	-0.65
217	Inpp4b	inositol polyphosphate-4-phosphatase, type II	-0.94
218	Dph5	DPH5 homolog (S. cerevisiae)	-0.93
219	Pank4	pantothenate kinase 4	-0.8
220	Ptch1	patched homolog 1	-0.91
221	Timeless	timeless homolog (Drosophila)	-0.73
222	Bend4	BEN domain containing 4	-0.85
223	Gstm1	glutathione S-transferase, mu 1	-0.51
224	Klhdc1	kelch domain containing 1	-0.92
225	Dapk1	death associated protein kinase 1	-0.61
226	Kcna2	potassium voltage-gated channel, shaker-related subfamily, m	-0.68
227	Inpp5f	inositol polyphosphate-5-phosphatase F	-0.73
228	2700081O15Rik	RIKEN cDNA 2700081O15 gene	-0.33
229	Dck	deoxycytidine kinase	-1.01
230	H2-DMa	histocompatibility 2, class II, locus DMa	-0.76
231	Csrp1	cysteine and glycine-rich protein 1	-0.77
232	Tfrc	transferrin receptor	-1.23
233	Hspbap1	Hspb associated protein 1	-0.74
234	Cspp1	centrosome and spindle pole associated protein 1	-0.84
235	Stk38	serine/threonine kinase 38	-0.64
236	Klf3	Kruppel-like factor 3 (basic)	-0.33
237	Galnt6	UDP-N-acetyl-alpha-D-galactosamine:polypeptide N-acetylgalac	-0.66
238	Hmgcs1	3-hydroxy-3-methylglutaryl-Coenzyme A synthase 1	-1.05
239	Dnajc6	DnaJ (Hsp40) homolog, subfamily C, member 6	-0.52
240	Piga	phosphatidylinositol glycan anchor biosynthesis, class A	-1.08
241	Boll	bol, boule-like (Drosophila)	-0.8
242	Dusp1	dual specificity phosphatase 1	0.17
243	Nsg2	neuron specific gene family member 2	-1.06
244	Ccdc28b	coiled coil domain containing 28B	-0.42
245	Rps17	ribosomal protein S17	-1.11
246	Oxct1	3-oxoacid CoA transferase 1	-0.98
247	Plekha1	pleckstrin homology domain containing, family A (phosphoinos	-0.91
248	Tlr12	toll-like receptor 12	-0.65
249	Cnr2	cannabinoid receptor 2 (macrophage)	-1.12
250	Ankrd28	ankyrin repeat domain 28	-0.73
251	Daf2	decay accelerating factor 2	-0.5
252	Slc12a7	solute carrier family 12, member 7	-0.74
253	Pitpnm2	phosphatidylinositol transfer protein, membrane-associated 2	-0.68
254	Ccdc126	coiled-coil domain containing 126	-0.74
255	Lypd6b	LY6/PLAUR domain containing 6B	-1.01
256	Ggta1	glycoprotein galactosyltransferase alpha 1, 3	-0.83
257	4932438A13Rik	RIKEN cDNA 4932438A13 gene	-0.68

258	Egr3	early growth response 3	-0.75
259	Tcp11l2	t-complex 11 (mouse) like 2	-0.68
260	Setd7	SET domain containing (lysine methyltransferase) 7	-0.72
261	Ap1ar	adaptor-related protein complex 1 associated regulatory prot	-0.81
262	Tanc1	tetratricopeptide repeat, ankyrin repeat and coiled-coil con	-0.6
263	Kremen1	kringle containing transmembrane protein 1	-0.6
264	Rasgrp1	RAS guanyl releasing protein 1	-0.72
265	Eml5	echinoderm microtubule associated protein like 5	-0.79
266	Ccm2	cerebral cavernous malformation 2 homolog (human)	-0.61
267	Arhgap29	Rho GTPase activating protein 29	-0.56
268	Add3	adducin 3 (gamma)	-0.57
269	Rpl31	ribosomal protein L31	-1.1
270	Sik1	salt inducible kinase 1	-0.59
271	Tmc6	transmembrane channel-like gene family 6	-0.62
272	Slc35e2	solute carrier family 35, member E2	-0.83
273	Rhoh	ras homolog gene family, member H	-0.62
274	Depdc1b	DEP domain containing 1B	-0.82
275	Aldh2	aldehyde dehydrogenase 2, mitochondrial	-0.4
276	Rcan3	regulator of calcineurin 3	-0.6
277	Smad1	MAD homolog 1 (Drosophila)	-0.87
278	Casd1	CAS1 domain containing 1	-0.96
279	Cd2ap	CD2-associated protein	-0.77
280	Plcxd2	phosphatidylinositol-specific phospholipase C, X domain cont	-0.75
281	Gk5	glycerol kinase 5 (putative)	-0.63
282	Lbh	limb-bud and heart	-0.71
283	Cyp39a1	cytochrome P450, family 39, subfamily a, polypeptide 1	-0.32
284	Ptpla	protein tyrosine phosphatase-like (proline instead of cataly	-0.81
285	Pole2	polymerase (DNA directed), epsilon 2 (p59 subunit)	-0.98
286	Zfp36	zinc finger protein 36	-0.54
287	Jarid2	jumonji, AT rich interactive domain 2	-0.66
288	Matk	megakaryocyte-associated tyrosine kinase	-0.56
289	Pcsk1	proprotein convertase subtilisin/kexin type 1	-0.38
290	Ypel2	yippee-like 2 (Drosophila)	-0.45
291	Sipa1l1	signal-induced proliferation-associated 1 like 1	-0.82
292	Abl2	v-abl Abelson murine leukemia viral oncogene homolog 2 (arg,	-0.5
293	Fntb	farnesyltransferase, CAAX box, beta	-0.82
294	Rtp4	receptor transporter protein 4	-1.19
295	Stt3b	STT3, subunit of the oligosaccharyltransferase complex, homo	-0.84
296	Myst4	MYST histone acetyltransferase monocytic leukemia 4	-0.58
297	Cep68	centrosomal protein 68	-0.78
298	Klhdc2	kelch domain containing 2	-1.05
299	Ifi27l2a	interferon, alpha-inducible protein 27 like 2A	-1.06
300	Atp6v1d	ATPase, H+ transporting, lysosomal V1 subunit D	-0.74
301	Eps8l1	EPS8-like 1	-0.46
302	Slc44a2	solute carrier family 44, member 2	-0.72
303	Arrb1	arrestin, beta 1	-0.74
304	Fos	FBJ osteosarcoma oncogene	0.11
305	Tnfaip8l1	tumor necrosis factor, alpha-induced protein 8-like 1	-0.69
306	Klf13	Kruppel-like factor 13	-0.43
307	Rictor	RPTOR independent companion of MTOR, complex 2	-0.65
308	Chd2	chromodomain helicase DNA binding protein 2	-0.51
309	Tbcel	tubulin folding cofactor E-like	-0.82
310	Ugcg	UDP-glucose ceramide glucosyltransferase	-0.82

311	Klf2	Kruppel-like factor 2 (lung)	-0.34
312	Slc17a9	solute carrier family 17, member 9	-0.5
313	Txk	TXK tyrosine kinase	-0.51
314	Cux1	cut-like homeobox 1	-0.37
315	Snx13	sorting nexin 13	-0.61
316	Mettl9	methyltransferase like 9	-0.8
317	Slc30a4	solute carrier family 30 (zinc transporter), member 4	-0.68
318	Mgat5	mannoside acetylglucosaminyltransferase 5	-0.77
319	Orai2	ORAI calcium release-activated calcium modulator 2	-0.52
320	Tbxa2r	thromboxane A2 receptor	-0.7
321	Zfp422	zinc finger protein 422	-0.86
322	Snn	stannin	-0.31
323	Abcc4	ATP-binding cassette, sub-family C (CFTR/MRP), member 4	-0.67
324	Fnbp1l	formin binding protein 1-like	-0.39
325	Oas2	2'-5' oligoadenylate synthetase 2	-0.97
326	Jun	Jun oncogene	0.59
327	Thada	thyroid adenoma associated	-0.45
328	Cyth1	cytohesin 1	-0.37
329	Apex1	apurinic/apyrimidinic endonuclease 1	-1.05
330	Ggt5	gamma-glutamyltransferase 5	-0.51
331	Pik3c2a	phosphatidylinositol 3-kinase, C2 domain containing, alpha p	-0.78
332	Wdr26	WD repeat domain 26	-0.5
333	Npc2	Niemann Pick type C2	-0.85
334	Nlk	nemo like kinase	-0.94
335	Tmem108	transmembrane protein 108	-0.36
336	Rasgrp2	RAS, guanyl releasing protein 2	-0.26
337	Cyp2s1	cytochrome P450, family 2, subfamily s, polypeptide 1	-0.54
338	Sla2	Src-like-adaptor 2	-0.7
339	Tmeff1	transmembrane protein with EGF-like and two follistatin-like	-0.37
340	Gnb4	guanine nucleotide binding protein (G protein), beta 4	-0.52
341	Ikbke	inhibitor of kappaB kinase epsilon	-0.72
342	Ube2h	ubiquitin-conjugating enzyme E2H	-0.64
343	Rnaset2a	ribonuclease T2A	-0.51
344	Zfp281	zinc finger protein 281	-0.89
345	Cd69	CD69 antigen	-0.72
346	Arid5b	AT rich interactive domain 5B (MRF1-like)	-0.46
347	Wdr12	WD repeat domain 12	-0.78
348	Dyrk2	dual-specificity tyrosine-(Y)-phosphorylation regulated kina	-0.62
349	Gch1	GTP cyclohydrolase 1	-0.55
350	Ube2r2	ubiquitin-conjugating enzyme E2R 2	-0.68
351	Dpp4	dipeptidylpeptidase 4	-0.83
352	Trim59	tripartite motif-containing 59	-0.77
353	Fam65b	family with sequence similarity 65, member B	-0.51
354	Camk4	calcium/calmodulin-dependent protein kinase IV	-0.74
355	Ppm1l	protein phosphatase 1 (formerly 2C)-like	-0.26
356	Adam6b	a disintegrin and metallopeptidase domain 6B	-0.67
357	Atp6ap2	ATPase, H+ transporting, lysosomal accessory protein 2	-0.95
358	Tgfbr1	transforming growth factor, beta receptor I	-0.66
359	Cep57l1	centrosomal protein 57-like 1	-0.85
360	Tlr7	toll-like receptor 7	-0.96
361	Cd79b	CD79B antigen	-0.59
362	Camk2d	calcium/calmodulin-dependent protein kinase II, beta	-0.53
363	Cnga1	cyclic nucleotide gated channel alpha 1	-0.8

364	Kcna3	potassium voltage-gated channel, shaker-related subfamily, m	-0.48
365	Mtap7	microtubule-associated protein 7	-0.46
366	Atxn10	ataxin 10	-0.76
367	Dnahc8	dynein, axonemal, heavy chain 8	-0.49
368	Prps1	phosphoribosyl pyrophosphate synthetase 1	-0.72
369	Usp3	ubiquitin specific peptidase 3	-0.51
370	Abtb2	ankyrin repeat and BTB (POZ) domain containing 2	-0.75
371	Rbm33	RNA binding motif protein 33	-0.49
372	Tfdp2	transcription factor Dp 2	-0.59
373	Minpp1	multiple inositol polyphosphate histidine phosphatase 1	-0.73
374	Acsf2	acyl-CoA synthetase family member 2	-0.74
375	1700017B05Rik	RIKEN cDNA 1700017B05 gene	-0.4
376	Sft2d2	SFT2 domain containing 2	-0.66
377	Zbtb20	zinc finger and BTB domain containing 20	-0.61
378	Trp53inp1	transformation related protein 53 inducible nuclear protein	-0.69
379	Trim25	tripartite motif-containing 25	-0.85
380	Bdh1	3-hydroxybutyrate dehydrogenase, type 1	-0.68
381	Phtf2	putative homeodomain transcription factor 2	-0.85
382	Znrf1	zinc and ring finger 1	-0.61
383	Fbxo32	F-box protein 32	-0.52
384	A630038E17Rik	RIKEN cDNA A630038E17 gene	-0.31
385	Ube2d2	ubiquitin-conjugating enzyme E2D 2	-0.55
386	Ndrg2	N-myc downstream regulated gene 2	-0.52
387	Frat2	frequently rearranged in advanced T-cell lymphomas 2	-0.31
388	Gramd4	GRAM domain containing 4	-0.43
389	Abcg2	ATP-binding cassette, sub-family G (WHITE), member 2	-0.56
390	Ttc27	tetratricopeptide repeat domain 27	-0.84
391	Pag1	phosphoprotein associated with glycosphingolipid microdomain	-0.61
392	Tsc22d3	TSC22 domain family, member 3	-0.17
393	37680	membrane-associated ring finger (C3HC4) 7	-0.6
394	Sorcs2	sortilin-related VPS10 domain containing receptor 2	-0.34
395	Ralb	v-ral simian leukemia viral oncogene homolog B (ras related)	-0.49
396	Zfp354c	zinc finger protein 354C	-0.55
397	Zeb1	zinc finger E-box binding homeobox 1	-0.56
398	Mapk8	mitogen-activated protein kinase 8	-0.67
399	Thra	thyroid hormone receptor alpha	-0.52
400	Rcsd1	RCSD domain containing 1	-0.56
401	Clcf1	cardiotrophin-like cytokine factor 1	-0.56
402	Prps2	phosphoribosyl pyrophosphate synthetase 2	-0.92
403	Ehd1	EH-domain containing 1	-1.09
404	Mcoln3	mucolipin 3	-0.72
405	BC057079	cDNA sequence BC057079	-0.65
406	4933439F18Rik	RIKEN cDNA 4933439F18 gene	-0.86
407	Lamp2	lysosomal-associated membrane protein 2	-0.74
408	Sema4b	sema domain, immunoglobulin domain (Ig), transmembrane domai	-0.58
409	Arhgap39	Rho GTPase activating protein 39	-0.69
410	Lrrc1	leucine rich repeat containing 1	-0.61
411	Slc35b3	solute carrier family 35, member B3	-0.88
412	Ldlr	low density lipoprotein receptor	-0.62
413	Cenpa	centromere protein A	-0.55
414	Gltp	glycolipid transfer protein	-0.71
415	Dgka	diacylglycerol kinase, alpha	-0.57
416	Fry	furry homolog (Drosophila)	-0.44

417	Fam3c	family with sequence similarity 3, member C	-0.71
418	Tbl1x	transducin (beta)-like 1 X-linked	-0.77
419	Ddx60	DEAD (Asp-Glu-Ala-Asp) box polypeptide 60	-0.77
420	Dhx40	DEAH (Asp-Glu-Ala-His) box polypeptide 40	-0.35
421	Add1	adducin 1 (alpha)	-0.55
422	Top2b	topoisomerase (DNA) II beta	-0.46
423	Sall2	sal-like 2 (Drosophila)	-0.29
424	Rsad2	radical S-adenosyl methionine domain containing 2	-0.85
425	Appl2	adaptor protein, phosphotyrosine interaction, PH domain and	-0.8
426	Ssbp3	single-stranded DNA binding protein 3	-0.52
427	Slc25a27	solute carrier family 25, member 27	-0.4
428	Camsap1l1	calmodulin regulated spectrin-associated protein 1-like 1	-0.39
429	Rnf144a	ring finger protein 144A	-0.42
430	Tdrkh	tudor and KH domain containing protein	-0.57
431	Rps6ka2	ribosomal protein S6 kinase, polypeptide 2	-0.39
432	Nucb2	nucleobindin 2	-0.45
433	Btla	B and T lymphocyte associated	-0.75
434	Lrp6	low density lipoprotein receptor-related protein 6	-0.48
435	Hsdl1	hydroxysteroid dehydrogenase like 1	-0.73
436	Kras	v-Ki-ras2 Kirsten rat sarcoma viral oncogene homolog	-0.7
437	Bace1	beta-site APP cleaving enzyme 1	-0.83
438	Pdlim1	PDZ and LIM domain 1 (elfin)	-0.42
439	Tuba1b	tubulin, alpha 1B	-0.73
440	Setx	senataxin	-0.63
441	Qprt	quinolinate phosphoribosyltransferase	-0.28
442	Btf3l4	basic transcription factor 3-like 4	-0.76
443	Mcm7	minichromosome maintenance deficient 7 (S. cerevisiae)	-0.66
444	Insig1	insulin induced gene 1	-0.64
445	Lpcat3	lysophosphatidylcholine acyltransferase 3	-0.66
446	Map4k4	mitogen-activated protein kinase kinase kinase kinase 4	-0.6
447	Ddit4	DNA-damage-inducible transcript 4	-0.87
448	Crlf3	cytokine receptor-like factor 3	-0.5
449	Maoa	monoamine oxidase A	-0.29
450	Dopey1	dopey family member 1	-0.56
451	Fxyd7	FXYD domain-containing ion transport regulator 7	-0.21
452	Folr4	folate receptor 4 (delta)	-0.45
453	Il21r	interleukin 21 receptor	-0.55
454	Rnf145	ring finger protein 145	-0.67
455	Angptl1	angiopoietin-like 1	-0.37
456	Bhlhb9	basic helix-loop-helix domain containing, class B9	-0.7
457	Rragd	Ras-related GTP binding D	-0.42
458	Tmem120b	transmembrane protein 120B	-0.36
459	Fbxl20	F-box and leucine-rich repeat protein 20	-0.56
460	Peli1	pellino 1	-0.67
461	Tuba1a	tubulin, alpha 1A	-0.47
462	Pcbp3	poly(rC) binding protein 3	-0.18
463	Scaper	S phase cyclin A-associated protein in the ER	-0.5
464	Ccdc109b	coiled-coil domain containing 109B	-0.31
465	Cdk2	cyclin-dependent kinase 2	-0.39
466	Suv39h1	suppressor of variegation 3-9 homolog 1 (Drosophila)	-0.58
467	Kif3a	kinesin family member 3A	-0.27
468	Tor1aip2	torsin A interacting protein 2	-0.72
469	Tet3	tet oncogene family member 3	-0.5

470	Kcnn4	potassium intermediate/small conductance calcium-activated c	-0.58
471	Zdhhc20	zinc finger, DHHC domain containing 11	-0.73
472	Nhsl1	NHS-like 1	-0.37
473	Pdgfrb	platelet derived growth factor receptor, beta polypeptide	-0.19
474	2810410L24Rik	RIKEN cDNA 2810410L24 gene	-0.13
475	Fam169b	family with sequence similarity 169, member B	-0.83
476	Pnpla8	patatin-like phospholipase domain containing 8	-0.63
477	Ppm1b	protein phosphatase 1B, magnesium dependent, beta isoform	-0.66
478	Tmem131	transmembrane protein 131	-0.54
479	Tnfrsf13b	tumor necrosis factor receptor superfamily, member 13b	-0.49
480	Pcgf5	polycomb group ring finger 5	-0.74
481	Dip2b	DIP2 disco-interacting protein 2 homolog B (Drosophila)	-0.46
482	Fads1	fatty acid desaturase 1	-0.64
483	Grk6	G protein-coupled receptor kinase 6	-0.58
484	Gpatch8	G patch domain containing 8	-0.7
485	Lrig1	leucine-rich repeats and immunoglobulin-like domains 1	-0.51
486	Itk	IL2-inducible T-cell kinase	-0.05
487	Ager	advanced glycosylation end product-specific receptor	-0.11
488	Smad4	MAD homolog 4 (Drosophila)	-0.49
489	Csrnp2	cysteine-serine-rich nuclear protein 2	-0.44
490	Ube4b	ubiquitination factor E4B, UFD2 homolog (S. cerevisiae)	-0.43
491	Mid1	midline 1	-0.58
492	Arf2	ADP-ribosylation factor 2	-0.33
493	Akap13	A kinase (PRKA) anchor protein 13	-0.25
494	Tmem86a	transmembrane protein 86A	-0.18
495	Zfp238	zinc finger protein 238	-0.7
496	D18Ertd653e	DNA segment, Chr 18, ERATO Doi 653, expressed	-0.46
497	Bzw2	basic leucine zipper and W2 domains 2	-0.54
498	Mpp7	membrane protein, palmitoylated 7 (MAGUK p55 subfamily membe	-0.6
499	Tubb3	tubulin, beta 3	-0.22
500	Rnf213	ring finger protein 213	-0.68
501	Tubb5	tubulin, beta 5	-0.59
502	Osbpl8	oxysterol binding protein-like 8	-0.57
503	Pde3b	phosphodiesterase 3B, cGMP-inhibited	-0.76
504	Tmem57	transmembrane protein 57	-0.49
505	Ralgps1	Ral GEF with PH domain and SH3 binding motif 1	-0.43
506	Mbtd1	mbt domain containing 1	-0.55
507	Plscr3	phospholipid scramblase 3	-0.55
508	Phf20l1	PHD finger protein 20-like 1	-0.46
509	Pgpep1l	pyroglutamyl-peptidase I-like	-0.45
510	Sesn3	sestrin 3	-0.76
511	Vamp1	vesicle-associated membrane protein 1	-0.48
512	5830411N06Rik	RIKEN cDNA 5830411N06 gene	-0.42
513	Klhl24	kelch-like 24 (Drosophila)	-0.61
514	Txnrd3	thioredoxin reductase 3	-0.36
515	Sbk1	SH3-binding kinase 1	-0.48
516	2510009E07Rik	RIKEN cDNA 2510009E07 gene	-0.22
517	Mical3	microtubule associated monoxygenase, calponin and LIM domain	-0.45
518	Tnfrsf14	tumor necrosis factor receptor superfamily, member 14 (herpe	-0.48
519	Celsr2	cadherin, EGF LAG seven-pass G-type receptor 2 (flamingo hom	-0.28
520	Ptk2	PTK2 protein tyrosine kinase 2	-0.33
521	Osbpl9	oxysterol binding protein-like 9	-0.55

522	Zfp318	zinc finger protein 318	-0.62
523	Glb1	galactosidase, beta 1	-0.44
524	Phlpp1	PH domain and leucine rich repeat protein phosphatase 1	-0.36
525	Rassf2	Ras association (RalGDS/AF-6) domain family member 2	-0.51
526	Egln3	EGL nine homolog 3 (C. elegans)	-0.6
527	Pepd	peptidase D	-0.71
528	Ccnjl	cyclin J-like	-0.32
529	Magi3	membrane associated guanylate kinase, WW and PDZ domain cont	-0.47
530	Prickle1	prickle homolog 1 (Drosophila)	-0.34
531	Mast4	microtubule associated serine/threonine kinase family member	-0.35
532	Snx30	sorting nexin family member 30	-0.36
533	3110057O12Rik	RIKEN cDNA 3110057O12 gene	-0.55
534	Slc25a23	solute carrier family 25 (mitochondrial carrier; phosphate c	-0.33
535	Lif	leukemia inhibitory factor	-0.11
536	Gbp6	guanylate binding protein 6	-0.78
537	Msrb2	methionine sulfoxide reductase B2	-0.34
538	Srpk2	serine/arginine-rich protein specific kinase 2	-0.46
539	Slc44a1	solute carrier family 44, member 1	-0.41
540	Stx2	syntaxin 2	-0.7
541	Rab6b	RAB6B, member RAS oncogene family	-0.43
542	Smc6	structural maintenance of chromosomes 6	-0.67
543	6720463M24Rik	RIKEN cDNA 6720463M24 gene	-0.59
544	Itpr2	inositol 1,4,5-triphosphate receptor 2	-0.22
545	Abcc5	ATP-binding cassette, sub-family C (CFTR/MRP), member 5	-0.39
546	Zdhhc17	zinc finger, DHHC domain containing 17	-0.56
547	Mcmbp	MCM (minichromosome maintenance deficient) binding protein	-0.48
548	Med13l	mediator complex subunit 13-like	-0.35
549	Spata6	spermatogenesis associated 6	-0.58
550	Setd1b	SET domain containing 1B	-0.14
551	Patz1	POZ (BTB) and AT hook containing zinc finger 1	-0.51
552	Asah2	N-acylsphingosine amidohydrolase 2	-0.43
553	Znrf3	zinc and ring finger 3	-0.4
554	Csrnp1	cysteine-serine-rich nuclear protein 1	-0.4
555	Rev3l	REV3-like, catalytic subunit of DNA polymerase zeta RAD54 li	-0.61
556	Gbp2	guanylate binding protein 2	-0.56
557	Arnt	aryl hydrocarbon receptor nuclear translocator	-0.46
558	Dhcr24	24-dehydrocholesterol reductase	-0.52
559	Gtf2i	general transcription factor II I	-0.09
560	Msh6	mutS homolog 6 (E. coli)	-0.62
561	Fam65a	family with sequence similarity 65, member A	-0.21
562	Cyp51	cytochrome P450, family 51	-0.66
563	Zcchc12	zinc finger, CCHC domain containing 12	-0.27
564	Msi2	Musashi homolog 2 (Drosophila)	-0.51
565	Pogz	pogo transposable element with ZNF domain	-0.61
566	Zfp286	zinc finger protein 286	-0.38
567	Vangl2	vang-like 2 (van gogh, Drosophila)	-0.25
568	Fam158a	family with sequence similarity 158, member A	-0.59
569	Lrrc8a	leucine rich repeat containing 8A	-0.45
570	Ndrg3	N-myc downstream regulated gene 3	-0.48
571	Hmgn1	high mobility group nucleosomal binding domain 1	-0.45
572	Syde1	synapse defective 1, Rho GTPase, homolog 1 (C. elegans)	-0.45
573	Vps37b	vacuolar protein sorting 37B (yeast)	-0.54

574	Asap1	ArfGAP with SH3 domain, ankyrin repeat and PH domain1	-0.68
575	Mlec	malectin	-0.43
576	Ece2	endothelin converting enzyme 2	-0.71
577	Parp9	poly (ADP-ribose) polymerase family, member 9	-0.7
578	Chd6	chromodomain helicase DNA binding protein 6	-0.47
579	Nsmce1	non-SMC element 1 homolog (S. cerevisiae)	-0.4
580	Nfkbiz	nuclear factor of kappa light polypeptide gene enhancer in B	-0.16
581	Prkch	protein kinase C, eta	-0.7
582	Cenpj	centromere protein J	-0.53
583	B630005N14Rik	RIKEN cDNA B630005N14 gene	-0.7
584	Znrf2	zinc and ring finger 2	-0.31
585	Fbxl3	F-box and leucine-rich repeat protein 3	-0.44
586	Plac8	placenta-specific 8	-0.27
587	Ighm	immunoglobulin heavy constant mu	-0.22
588	Rapgef6	Rap guanine nucleotide exchange factor (GEF) 6	-0.58
589	Wnt5b	wingless-related MMTV integration site 5B	-0.2
590	Ano6	anoctamin 6	-0.4
591	Arhgap15	Rho GTPase activating protein 15	-0.21
592	Zfp386	zinc finger protein 386 (Kruppel-like)	-0.45
593	Numb	numb gene homolog (Drosophila)	-0.59
594	Brms1l	breast cancer metastasis-suppressor 1-like	-0.76
595	Irf9	interferon regulatory factor 9	-0.62
596	Rmnd5a	required for meiotic nuclear division 5 homolog A (S. cerevi	-0.57
597	Lpp	LIM domain containing preferred translocation partner in lip	-0.57
598	Irgm2	immunity-related GTPase family M member 2	-0.88
599	Ezr	ezrin	-0.39
600	Rhoa	ras homolog gene family, member A	-0.4
601	Siah1a	seven in absentia 1A	-0.63
602	Mbnl2	muscleblind-like 2	-0.66
603	Acpp	acid phosphatase, prostate	-0.65
604	Larp4	La ribonucleoprotein domain family, member 4	-0.72
605	Tmem71	transmembrane protein 71	-0.41
606	Hook1	hook homolog 1 (Drosophila)	-0.74
607	Iigp1	interferon inducible GTPase 1	-0.75
608	Pcca	propionyl-Coenzyme A carboxylase, alpha polypeptide	-0.59
609	Glcci1	glucocorticoid induced transcript 1	-0.44
610	Pld3	phospholipase D family, member 3	-0.38
611	Fnip1	folliculin interacting protein 1	-0.62
612	Rere	arginine glutamic acid dipeptide (RE) repeats	-0.44
613	Gm885	predicted gene 885	-0.81
614	A230046K03Rik	RIKEN cDNA A230046K03 gene	-0.66
615	Atxn2	ataxin 2	-0.61
616	Pbx2	pre B-cell leukemia transcription factor 2	-0.33
617	Dcaf6	DDB1 and CUL4 associated factor 6	-0.41
618	Tcf12	transcription factor 12	-0.62
619	Hpcal1	hippocalcin-like 1	-0.44
620	Ipcef1	interaction protein for cytohesin exchange factors 1	-0.51
621	Cnst	consortin, connexin sorting protein	-0.48
622	Pde6d	phosphodiesterase 6D, cGMP-specific, rod, delta	-0.43
623	Lactb	lactamase, beta	-0.67
624	Mpzl1	myelin protein zero-like 1	-0.12
625	Mpp5	membrane protein, palmitoylated 5 (MAGUK p55 subfamily membe	-0.47

626	Arid4a	AT rich interactive domain 4A (RBP1-like)	-0.58
627	Strbp	spermatid perinuclear RNA binding protein	-0.69
628	Zyg11b	zyg-ll homolog B (C. elegans)	-0.41
629	Lphn1	latrophilin 1	-0.34
630	Igsf3	immunoglobulin superfamily, member 3	-0.19
631	Ptov1	prostate tumor over expressed gene 1	-0.17
632	Dlg3	discs, large homolog 3 (Drosophila)	-0.29
633	Etnk1	ethanolamine kinase 1	-0.66
634	Cdc14b	CDC14 cell division cycle 14 homolog B (S. cerevisiae)	-0.53
635	Ablim1	actin-binding LIM protein 1	-0.4
636	Kcnj16	potassium inwardly-rectifying channel, subfamily J, member 1	-0.16
637	Brip1	BRCA1 interacting protein C-terminal helicase 1	-0.33
638	Atp13a3	ATPase type 13A3	-0.57
639	Zfp516	zinc finger protein 516	-0.31
640	Tbc1d15	TBC1 domain family, member 15	-0.55
641	Ccdc28a	coiled-coil domain containing 28A	-0.55
642	Slc11a2	solute carrier family 11 (proton-coupled divalent metal ion	-0.55
643	Wdr46	WD repeat domain 46	-0.49
644	Atp6v0d1	ATPase, H+ transporting, lysosomal V0 subunit D1	-0.51
645	Cep350	centrosomal protein 350	-0.33
646	Itga5	integrin alpha 5 (fibronectin receptor alpha)	-0.24
647	Abl1	c-abl oncogene 1, non-receptor tyrosine kinase	-0.47
648	Rps6ka5	ribosomal protein S6 kinase, polypeptide 5	-0.38
649	Pccb	propionyl Coenzyme A carboxylase, beta polypeptide	-0.41
650	Hbp1	high mobility group box transcription factor 1	-0.52
651	Smchd1	SMC hinge domain containing 1	-0.65
652	Eef2k	eukaryotic elongation factor-2 kinase	-0.42
653	Sh3bp5	SH3-domain binding protein 5 (BTK-associated)	-0.35
654	Men1	multiple endocrine neoplasia 1	-0.37
655	Ctse	cathepsin E	0.11
656	Gpd2	glucose-6-phosphate dehydrogenase 2	-0.33
657	Eif4e3	eukaryotic translation initiation factor 4E member 3	-0.26
658	Ascc3	activating signal cointegrator 1 complex subunit 3	-0.39
659	Tnrc6c	trinucleotide repeat containing 6C	-0.43
660	Cep170	centrosomal protein 170	-0.51
661	Idi1	isopentenyl-diphosphate delta isomerase	-0.99
662	Trpm7	transient receptor potential cation channel, subfamily M, me	-0.49
663	Rcor1	REST corepressor 2	-0.59
664	Mpp6	membrane protein, palmitoylated 6 (MAGUK p55 subfamily membe	-0.56
665	Calcoco1	calcium binding and coiled coil domain 1	-0.24
666	Cox7a2l	cytochrome c oxidase subunit VIIa polypeptide 2-like	-0.44
667	Spo11	sporulation protein, meiosis-specific, SPO11 homolog (S. cer	-0.62
668	Glo1	glyoxalase 1	-0.72
669	Smad5	MAD homolog 5 (Drosophila)	-0.49
670	Arhgef1	Rho guanine nucleotide exchange factor (GEF) 1	-0.1
671	Polg2	polymerase (DNA directed), gamma 2, accessory subunit	-0.55
672	Hdac5	histone deacetylase 5	-0.44
673	4632428N05Rik	RIKEN cDNA 4632428N05 gene	-0.46
674	Akap2	A kinase (PRKA) anchor protein 2	-0.34
675	Il1rl2	interleukin 1 receptor-like 2	-0.57
676	Senp7	SUMO1/sentrin specific peptidase 7	-0.38
677	Tex2	testis expressed gene 2	-0.6

678	Slfn10-ps	schlafen 10, pseudogene	-0.4
679	Atp11c	ATPase, class VI, type 11C	-0.61
680	Narg2	NMDA receptor-regulated gene 2	-0.71
681	Sos1	son of sevenless homolog 1 (Drosophila)	-0.45
682	Oasl2	2'-5' oligoadenylate synthetase-like 2	-0.74
683	Ly75	lymphocyte antigen 75	-0.59
684	H2afv	H2A histone family, member V	-0.49
685	Nme4	non-metastatic cells 4, protein expressed in	-0.52
686	Aff4	AF4/FMR2 family, member 4	-0.34
687	Emb	embigin	-0.51
688	Jmy	junction-mediating and regulatory protein	-0.49
689	Hmgcr	3-hydroxy-3-methylglutaryl-Coenzyme A reductase	-0.65
690	Akap12	A kinase (PRKA) anchor protein (gravin) 12	-0.45
691	Nasp	nuclear autoantigenic sperm protein (histone-binding)	-0.63
692	Ctss	cathepsin S	-0.89
693	Vipr1	vasoactive intestinal peptide receptor 1	-0.54
694	Parp11	poly (ADP-ribose) polymerase family, member 11	-0.7
695	Rcc2	regulator of chromosome condensation 2	-0.56
696	Ccdc22	coiled-coil domain containing 22	-0.47
697	Zfp407	zinc finger protein 407	-0.47
698	Ccng2	cyclin G2	-0.56
699	Rbbp6	retinoblastoma binding protein 6	-0.48
700	Stat5a	signal transducer and activator of transcription 5A	-0.42
701	Cd247	CD247 antigen	-0.1
702	Suv420h1	suppressor of variegation 4-20 homolog 1 (Drosophila)	-0.4
703	Slc15a2	solute carrier family 15 (H+/peptide transporter), member 2	-0.82
704	Mll3	myeloid/lymphoid or mixed-lineage leukemia 3	-0.21
705	Lman2l	lectin, mannose-binding 2-like	-0.59
706	Upf2	UPF2 regulator of nonsense transcripts homolog (yeast)	-0.58
707	Zfand5	zinc finger, AN1-type domain 5	-0.58
708	Uck2	uridine-cytidine kinase 2	-0.47
709	Gpsm2	G-protein signalling modulator 2 (AGS3-like, C. elegans)	-0.52
710	Cd53	CD53 antigen	-0.47
711	Limk2	LIM motif-containing protein kinase 2	-0.41
712	Ncapd3	non-SMC condensin II complex, subunit D3	-0.55
713	Gm7120	predicted gene 7120	-0.48
714	Utrn	utrophin	-0.08
715	Inpp5a	inositol polyphosphate-5-phosphatase A	-0.45
716	Rasgrf2	RAS protein-specific guanine nucleotide-releasing factor 2	-0.47
717	Flot2	flotillin 2	-0.35
718	Sqle	squalene epoxidase	-0.6
719	Lmbr1l	limb region 1 like	-0.26
720	Arl5b	ADP-ribosylation factor-like 5B	-0.57
721	Cd1d1	CD1d1 antigen	-0.56
722	Lmo4	LIM domain only 4	-0.38
723	Ctnnbl1	catenin, beta like 1	-0.48
724	Tubb2c	tubulin, beta 2C	-0.38
725	Gabrr2	gamma-aminobutyric acid (GABA) C receptor, subunit rho 2	-0.36
726	Slc25a5	solute carrier family 25 (mitochondrial carrier, adenine nuc	-0.83
727	Stk3	serine/threonine kinase 3 (Ste20, yeast homolog)	-0.21
728	Gtf2ird2	GTF2I repeat domain containing 2	-0.33
729	Pdk2	pyruvate dehydrogenase kinase, isoenzyme 2	-0.22
730	Art4	ADP-ribosyltransferase 4	-0.39

731	Mthfd1l	methylenetetrahydrofolate dehydrogenase (NADP+ dependent) 1-	-0.59
732	Carns1	carnosine synthase 1	-0.22
733	Arl3	ADP-ribosylation factor-like 3	-0.48
734	Pygm	muscle glycogen phosphorylase	-0.25
735	Clasp2	CLIP associating protein 2	-0.39
736	4930420K17Rik	RIKEN cDNA 4930420K17 gene	-0.68
737	Trpc4ap	transient receptor potential cation channel, subfamily C, me	-0.47
738	Ikzf4	IKAROS family zinc finger 4	-0.19
739	Cnot6	CCR4-NOT transcription complex, subunit 6	-0.54
740	Cryl1	crystallin, lambda 1	-0.38
741	Fam116a	family with sequence similarity 116, member A	-0.54
742	Chrnb1	cholinergic receptor, nicotinic, beta polypeptide 1 (muscle)	-0.23
743	Frs2	fibroblast growth factor receptor substrate 2	-0.59
744	E130308A19Rik	RIKEN cDNA E130308A19 gene	-0.42
745	Atrnl1	attractin like 1	-0.39
746	Ung	uracil DNA glycosylase	-0.51
747	Zfyve21	zinc finger, FYVE domain containing 21	-0.44
748	Aff1	AF4/FMR2 family, member 1	-0.51
749	Luc7l2	LUC7-like 2 (S. cerevisiae)	-0.34
750	Tusc3	tumor suppressor candidate 3	-0.51
751	Snhg1	small nucleolar RNA host gene (non-protein coding) 1	-0.77
752	Smarca2	SWI/SNF related, matrix associated, actin dependent regulato	-0.52
753	Gltscr1	glioma tumor suppressor candidate region gene 1	-0.33
754	Msh3	mutS homolog 3 (E. coli)	-0.44
755	A630033H20Rik	RIKEN cDNA A630033H20 gene	-0.51
756	Tmem164	transmembrane protein 164	-0.36
757	A130010J15Rik	RIKEN cDNA A130010J15 gene	-0.42
758	Zfp53	zinc finger protein 53	-0.63
759	Tecpr1	tectonin beta-propeller repeat containing 1	-0.26
760	Mir17hg	MIR17 host gene 1 (non-protein coding)	-0.52
761	Lims1	LIM and senescent cell antigen-like domains 1	-0.57
762	2410042D21Rik	RIKEN cDNA 2410042D21 gene	-0.61
763	Arhgap5	Rho GTPase activating protein 5	-0.3
764	Rnf103	ring finger protein 103	-0.43
765	Rpl41	ribosomal protein L41	-0.51
766	Syde2	synapse defective 1, Rho GTPase, homolog 2 (C. elegans)	-0.4
767	Rrad	Ras-related associated with diabetes	-0.51
768	Thrap3	thyroid hormone receptor associated protein 3	-0.46
769	Cry1	cryptochrome 1 (photolyase-like)	-0.42
770	Mrrf	mitochondrial ribosome recycling factor	-0.63
771	Ccdc117	coiled-coil domain containing 117	-0.38
772	Nfkbia	nuclear factor of kappa light polypeptide gene enhancer in B	0.04
773	Emp3	epithelial membrane protein 3	-0.39
774	Phyhd1	phytanoyl-CoA dioxygenase domain containing 1	-0.29
775	Trrap	transformation/transcription domain-associated protein	-0.42
776	Enc1	ectodermal-neural cortex 1	-0.54
